# Antioxidant Activity of a New Series of Carbazole-Derived Nitrones

**DOI:** 10.3390/molecules31203619

**Published:** 2026-10-11

**Authors:** Maciej J. Rojewski, Dimitra Hadjipavlou-Litina, Iwona E. Głowacka, Jose Marco-Contelles, Dorota G. Piotrowska

**Affiliations:** 1 Bioorganic Chemistry Laboratory, Faculty of Pharmacy, Medical University of Lodz, Muszyńskiego, 90-151 Lodz, Poland; maciej.rojewski@umed.lodz.pl (M.J.R.); iwona.glowacka@umed.lodz.pl (I.E.G.); 2 Laboratory of Pharmaceutical Chemistry, School of Pharmacy, Aristotle University of Thessaloniki, 54124 Thessaloniki, Greece; hadjipav@pharm.auth.gr; 3 Laboratory of Medicinal Chemistry, Institute of General Organic Chemistry (CSIC), C/ Juan de la Cierva 3, 28006 Madrid, Spain; 43genn@gmail.com

**Keywords:** nitrones, antioxidant activities, anti-inflammatory activities, lipoxygenase inhibitors, lipid peroxidation

## Abstract

A new series of 2- and 4-substituted carbazole-derived nitrones was synthesized and evaluated for their antioxidant properties using multiple in vitro protocols, including inhibition of AAPH-induced lipid peroxidation, hydroxyl radical scavenging, ABTS^•+^–decolorization and DPPH interaction assays. Their anti-inflammatory capacity was investigated through the inhibition of soybean lipoxygenase (SLOX). Among all fourteen tested nitrones, compound **11bg** emerged as the most promising derivative, exhibiting strong inhibition of lipid peroxidation and efficient hydroxyl radical scavenging activity. Nitrone **11be** showed the highest lipoxygenase inhibition with an IC_50_ value of 0.0225 μM. Furthermore, seven of the fourteen synthesized hybrids showed strong SLOX inhibitory activity, with IC_50_ values below 30 μM.

## 1. Introduction

Free radicals play a fundamental role in maintaining physiological homeostasis [1]. The generation of reactive oxygen species (ROS) and reactive nitrogen species (RNS) is an intrinsic component of normal cellular metabolism, essential for numerous biological processes, including modulation of intracellular pH [2], regulation of cell proliferation and growth [3], activation of calcium channels mediating Ca^2+^ transport [4], and facilitation of glucose uptake in muscle tissue [5]. Despite their indispensable regulatory functions, excessive production of free radicals can disrupt cellular integrity and impair normal physiological processes [6]. To counterbalance their potentially harmful effects, organisms have evolved complex antioxidant defense systems comprising both enzymatic and non-enzymatic components [6,7]. When the equilibrium between reactive species generation and antioxidant capacity is disturbed, oxidative stress (OS) ensues. Mounting evidence implicates OS in the pathogenesis of numerous disorders, including viral infections (e.g., AIDS [8] and influenza [9]), metabolic diseases (such as diabetes [10], atherosclerosis [11], and asthma [12]), neurodegenerative conditions (including Alzheimer’s disease (AD) [13], Parkinson’s disease (PD) [14], and multiple sclerosis [15]), as well as various cancers [16].

**Figure 1:**
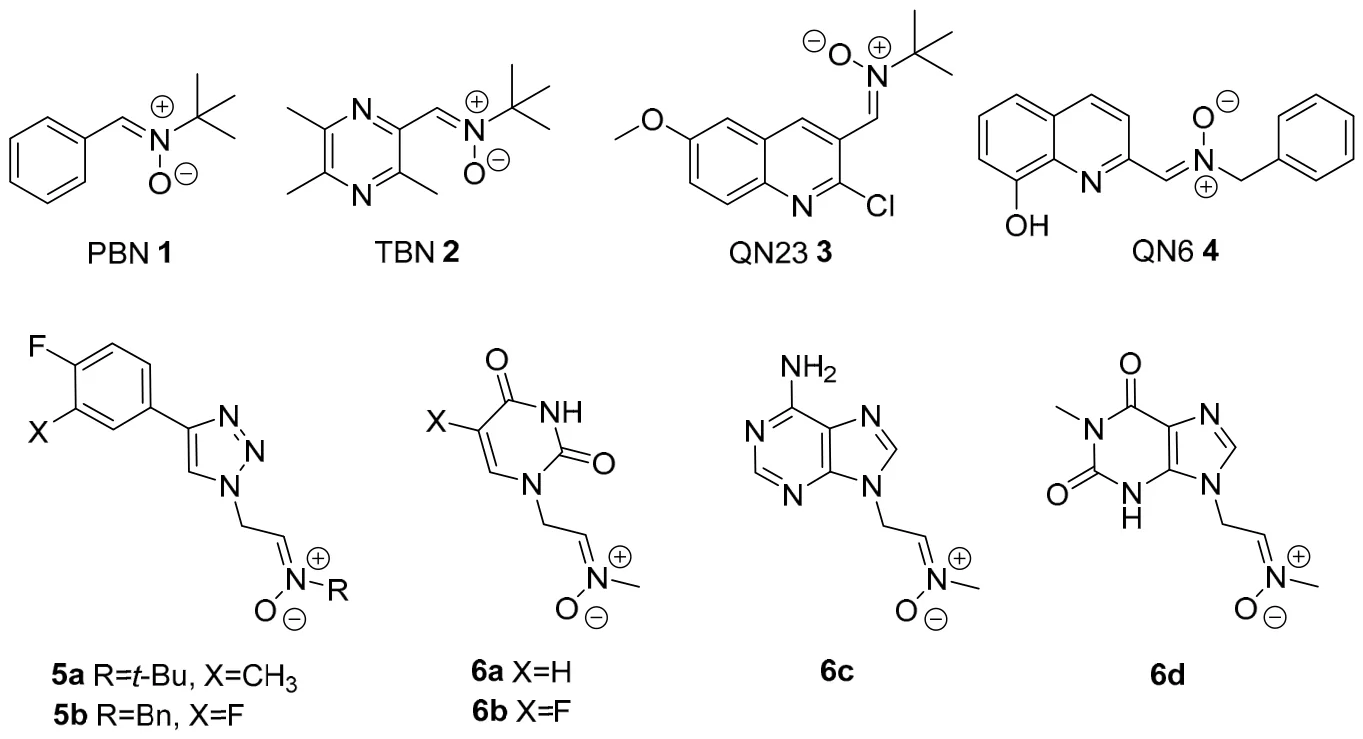
Structures of nitrones exhibiting antioxidant and neuroprotective effects.

In light of these observations, considerable research efforts over recent decades have focused on the identification and development of natural and synthetic antioxidants capable of mitigating OS through the scavenging of ROS and RNS. Among the most extensively investigated compounds are nitrones, a class of molecules demonstrating significant therapeutic potential in the management of oxidative stress-related pathologies [17]. Experimental studies have shown that PBN **1** (Figure 1) exerts protective effects by attenuating post-traumatic shock [18] and conferring neuroprotection in ischemic stroke models [19]. Likewise, quinoline-derived nitrones QN23 **3** and QN6 **4** (Figure 1) have been reported to reduce the expression of key molecular markers involved in autophagy during stroke through their antiautophagic activity [20,21]. Furthermore, TBN **2** (Figure 1) has emerged as a promising candidate for preventing or slowing neurodegenerative processes associated with PD [22]. Various other heterocyclic systems were also successfully incorporated into the nitrone structure and resulted in the identification of compounds with antioxidant potential. This research approach is well exemplified by our previous studies on 1,2,3-triazole derivatives **5** [23,24] as well as nucleobase-derived nitrones **6** [25] (Figure 1). And thus, among the series of 1,2,3-triazole-containing nitrones investigated, compound **5a** [23] and **5b** [24] have been found the most potent antioxidant agents. Furthermore, the incorporation of selected nucleobase moieties into the nitrone structure resulted in the identification of promising antioxidants, namely pyrimidinic nitrones **6a**,**b** and purinic nitrones **6c**,**d**, which exhibited superior antioxidant and neuroprotective properties compared with PBN [25].

**Figure 2:**
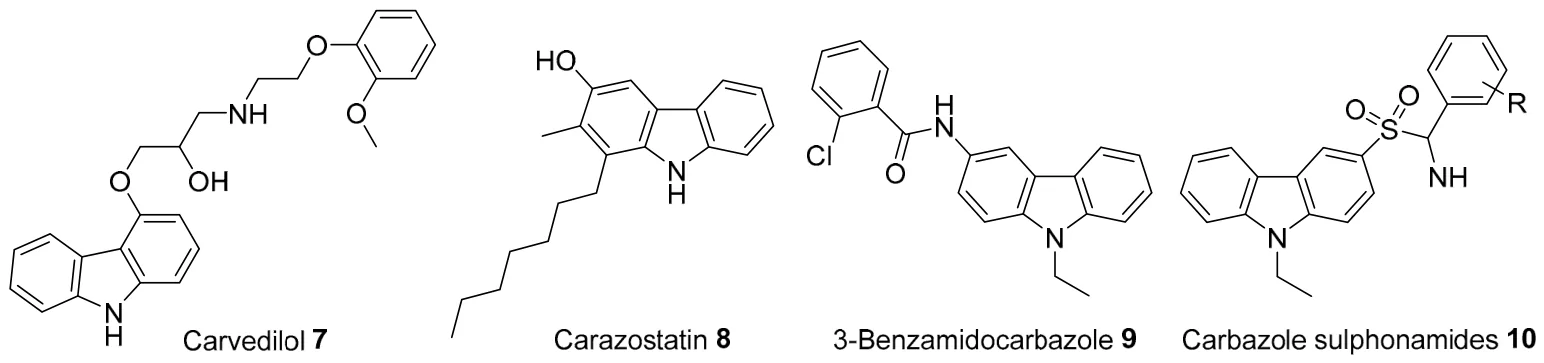
Examples of drugs containing the carbazole scaffold.

Another class of molecules that has been of continuing interest in biological and medicinal research are heterocyclic compounds, i.e., structurally diverse and pharmacologically important molecules. Among them, carbazole occupies a distinguished position owing to its unique tricyclic scaffold and broad functional versatility. Both naturally occurring and synthetically derived carbazole analogs exhibit a wide spectrum of biological activities, including anti-HIV [26], antimicrobial [27,28], antihistaminic [29], antiviral [30], anti-inflammatory [28], anticancer [31], and antioxidant effects, with particular relevance to neurodegenerative disorders such as AD [32]. The therapeutic potential of the carbazole framework is underscored by its incorporation into numerous clinically relevant agents (Figure 2). One of the most prominent examples is carvedilol **7**, a carbazole-containing drug that acts as a nonselective *β*-adrenergic receptor antagonist with well-established antihypertensive efficacy. Notably, carvedilol demonstrates exceptionally strong antioxidant activity, surpassing that of other *β*-blockers [33]. This property has been attributed, at least in part, to its ability to chelate free iron ions [34]. Another representative compound, carazostatin **8**, exhibits antioxidant activity exceeding that of α-tocopherol [35]. Further examples highlighting the pharmacological versatility of carbazole derivatives include 3-benzamidocarbazole **9**, an inhibitor of prostaglandin E_2_ (PGE_2_) production [36], as well as carbazole sulphonamides **10**, a promising novel class of antimitotic agents with potential for clinical development in the treatment of solid tumors [37]. These findings emphasize the carbazole core as a privileged structural motif in drug discovery and justify continued efforts toward the design and evaluation of new carbazole-based therapeutic candidates.

**Figure 3:**
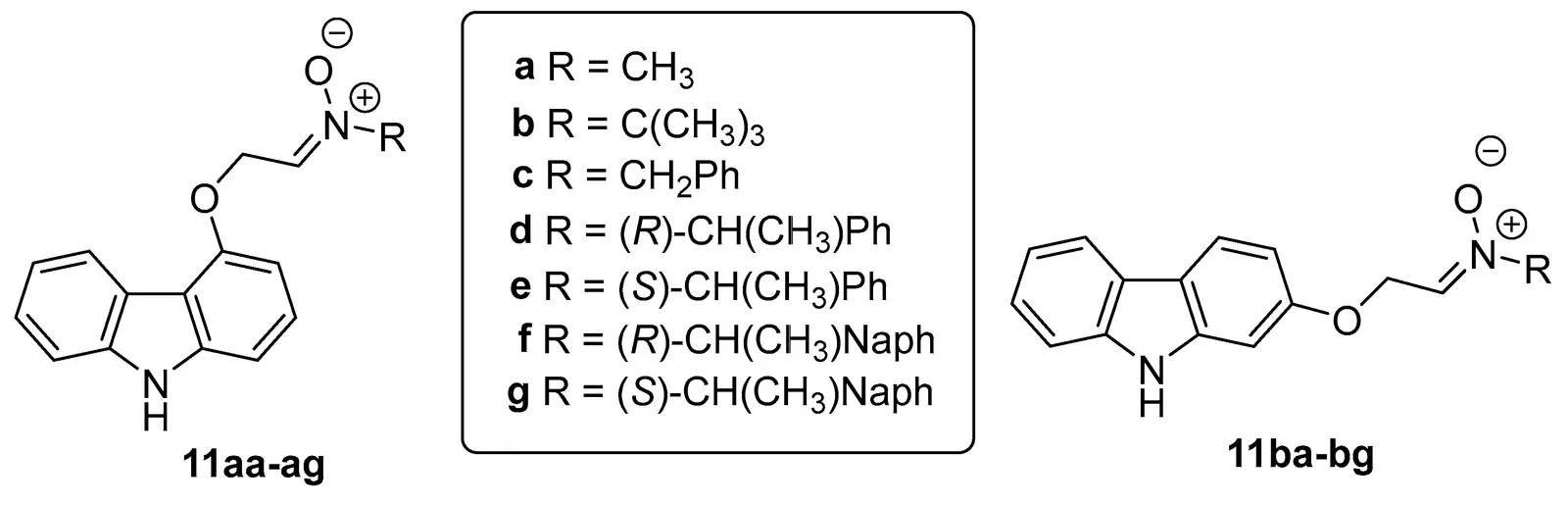
Nitrones **11aa**–**11ag** and **11ba**–**11bg** synthesized and characterized in the present work.

The strategy of enhancing the pharmacological potential of a drug by hybridization, combining two or more pharmacophores with established biological properties in one entity, is frequently applied in search for novel drug candidates [38,39]. Following this approach, the new series of compounds **11aa**–**ag** and **11ba**–**bg** have been designed by incorporating a nitrone function into the carbazole moiety (Figure 3).

## 2. Results and Discussion

### 2.1. Chemistry

**Scheme 1:**
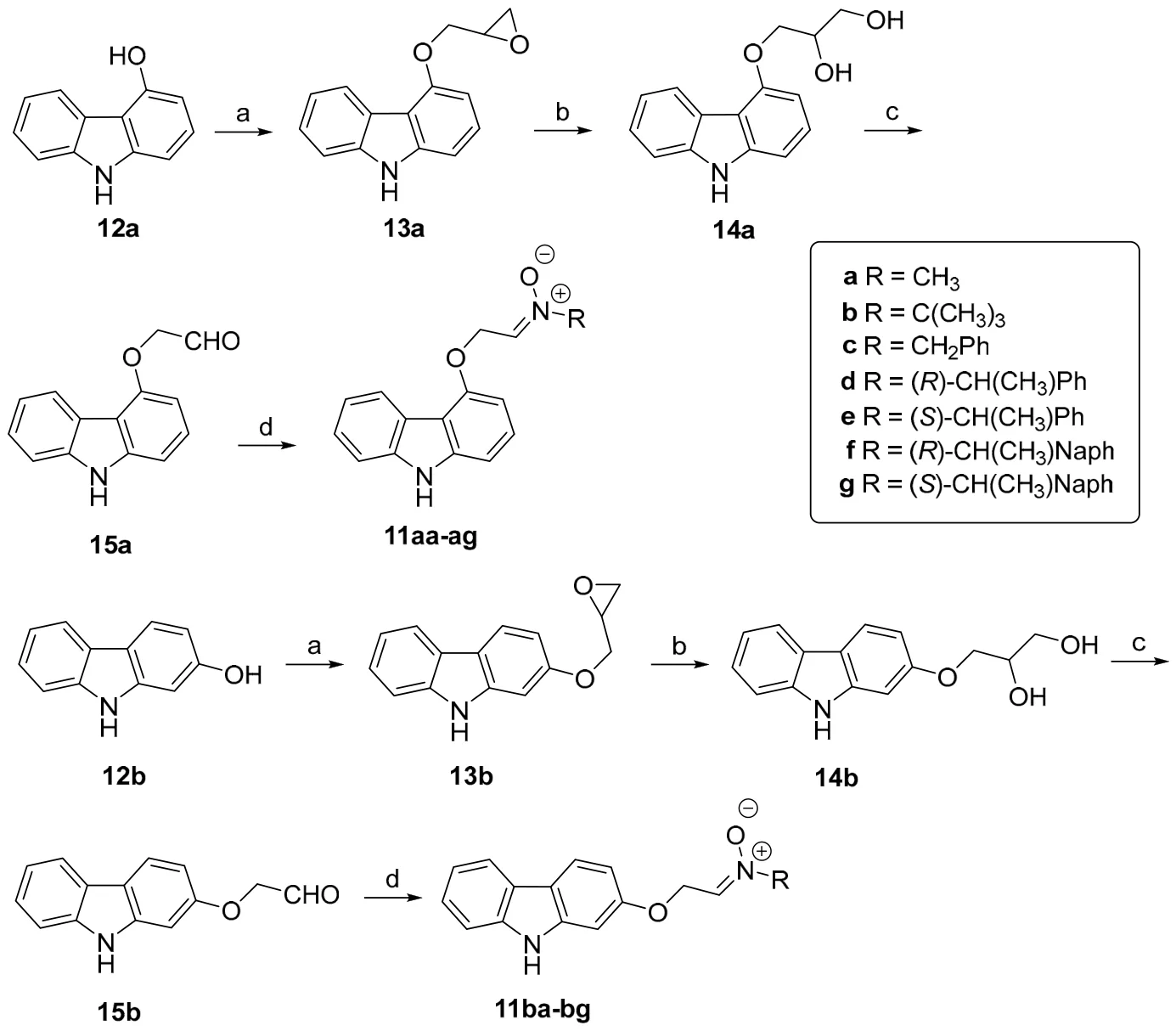
Synthesis of **11aa**–**ag** and **11ba**–**bg**. Reagents and conditions: a. epibromohydrin, 1M NaOH, DMSO, rt; b. H_2_SO_4_, water, reflux, 24 h; c. NaIO_4_, water, ethyl acetate, rt, 24 h; d. RNHOH × HCl or RNHOH × (COOH)_2_, Na_2_CO_3_, EtOH, rt, 1–5 h, 20–73% (calculated for two steps, starting from the corresponding diol **14**).

Nitrones **11aa**–**ag** and **11ba**–**bg** were prepared following the reaction sequence presented in Scheme 1. Initially, 4-hydroxycarbazole **12a** was alkylated with epibromohydrin to afford epoxide **13a** [40,41], which was subsequently converted into diol **14a** [42]. Oxidative cleavage of the diol with sodium metaperiodate in a biphasic ethyl acetate–water system [43] gave **15a** as a 17:83 mixture of corresponding aldehyde and hydrate, which was sufficiently pure to be used directly in the subsequent step without further purification. All nitrones **11aa**–**ag** were obtained in good yields by reacting compound **15a** with appropriate *N*-substituted hydroxylamines [44]. An analogous synthetic sequence was applied for the preparation of nitrones **11ba**–**bg**. In this case, compound **15b**, obtained as a 38:62 mixture of the corresponding aldehyde and hydrate, was treated with the same set of respective *N*-substituted hydroxylamines to furnish the designed products. The NMR spectra of nitrones **11aa**–**ag** and **11ba**–**bg** demonstrated that they exist exclusively as *Z*-isomers (see Supplementary Material); however, no corresponding signals attributed to other *E*-isomers that could serve as a comparison were observed, regardless of the solvent used (chloroform-*d* or DMSO-*d*_6_). This assignment was further supported by comparison with literature data, which indicate that the Z-configuration has also been observed for other acyclic nitrones [24,25,45]. Progress of the reactions and the consumption of the starting materials were monitored by TLC and finally confirmed by ^1^H NMR taken for the crude reaction mixtures.

### 2.2. In Vitro Antioxidant and Anti-Inflammatory Activity

All synthesized nitrones **11aa**–**11ag** and **11ba**–**11bg** were evaluated in vitro for their antioxidant properties, focusing on both their radical scavenging activities and their inhibition against soybean lipoxygenase (LOX) (Table 1). The antioxidant potential of the synthesized compounds was evaluated through a series of complementary assays and compared with the reference antioxidants nordihydroguaiaretic acid (NDGA) and Trolox. Owing to the multifactorial nature of OS and inflammation, the antioxidant activity of the synthesized nitrones was assessed using four distinct in vitro assays in order to obtain a comprehensive evaluation of their antioxidant profile, i.e., (a) interaction with the stable free radical 2,2-diphenyl-1-picrylhydrazyl (DPPH) [46], (b) inhibition of lipid peroxidation induced by the water-soluble azo initiator 2,2′-azobis(2-amidinopropane) dihydrochloride (AAPH) [47], (c) competition with dimethyl sulfoxide (DMSO) for hydroxyl radicals [48], and (d) scavenging activity against the cation radical ABTS^•+^ [2,2′-azinobis(3-ethylbenzothiazoline-6-sulfonic acid)] [48]. All assays were based on spectrophotometric measurements, providing rapid, simple, and reliable evaluation of the antioxidant properties of the investigated compounds. NDGA and Trolox were used as standard reference compounds in the studies for comparison reasons.

DPPH is a stable free radical widely employed in antioxidant activity assays due to its high stability and ease of use [48]. Furthermore, the DPPH assay is considered relatively independent of the polarity of the tested compounds, making it a versatile method for the evaluation of diverse antioxidant molecules. The radical scavenging activities of the investigated compounds toward the stable DPPH radical are summarized in Table 1. Among the tested compounds, nitrone **11bg** having an (*S*)-(1-naphthalen-1-yl)ethyl substituent exhibited the highest DPPH radical scavenging activity (89.7%), although its activity remained slightly lower than that of the reference antioxidant NDGA (97%). The other tested nitrones displayed low-to-moderate activities, with DPPH inhibition values ranging from 11% to 54.5%. The observed radical scavenging activity appeared to depend on both the nature of the substituent attached to the nitrogen atom and the position of the nitrone moiety within the carbazole scaffold. In general, 4-substituted carbazole nitrones demonstrated higher DPPH-reducing activity than their respective 2-substituted analogs (**11aa** vs. **11ba**, **11ab** vs. **11bb**, **11ac** vs. **11bc**, **11ad** vs. **11bd**, **11ae** vs. **11be**, **11af** vs. **11bf**). The only exception was observed for nitrones containing (*S*)-(1-naphthalen-1-yl)ethyl group, where the 2-functionalized carbazole nitrone was more active than its 4-substituted counterpart (**11bg** vs. **11ag**). No unambiguous structure–activity relationship could be established across the entire series of compounds. Nevertheless, within the series of 4-substituted carbazole nitrones, derivatives containing (*R*)-configured substituents were consistently more active than their corresponding (*S*) enantiomers [(*R*)-**11ad** vs. (*S*)-**11ae**, and (*R*)-**11af** vs. (*S*)-**11ag**]. This trend was not maintained among the 2-substituted carbazole derivatives, where (*R*)-**11bd** was more active than (*S*)-**11be**, whereas (*S*)-**11bg** exhibited higher activity than (*R*)-**11bf**. Lipophilicity did not show to influence activity. It is worth nothing that the introduction of a carbazole moiety appears to slightly affect the DPPH radical scavenging activity of the nitrones. Nitrones **11aa** (39%) and **11ba** (24.5%), both bearing a methyl group on the nitrogen atom of nitrone function, exhibited slightly higher radical scavenging activity than previously described N-methylated nucleobase-containing nitrones **6** (6–20%) (Figure 1) [25]. This trend is even more pronounced for the N-tert-butyl derivatives **11ab** (34%) and **11bb** (15%) compared to their known quinoline-containing counterpart QN 23 **3** (4%) [49].

The inhibition of lipid peroxidation (ILPO) was evaluated using the AAPH-induced lipid peroxidation assay [47], and the results are summarized in Table 1. *N*-Methyl 4-substituted carbazole nitrone **11aa**, as well as enantiomerically pure 2-functionalized carbazole-derived nitrones **11bd**, **11be**, **11bf** and **11bg** exhibited significant inhibition of lipid peroxidation (ILPO = 78.8–100%) at a concentration of 100 µM. Trolox, used as a positive control, afforded 93% inhibition under the same experimental conditions. For the achiral nitrones bearing Me, *t*-Bu and Bn groups on the nitrone nitrogen atom, the 4-functionalized carbazole derivatives (series **11a**) were generally more active than the respective 2-functionalized carbazole analogs (series **11b**) (**11aa** vs. **11ba**, **11ab** vs. **11bb**, **11ac** vs. **11bc**). In contrast, all chiral nitrones containing 1-phenylethyl [CH(Ph)Me] or 1-(naphthalen-1-yl)ethyl [CH(2-Naph)Me] displayed the opposite trend, with the 2-substituted derivatives (series **11b**) exhibiting greater activity than the corresponding 4-substituted analogs (series **11a**) [(**11bd** vs. **11ad**, **11be** vs. **11ae**, **11bf** vs. **11af**, **11bg** vs. **11ag**). Moreover, within the series of 2-functionalized carbazole derivatives (series **11b**), the correlation between the absolute configuration of the stereogenic center and lipid peroxidation inhibitory activity was observed. Specifically, nitrones containing (*R*)-configured 1-phenylethyl and (1-naphthalen-1-yl)ethyl substituents (**11bd** and **11bf**) exhibited higher activity than the corresponding (*S*) enantiomers (**11be** and **11bg**), suggesting the influence of absolute configuration of the nitrone on antioxidant performance in this assay. Again, we did not find any role for lipophilicity. While lipid peroxidation inhibition values (ILPOs) revealed that the corresponding N-methylated carbazole nitrones **11aa** (88.3%) and **11ba** (52.2%) exhibited activities comparable to those of previously reported nucleobase-derived nitrones **6** (55–92%) (Figure 1) [25], the activity of the triazole-derived nitrones **5** (Figure 1) appears to depend not only on the substituent at the nitrogen atom of nitrone function, but primarily on the aromatic substituent at the C5 position of the triazole ring, including the presence of fluorine atom(s) in the aromatic ring [23,24]. On the other hand, the observed inhibition values (ILPO) for the corresponding N-*tert*-butyl carbazole derivatives **11ab** (65.6%) and **11bb** (47.2%) highlight the significant role of the carbazole moiety in enhancing the antioxidant properties of the obtained nitrones compared with the quinoline-containing nitrone QN23 **3** (22%) [49].

Free hydroxyl radicals (^•^OH) are among the most reactive ROS, and can cause severe damage to biologically important macromolecules, including DNA, lipids, proteins and carbohydrates. Therefore, the ability of the obtained nitrones to scavenge ^•^OH radicals was considered as an important parameter in the evaluation of their antioxidant potential [48]. As shown in Table 1, the majority of the tested compounds exhibited high activity at a concentration of 100 µM. The scavenging activity ranged from 10.7% to 91%. Several nitrones displayed higher activity than the reference antioxidant Trolox (73%). Among the most active compounds, nitrones **11ab** and **11bg** exhibited particularly strong competition with DMSO for ^•^OH radicals generated in this assay system, affording scavenging activities of 91% and 89%, respectively.

The antioxidant activity of the synthesized nitrones was further evaluated using the ABTS^•+^ decolorization assay (Table 1) [48]. The results revealed significant antioxidant potency for nitrone **11bd**, **11bf** and **11bg**, which exhibited radical scavenging activities of 85%, 87.8% and 95%, respectively. These values were comparable to, or in the case of **11bg** even slightly exceeded, the activity of the reference compound Trolox. In contrast, the remaining compounds displayed only moderate antioxidant activity, with scavenging efficiencies ranging from 35% to 51%. Lipophilicity did not show any influence on the scavenging results. The results from the ABTS radical cation decolorization assay support the role of the carbazole moiety in the observed antioxidant activity, compared with that of the previously reported nitrones **5** and **6** (Figure 1). While N-methylated carbazole nitrones **11aa** (51%) and **11ba** (48%) appeared to be significantly active, triazole-derived nitrones **5** (5.3–34.3%) [23] and nucleobase-derived nitrones **6** (5–19%) [25] presented only limited activity. Similarly, the quinoline-derived nitrone QN23 **3** (7%) was less active [49] than N-*tert*-butyl nitrones **11ab** (50%) and **11bb** (49%) investigated in these studies, further supporting the role of the carbazole unit in enhancing antioxidant potential.

Finally, we tested the anti-inflammatory activity of the new compounds through their ability to inhibit soybean lipoxygenase (Table 1). Lipoxygenases catalyze the highly regio- and stereospecific oxygenation of *Z*,*Z*-1,4-diene units in polyunsaturated fatty acids and their derivatives to produce chiral conjugated diene hydroperoxides. LOXs are expressed in immune, epithelial, and tumor cells, playing an important role in various pathophysiological disorders. Upon appropriate stimulation, neutrophils cleave arachidonic acid (AA) from membrane phospholipids, leading to the production of leukotrienes via the LOX pathway. Among them, leukotriene B4 (LTB4) acts as a key inflammatory mediator, contributing to the pathogenesis of neutrophil-mediated inflammatory diseases such as cardiovascular disease, stroke, and cancer.

Soybean lipoxygenases (LOXs) are used in vitro assays to investigate the inhibitory activities of new bioactive compounds. The ability of the synthesized compounds to inhibit soybean LOX by the UV absorbance-based enzyme protocol was evaluated [47]. Among all the tested compounds, nitrone **11be**, 2-substituted carbazole bearing (*S*)-1-phenylethyl group, appeared to be the most potent inhibitor (0.0225 µM), 20-fold more potent than NDGA (0.45 µM) used as a standard in this test and is approximately 293-fold lower than that of the opposite enantiomer **11bd**. It is interesting that between (*R*)-enantiomer **11bd** and (*S*)-**11be**, the second is the most potent. Biological systems rely significantly on molecular shape. Since enzymes are themselves chiral, they usually only recognize and interact with one specific enantiomer. Several other nitrones showed high IC_50_ inhibition values (Table 1, IC_50_ = 6.6–26 µM), including 4-substituted (**11aa**, **11ab**, **11ad** and **11ag**) as well as 2-substituted carbazole derivatives (**11bd**, and **11bg**), however being 15- to 58-fold less potent than NDGA. Although lipophilicity is considered an important property for LOX inhibition, the results undertaken herein do not support this idea. On the contrary, stereospecificity seems to be a very important property. Again, despite the apparent role of an optimized substituent at the nitrogen atom of the nitrone function, the carbazole moiety may contribute to the inhibitory activity of these compounds. However, its contribution cannot be considered independently of the nature of the N-substituent and the stereochemistry of the molecules. Thus, the N-methylated carbazole derivative **11aa** (IC_50_ = 25 µM) was found to be more active than the previously reported N-methylated triazole-derived nitrones **5** (IC_50_ > 37.5 µM) [23] as well as nucleobase-derived nitrones **6** (IC_50_ = 28-79 µM) [25]. These comparisons suggest that the carbazole moiety may favor LOX inhibition, although its contribution should be interpreted in the context of the other structural features that influence activity.

## 3. Materials and Methods

### 3.1. Chemistry—General Information

^1^H and ^13^C NMR spectra were taken in CDCl_3_, CD_3_OD or DMSO-*d*_6_ on the Bruker Avance III spectrometers (Bruker Instruments, Karlsruhe, Germany) (600 MHz) with TMS as the internal standard at 600 and 151 MHz, respectively. IR spectra were measured on a Bruker Alpha-T FT-IR spectrometer (Bruker Optik GmbH, Ettlingen, Germany). Melting points were determined on a Boetius apparatus and are uncorrected. Elemental analyses were performed by the Microanalytical Laboratory of this Faculty on Perkin-Elmer PE 2400 CHNS analyzer (Perkin Elmer Corp., Norwalk CT, USA), and their results were found to be in good agreement (±0.3%) with the calculated values. Polarimetric measurements were conducted with an Optical Activity PolAr 3001 apparatus (Optical Activity Ltd., Ramsey, United Kingdom). The following adsorbents were used: column chromatography, Merck silica gel 60 (70–230 mesh); analytical TLC, Merck TLC plastic sheets silica gel 60 F_254_.

### 3.2. General Procedure for the Synthesis of Epoxides ***13a*** and ***13b***

To a solution of the respective hydroxycarbazole **12** (1.0 mmol) in DMSO (0.6 mL), 1 M NaOH (1.14 mmol) was added followed by epibromohydrin (1.11 mmol). The reaction mixture was stirred at room temperature for 24 h, diluted with water (15 mL) and extracted with ethyl acetate (2 × 15 mL). The combined organic layers were dried over Na_2_SO_4_, filtered and concentrated to give crude product **13**.

4-(Oxiran-2-ylmethoxy)-9*H*-carbazole (**13a**). According to the general procedure from 4-hydroxycarbazole **12a** (0.300 g, 1.64 mmol), pure product **13a** (0.345 g, 88%) was obtained as a white amorphous solid after purification on a silica gel column using chloroform as eluent: m.p. 134–136 °C (m.p. 135–138 °C) [41]; ^1^H NMR (600 MHz, CDCl_3_) δ 8.34 (d, *J* = 7.8 Hz, 1H, H_arom_), 8.06 (s, 1H, N**H**), 7.42–7.38 (m, 2H, H_arom_), 7.32 (t, *J* = 8.1 Hz, 1H, H_arom_), 7.27–7.23 (m, 1H, H_arom_), 7.07 (d, *J* = 8.1 Hz, 1H, H_arom_), 6.67 (d, *J* = 8.1 Hz, 1H, H_arom_), 4.47 (dd, *J* = 10.8 Hz, *J =* 3.3 Hz, 1H, OCH_2_), 4.28 (dd, *J* = 10.8 Hz, *J* = 5.1 Hz, 1H, OCH_2_), 3.58–3.54 (m, 1H, OC**H**CH_2_), 3.00 (t, *J* = 4.8 Hz, 1H, CHC**H_2_**O), 2.90 (dd, *J* = 4.8 Hz, *J* = 3.0 Hz, 1H, CHC**H_2_**O).

2-(Oxiran-2-ylmethoxy)-9*H*-carbazole (**13b**). According to the general procedure from 2-hydroxycarbazole **12b** (0.200 g, 1.09 mmol), pure product **13b** (0.135 g, 52%) was obtained as beige powder after purification on silica gel column using chloroform as eluent: m.p. 198–201 °C; IR (KBr, cm^−1^) ν_max_ 3388, 3053, 3000, 2925, 1628, 1609, 1462, 1309, 1180, 1033; ^1^H NMR (600 MHz, DMSO-*d_6_*) δ 11.09 (s, 1H, N**H**), 7.99–7.95 (m, 2H, H_arom_), 7.41 (d, *J* = 8.1 Hz, 1H, H_arom_), 7.28 (t, *J* = 7.2 Hz, 1H, H_arom_), 7.10 (t, *J* = 7.2 Hz, 1H, H_arom_), 7.00–6.98 (m, 1H, H_arom_), 6.81–6.78 (m, 1H, H_arom_), 4.42–4.38 (m, 1H, OCH_2_), 3.93–3.89 (m, 1H, OCH_2_), 2.88–2.85 (m, 1H, CHC**H_2_**O), 2.77–2.74 (m, 1H, CHC**H_2_**O), 2.55–2.53 (m, 1H, OC**H**CH_2_).

### 3.3. General Procedure for the Preparation of Diol ***14***

To a suspension of carbazole derivative **13** (0.135 g, 0.564 mmol) in H_2_O (10 mL), a catalytic amount of concentrated sulfuric acid (10 drops) was added dropwise. The mixture was heated at 100 °C for 24 h. After cooling to room temperature, the mixture was neutralized with solid NaHCO_3_ and extracted with ethyl acetate (3 × 50 mL). The combined organic layers were dried over Na_2_SO_4_, filtered, and concentrated in vacuo to afford pure diol **14**.

3-[(9*H*-Carbazol-4-yl)oxy]propane-1,2-diol (**14a**). According to the general procedure from epoxycarbazole **13a** (0.400 g, 1.55 mmol), pure product **14a** (0.337 g, 78%) was obtained as a white amorphous solid after purification on a silica gel column (CHCl_3_:MeOH, 100:1, *v*/*v*): m.p. 95–97 °C; IR (KBr, cm^−1^) ν_max_ 3394, 3051, 2933, 2876, 1626, 1604, 1454, 1334, 1262, 1214, 1099; ^1^H NMR (600 MHz, DMSO-*d*_6_) δ 11.21 (s, 1H, N**H**), 8.22 (d, *J* = 7.8 Hz, 1H, H_arom_), 7.45 (d, *J* = 7.5 Hz, 1H, H_arom_), 7.34 (t, *J* = 7.5 Hz, 1H, H_arom_), 7.29 (t, *J* = 7.5 Hz, 1H, H_arom_), 7.16–7.12 (m, 1H, H_arom_), 7.07 (d, *J* = 7.8 Hz, 1H, H_arom_), 6.68 (d, *J* = 7.8 Hz, 1H, H_arom_), 5.08–5.04 (m, 1H, OC**H_2_**CH), 4.75–4.71 (m, 1H, OC**H_2_**CH), 4.24–4.19 (m, 1H, CHO**H**), 4.16–4.11 (m, 1H, CH_2_O**H**), 4.04–3.98 (m, 1H, CH_2_C**H**CH_2_), 3.68–3.58 (m, 2H, C**H_2_**OH); ^13^C NMR (151 MHz, DMSO-*d*_6_) δ 155.55, 141.59, 139.40, 126.94, 124.97, 122.97, 122.27, 119.01, 112.12, 110.79, 104.25, 100.90, 70.72, 69.84, 63.44. Anal. Calcd. for C_15_H_15_NO_3_: C, 70,02; H, 5,88; N, 5.44. Found: C, 69,90; H, 5,78; N, 5,45.

3-[(9*H*-Carbazol-2-yl)oxy]propane-1,2-diol (**14b**). According to the general procedure from epoxycarbazole **13b** (0.135 g, 0.564 mmol), pure product **14b** (0.102 g, 70%) was obtained as a white amorphous solid after purification on a silica gel column (CHCl_3_:MeOH, 50:1, *v*/*v*): m.p. 241–243 °C; IR (KBr, cm^−1^) ν_max_ 3397, 3047, 2947, 2881, 1631, 1610, 1459, 1332, 1311, 1294, 1228, 1175, 1033; ^1^H NMR (600 MHz, CD_3_OD) δ 7.92 (d, *J* = 7.8 Hz, 1H, H_arom_), 7.89 (d, *J* = 8.5 Hz, 1H, H_arom_), 7.37 (d, *J* = 8.0 Hz, 1H, H_arom_), 7.29–7.24 (m, 1H, H_arom_), 7.10 (t, *J* = 7.4 Hz, 1H, H_arom_), 7.00 (d, *J* = 2.2 Hz, 1H, H_arom_), 6.82 (dd, *J* = 8.5 Hz, *J* = 2.2 Hz, 1H, H_arom_), 4.15 (dd, *J* = 9.4 Hz, *J* = 4.4 Hz, 1H, OC**H_2_**CH), 4.06 (dd, *J* = 9.4 Hz, *J* = 5.9 Hz, 1H, OC**H_2_**CH), 4.06–4.02 (m, 1H, CH_2_C**H**CH_2_), 3.74 (dd, *J* = 11.3 Hz, *J* = 5.1 Hz, 1H, C**H_2_**OH), 3.69 (dd, *J* = 11.3 Hz, *J* = 5.6 Hz, 1H, **CH_2_**OH); ^13^C NMR (151 MHz, CD_3_OD) δ 158.25, 141.25, 140.19, 123.84, 122.96, 120.16, 118.63, 118.34, 116.52, 109.95, 107.86, 95.04, 70.57, 69.41, 62.94. Anal. Calcd. for C_15_H_15_NO_3_: C, 70.02; H, 5.88; N, 5.44. Found: C, 69.90; H, 5.82; N, 5.42.

### 3.4. General Procedure for the Preparation of Aldehyde ***15***

A mixture of diol **14** (1.00 mmol), NaIO_4_ (1.20 mmol), water (2 mL) and ethyl acetate (10 mL) was stirred at room temperature for 24 h. The organic layer was separated and the water phase was extracted with ethyl acetate (3 × 5 mL). The organic extracts were collected, dried (Na_2_SO_4_) and evaporated in vacuo to afford a crude aldehyde **15**, which was immediately used in the next step without purification.

Compound **15a**. From diol **14a** (0.415 g, 1,61 mmol), crude aldehyde **15a** (0,347 g, 96%) was obtained as a 17:83 mixture of aldehyde and respective hydrate, which was isolated as a white powder: ^1^H NMR (600 MHz, DMSO-*d*_6_) δ 11.29 (s, 0.17 × 1H, N**H**), 11.21 (s, 0.83 × 1H, N**H**), 9.86 (s, 0.17 × 1H, C**H**O), 8.31–8.27 (m, 0.17 × 1H, H_arom_), 8.26–8.22 (m, 0.83 × 1H, H_arom_), 7.47 (d, *J* = 8.4 Hz, 0.17 × 1H, H_arom_), 7.44 (d, *J* = 8.4 Hz, 0.83 × 1H, H_arom_), 7.36 (t, *J* = 7.5 Hz, 0.17 × 1H, H_arom_), 7.33 (t, *J* = 7.5 Hz, 0.83 × 1H, H_arom_), 7.28 (t, *J* = 7.8 Hz, 1H, H_arom_), 7.17 (t, *J* = 7.5 Hz, 0.17 × 1H, H_arom_), 7.14–7.11 (m, 1H, H_arom_), 7.08–7.05 (m, 0.83 × 1H, H_arom_), 6.65 (d, *J* = 7.8 Hz, 0.83 × 1H, H_arom_), 6.62 (d, *J* = 7.8 Hz, 0.17 × 1H, H_arom_), 6.18–6.14 (m, 0.83 × 2H, 2 × O**H**), 5.32–5.27 (m, 0.83 × 1H, -CH(OH)_2_), 5.07 (s, 0.17 × 2H, OC**H_2_**), 4.05–4.01 (m, 0.83 × 2H, OC**H_2_**).

Compound **15b**. From diol **14b** (0.360 g, 1,40 mmol), crude aldehyde **15b** (0,280 g, 89%) was obtained as a 38:62 mixture of aldehyde and respective hydrate, which was isolated as a white powder: ^1^H NMR (600 MHz, DMSO-*d*_6_) δ 11.10 (s, 0.38 × 1H, N**H**), 11.05 (s, 0.62 × 1H, N**H**), 9.75 (s, 0.38 × 1H, C**H**O), 8.00–7.93 (m, 2H, H_arom_), 7.43–7.39 (m, 1H, H_arom_), 7.31–7.25 (m, 1H, H_arom_), 7.12–7.08 (m, 1H, H_arom_), 6.95 (d, *J* = 2.1 Hz, 1H, H_arom_), 6.80 (dd, *J* = 8.7 Hz, *J* = 2.4 Hz, 0.38 × 1H, H_arom_), 6.75 (dd, *J* = 8.4 Hz, *J* = 2.1 Hz, 0.62 × 1H, H_arom_), 6.09 (d, *J* = 6.6 Hz, 0.62 × 2H, 2 × O**H**), 5.14–5.10 (m, 0.62 × 1H, -C**H**(OH)_2_), 4.92 (s, 0.38 × 2H, OC**H_2_**), 3.87 (d, *J* = 5.1 Hz, 0.62 × 2H, OC**H_2_**CH).

### 3.5. General Procedure for the Preparation of Nitrones ***11aa***–***ag*** and ***11ba***–***bg***

To a solution of crude aldehyde **15** [obtained from respective diol **14** (1.00 mmol)] in ethanol (10 mL), Na_2_CO_3_ (1.30 mmol) and the respective salt of hydroxylamine (1.10 mmol) were added. The mixture was stirred at room temperature for 1 to 5 h. Then, a saturated solution of NaHCO_3_ (10 mL) was added, and the reaction mixture was extracted with CH_2_Cl_2_ (3 × 15 mL). The combined organic extracts were dried over Na_2_SO_4_ and evaporated in vacuo to afford the crude nitrones **11**, which was purified by column chromatography on silica gel or washed with an ethyl acetate and hexane mixture (1:1 *v*/*v*).

2-[(9*H*-Carbazol-4-yl)oxy]-*N*-methylethan-1-imine oxide (**11aa**). According to the general procedure from the crude aldehyde **15a** (0.325 g, 1.44 mmol) [obtained directly from diol **14a** (0.300 g, 1.17 mmol)], *N*-methylhydroxylamine hydrochloride (0.132 g, 1.58 mmol), the pure nitrone **11aa** (0.194 g, 65% calculated for two steps, starting from diol **14a**) was obtained as a white solid after 1 h of the reaction and subsequent purification by column chromatography on silica gel (CH_2_Cl_2_:MeOH, 100:1, *v*/*v*): m.p. 96–98 °C; IR (KBr, cm^−1^) ν_max_: 3384, 3297, 2991, 2933, 2815, 1624, 1602, 1586, 1452, 1438, 1348, 1331, 1259, 1227, 1213; ^1^H NMR (600 MHz, DMSO-*d*_6_) δ 11.28 (s, 1H, N**H**), 8.19 (d, *J* = 7.8 Hz, 1H, H_arom_), 7.62 (t, *J* = 4.8 Hz, 1H, CH=N), 7.46 (d, *J* = 8.4 Hz, 1H, H_arom_), 7.36 (t, *J* = 7.2 Hz, 1H, H_arom_), 7.31 (t, *J* = 7.8 Hz, 1H, H_arom_), 7.15 (t, *J* = 7.8 Hz, 1H, H_arom_), 7.12 (d, *J* = 8.0 Hz, 1H, H_arom_), 6.69 (d, *J* = 7.8 Hz, 1H, H_arom_), 5.08 (dd, *J* = 4.8 Hz, *J* = 1.6 Hz, 2H, OC**H**_2_), 3.70 (s, 3H, NC**H_3_**); ^13^C NMR (151 MHz, DMSO-*d_6_*) δ 154.44, 141.69, 139.47, 135.91, 126.99, 125.20, 122.90, 121.94, 119.11, 111.95, 110.96, 104.99, 100.91, 63.37, 52.39. Anal. Calcd. for C_15_H_14_N_2_O_2_: C, 70.85; H, 5.55; N, 11.02. Found: C, 70.83; H, 5,37; N, 11.09.

2-[(9*H*-Carbazol-4-yl)oxy]-*N*-(*tert*-butyl)ethan-1-imine oxide (**11ab**). According to the general procedure from the crude aldehyde **15a** (0.400 g, 1.76 mmol) [obtained directly from diol **14a** (0.350 g, 1.36 mmol)], *N*-*tert*-butylhydroxylamine hydrochloride (0.243 g, 1.94 mmol), the pure nitrone **11ab** (0.160 g, 40% calculated for two steps, starting from diol **14a**) was obtained as a white solid after 1 h of the reaction and subsequent purification by column chromatography on silica gel (CHCl_3_): m.p. 150–152 °C; IR (KBr, cm^−1^) ν_max_: 3408, 3149, 3089, 2990, 2972, 2852, 1606, 1584, 1505, 1444, 1336, 1218, 1136; ^1^H NMR (600 MHz, CDCl_3_) δ 8.36 (s, 1H, N**H**), 8.30 (d, *J* = 7.8 Hz, 1H, H_arom_), 7.43–7.38 (m, 2H, H_arom_), 7.33–7.29 (m, 1H, H_arom_, 1H, C**H**=N), 7.27–7.24 (m, 1H, H_arom_), 7.08 (d, *J* = 8.0 Hz, 1H, H_arom_), 6.64 (d, *J* = 7.9 Hz, 1H, H_arom_), 5.28 (d, *J* = 4.1 Hz, 2H, OC**H**_2_), 1.58 (s, 9H, C(C**H_3_**)_3_); ^13^C NMR (151 MHz, CDCl_3_) δ 154.42, 141.16, 138.87, 132.39, 126.76, 125.14, 122.92, 122.41, 119.59, 112.62, 110.18, 104.33, 101.12, 69.70, 64.66, 27.91. Anal. Calcd. for C_18_H_20_N_2_O_2_: C, 72.95; H, 6.80; N, 9.45. Found: C, 72.81; H, 6.59; N, 9.54.

2-[(9*H*-Carbazol-4-yl)oxy]-*N*-benzylethan-1-imine oxide (**11ac**). According to the general procedure from the crude aldehyde **15a** (0.330 g, 1.47 mmol) [obtained directly from diol **14a** (0.340 g, 1.32 mmol)], *N*-benzylhydroxylamine hydrochloride (0.258 g, 1.62 mmol), the pure nitrone **11ac** (0.132 g, 30% calculated for two steps, starting from diol **14a**) was obtained as a white solid after 1 h of the reaction and subsequent purification by column chromatography on silica gel (hexane:ethyl acetate, 5:1, *v*/*v*): m.p. 173–175 °C; IR (KBr, cm^−1^) ν_max_ 3418, 3097, 2986, 2889, 1630, 1606, 1511, 1457, 1347, 1268, 1212, 1162, 1111; ^1^H NMR (600 MHz, DMSO-*d_6_*) δ 11.29 (s, 1H, N**H**), 8.19 (d, *J* = 7.8 Hz, 1H, H_arom_), 7.86 (t, *J* = 4.7 Hz, 1H, -CH=N), 7.51–7.45 (m, 3H, H_arom_), 7.43–7.33 (m, 4H, H_arom_), 7.28 (t, *J* = 8.0 Hz, 1H, H_arom_), 7.16 (t, *J* = 7.5 Hz, 1H, H_arom_), 7.12 (d, *J* = 8.0 Hz, 1H, H_arom_), 6.65 (d, *J* = 7.9 Hz, 1H, H_arom_), 5.10 (d, *J* = 4.7 Hz, 2H, OC**H**_2_), 5.04 (s, 2H, C**H**_2_Ph); ^13^C NMR (151 MHz, DMSO-*d_6_*) δ 154.41, 141.67, 139.47, 135.44, 134.67, 129.45, 128.84, 128.79, 126.95, 125.21, 122.88, 121.93, 119.11, 111.94, 110.97, 105.00, 100.93, 68.36, 63.27. Anal. Calcd. for C_21_H_18_N_2_O_2_: C, 76.34; H, 5.49; N, 8.48. Found: C, 76.28; H, 5.37; N, 8.37.

(*R*)-2-[(9*H*-Carbazol-4-yl)oxy]-*N*-(1-phenylethyl)ethan-1-imine oxide (**11ad**). According to the general procedure from the crude aldehyde **15a** (0.326 g, 1.45 mmol) [obtained directly from diol **14a** (0.385 g, 1.50 mmol)], (*R*)-(+)-(1-phenylethyl)hydroxylamine oxalate (0.362 g, 1.59 mmol), the pure nitrone **11ad** (0.195 g, 38% calculated for two steps, starting from diol **14a**) was obtained as green powder after 3 h of the reaction and subsequent purification by column chromatography on silica gel (hexane:ethyl acetate, 5:1, *v*/*v*): m.p. 73–76 °C; ${\left[\alpha \right]}_{D}^{20}$ +42.73 (*c* 0.94, CHCl_3_); IR (KBr, cm^−1^) ν_max_ 3406, 3229, 3059, 2932, 1606, 1584, 1508, 1454, 1347, 1260, 1212, 1150; ^1^H NMR (600 MHz, CDCl_3_) δ 8.23 (d, *J* = 7.8 Hz, 1H, H_arom_), 8.18 (s, 1H, N**H**), 7.50–7.48 (m, 2H, H_arom_), 7.42–7.35 (m, 5H, H_arom_), 7.28 (d, *J* = 8.0 Hz, 1H, H_arom_), 7.25–7.20 (m, 2H, H_arom_, C**H**=N), 7.06 (d, *J* = 8.0 Hz, 1H, H_arom_), 6.59 (d, *J* = 8.0 Hz, 1H, H_arom_), 5.27 (dAB, ^2^*J_AB_* = 16.9 Hz, ^3^*J* = 4.4 Hz, 1H, OC**H**_2_), 5.21 (dAB, ^2^*J_AB_* = 16.9 Hz, ^3^*J* = 4.4 Hz, 1H, OC**H**_2_), 5.11 (q, *J* = 6.9 Hz, 1H, C**H**CH_3_), 1.87 (d, *J* = 6.9 Hz, 3H, CHC**H_3_**); ^13^C NMR (151 MHz, CDCl_3_) δ 154.27, 141.06, 138.80, 137.80, 134.59, 128.94, 128.84, 127.35, 126.74, 125.15, 122.93, 122.37, 119.63, 112.63, 110.12, 104.33, 101.22, 73.66, 63.69, 18.96. Anal. Calcd. for C_22_H_20_N_2_O_2_: C, 76.72; H, 5.85; N, 8.13. Found: C, 76.55; H, 5.73; N, 8.09.

(*S*)-2-[(9*H*-Carbazol-4-yl)oxy]-*N*-(1-phenylethyl)ethan-1-imine oxide (**11ae**). According to the general procedure from the crude aldehyde **15a** (0.340 g, 1.51 mmol) [obtained directly from diol **14a** (0.350 g, 1.36 mmol)], (*S*)-(−)-(1-phenylethyl)hydroxylamine oxalate (0.377 g, 1.66 mmol), the pure nitrone **11ae** (0.129 g, 28% calculated for two steps, starting from diol **14a**) was obtained as green powder after 3 h of the reaction and subsequent purification by column chromatography on silica gel (hexane:ethyl acetate, 5:1, *v*/*v*): m.p. 72–75 °C; ${\left[\alpha \right]}_{D}^{20}$ −41.69 (*c* 0.98, CHCl_3_); IR and NMR spectra of (*S*)-**11ae** were identical with its enantiomer (*R*)-**1ad**. Anal. Calcd. for C_22_H_20_N_2_O_2_: C, 76.72; H, 5.85; N, 8.13. Found: C, 76.54; H, 5.74; N, 8.08.

(*R*)-2-[(9*H*-Carbazol-4-yl)oxy]-*N*-[1-(naphthalen-1-yl)ethyl]ethan-1-imine oxide (**11af**). According to the general procedure from the crude aldehyde **15a** (0.310 g, 1.38 mmol) [obtained directly from diol **14a** (0.330 g, 1.28 mmol)], (*R*)-(+)-(1-(1-naphthyl)ethyl)hydroxylamine oxalate (0.421 g, 1.52 mmol), the pure nitrone **1af** (0.116 g, 23% calculated for two steps, starting from diol **14a**) was obtained as a brown powder after 5 h of the reaction and subsequent purification by column chromatography on silica gel (hexane:ethyl acetate, 5:1, *v*/*v*): m.p. 90–93 °C; ${\left[\alpha \right]}_{D}^{20}$ −57.77 (*c* 0.77, CHCl_3_); IR (KBr, cm^−1^) ν_max_ 3406, 3230, 3053, 2984, 2937, 1606, 1584, 1508, 1454, 1441, 1347, 1260, 1211; ^1^H NMR (600 MHz, CDCl_3_) δ 8.17 (s, 1H, N**H**), 8.10 (d, *J* = 8.5 Hz, 1H, H_arom_), 8.04 (d, *J* = 6.7 Hz, 1H, H_arom_), 7.90 (d, *J* = 8.2 Hz, 1H, H_arom_), 7.87 (d, *J* = 8.2 Hz, 1H, H_arom_), 7.74 (d, *J* = 7.2 Hz, 1H, H_arom_), 7.59 (t, *J* = 8.4 Hz, 1H, H_arom_), 7.54 (t, *J* = 6.8 Hz, 1H, H_arom_), 7.50 (t, *J* = 7.2 Hz, 1H, H_arom_), 7.35–7.33 (m, 2H, H_arom_), 7.18–7.12 (m, 2H, H_arom_), 6.98 (d, *J* = 8.1 Hz, 1H, H_arom_), 6.91 (t, *J* = 4.4 Hz, 1H, C**H**=N), 6.43 (d, *J* = 8.0 Hz, 1H, H_arom_), 5.87 (q, *J* = 6.9 Hz, 1H, C**H**CH_3_), 5.21 (dAB, ^2^*J_AB_* = 17.0 Hz, ^3^*J* = 4.5 Hz, 1H, OC**H**_2_), 5.17 (dAB, ^2^*J_AB_* = 17.0 Hz, ^3^*J* = 4.4 Hz, 1H, OC**H**_2_), 2.06 (d, *J* = 6.9 Hz, 3H, CHC**H_3_**); ^13^C NMR (151 MHz, CDCl_3_) δ 154.14, 140.98, 138.73, 135.36, 133.93, 132.63, 131.29, 130.00, 129.18, 127.33, 126.62, 126.18, 125.32, 125.07, 125.01, 122.83, 122.46, 122.24, 119.56, 112.49, 110.06, 104.25, 101.13, 70.55, 63.78, 19.09. Anal. Calcd. for C_26_H_22_N_2_O_2_ × 1.25H_2_O: C, 74.89; H, 5.92; N, 6.72. Found: C, 75.15; H, 5.74; N, 7.02.

(*S*)-2-[(9*H*-Carbazol-4-yl)oxy]-*N*-[1-(naphthalen-1-yl)ethyl]ethan-1-imine oxide (**11ag**). According to the general procedure from the crude aldehyde **15a** (0.363 g, 1.61 mmol) obtained directly from diol **14a** (0.421 g, 1.64 mmol)], (*S*)-(−)-(1-(1-naphthyl)ethyl)hydroxylamine oxalate (0.491 g, 1.77 mmol), the pure nitrone **11ag** (0.165 g, 26% calculated for two steps, starting from diol **14a**) was obtained as a brown powder after 5 h of the reaction and subsequent purification by column chromatography on silica gel (hexane:ethyl acetate, 5:1, *v*/*v*): m.p. 89–92 °C; ${\left[\alpha \right]}_{D}^{20}$ +62.54 (*c* 0.91, CHCl_3_); IR and NMR spectra of (*S*)-**11ag** were identical with its enantiomer (*R*)-**1af**. Anal. Calcd. for C_26_H_22_N_2_O_2_ × 1.25H_2_O: C, 74.89; H, 5.92; N, 6.72. Found: C, 75.19; H, 5.64; N, 6.99.

2-[(9*H*-Carbazol-2-yl)oxy]-*N*-methylethan-1-imine oxide (**11ba**). According to the general procedure from the crude aldehyde **15b** (0.330 g, 1.47 mmol) obtained directly from diol **14b** (0.380 g, 1.48 mmol)], *N*-methylhydroxylamine hydrochloride (0.135 g, 1.62 mmol), the pure nitrone **11ba** (0.276 g, 73% calculated for two steps, starting from diol **14b**) was obtained as beige powder after 3 h of the reaction and subsequent washing of crude product with hexane:ethyl acetate mixture (1:1, *v*/*v*) (3 × 16 mL): m.p. 196–199 °C; IR (KBr, cm^−1^) ν_max_: 3402, 3169, 3096, 3066, 1639, 1609, 1464, 1441, 1403, 1328, 1292, 1235, 1174; ^1^H NMR (600 MHz, DMSO-*d_6_*) δ 11.12 (s, 1H, N**H**), 8.00–7.97 (m, 1H, H_arom_, 1H, C**H**=N), 7.43–7.41 (m, 2H, H_arom_), 7.29 (t, *J* = 7.6 Hz, 1H, H_arom_), 7.11 (t, *J* = 7.4 Hz, 1H, H_arom_), 6.98 (d, *J* = 2.3 Hz, 1H, H_arom_), 6.78 (dd, *J* = 8.5 Hz, *J* = 2.3 Hz, 1H, H_arom_), 4.92 (d, *J* = 4.7 Hz, 2H, OC**H**_2_), 3.66 (s, 3H, NC**H_3_**); ^13^C NMR (151 MHz, DMSO-*d_6_*) δ 157.27, 141.45, 140.28, 135.79, 124.78, 122.99, 121.54, 119.81, 119.07, 117.19, 111.13, 108.31, 95.71, 63.29, 52.42. Anal. Calcd. for C_15_H_14_N_2_O_2_: C, 70.85; H, 5.55; N, 11.02. Found: C, 70.84; H, 5,33; N, 11.09.

2-[(9*H*-Carbazol-2-yl)oxy]-*N*-(*tert*-butyl)ethan-1-imine oxide (**11bb**). According to the general procedure from the crude aldehyde **15b** (0.166 g, 0.74 mmol) obtained directly from diol **14b** (0.261 g, 1.01 mmol)], *N*-*tert*-butylhydroxylamine hydrochloride (0.102 g, 0.81 mmol), the pure nitrone **11bb** (0.071 g, 24% calculated for two steps, starting from diol **14b**) was obtained as green powder after 1 h of the reaction and subsequent purification by column chromatography on silica gel (CH_2_Cl_2_:MeOH, 100:1 *v*/*v*): m.p. 189–192 °C; IR (KBr, cm^−1^) ν_max_ 3456, 3251, 3101, 2973, 2930, 2893, 1633, 1606, 1464, 1305, 1291, 1242, 1186; ^1^H NMR (600 MHz, CDCl_3_) δ 8.30 (s, 1H, N**H**), 7.98 (d, *J* = 7.8 Hz, 1H, H_arom_), 7.94 (d, *J* = 8.5 Hz, 1H, H_arom_), 7.39 (d, *J* = 8.0 Hz, 1H, H_arom_), 7.37–7.32 (m, 1H, H_arom_), 7.24–7.17 (m, 1H, H_arom_, 1H, C**H**=N), 6.92 (d, *J* = 2.3 Hz, 1H, H_arom_), 6.85 (dd, *J* = 8.5 Hz, *J* = 2.3 Hz, 1H, H_arom_), 5.08 (d, *J* = 4.2 Hz, 2H, OC**H**_2_), 1.55 (s, 9H, N(C**H_3_**)_3_); ^13^C NMR (151 MHz, CDCl_3_) δ 157.21, 140.83, 139.68, 132.28, 124.74, 123.35, 121.23, 119.55, 119.54, 117.77, 110.46, 108.52, 95.19, 69.72, 64.69, 27.88. Anal. Calcd. for C_18_H_20_N_2_O_2_: C, 72.95; H, 6.80; N, 9.45. Found: C, 72.86; H, 6.50; N, 9.59.

2-[(9*H*-Carbazol-2-yl)oxy]-*N*-benzylethan-1-imine oxide (**11bc**). According to the general procedure from the crude aldehyde **15b** (0.246 g, 1.09 mmol) obtained directly from diol **14b** (0.355 g, 1.38 mmol)], *N*-benzylhydroxylamine hydrochloride (0.191 g, 1.20 mmol), the pure nitrone **11bc** (0.116 g, 25% calculated for two steps, starting from diol **14b**) was obtained as slightly brownish powder after 3 h of the reaction and subsequent washing of crude product with hexane:ethyl acetate (1:1, *v*/*v*) (3 × 16 mL): m.p. 204–207 °C; IR (KBr, cm^−1^) ν_max_: 3394, 3076, 3064, 2979, 2939, 1639, 1612, 1460, 1443, 1343, 1294, 1235, 1174; ^1^H NMR (600 MHz, DMSO-*d_6_*) δ 11.13 (s, 1H, N**H**), 8.00–7.96 (m, 2H, H_arom_), 7.63 (t, *J* = 4.8 Hz, 1H, CH=N), 7.46–7.41 (m, 3H, H_arom_), 7.38–7.35 (m, 3H, H_arom_), 7.29 (t, *J* = 7.6 Hz, 1H, H_arom_), 7.11 (t, *J* = 8.0 Hz, 1H, H_arom_), 6.99 (d, *J* = 2.3 Hz, 1H, H_arom_), 6.78 (dd, *J* = 8.5 Hz, *J* = 2.3 Hz, 1H, H_arom_), 4.99 (s, 2H, C**H**_2_Ph), 4.94 (d, *J* = 4.7 Hz, 2H, OC**H**_2_); ^13^C NMR (151 MHz, DMSO-*d_6_*) δ 157.22, 141.44, 140.29, 135.42, 134.53, 129.47, 128.87, 128.80, 124.80, 122.98, 121.53, 119.82, 119.08, 117.24, 111.14, 108.29, 95.81, 68.37, 63.38. Anal. Calcd. for C_21_H_18_N_2_O_2_: C, 76.34; H, 5.49; N, 8.48. Found: C, 76.28; H, 5.37; N, 8.39.

(*R*)-2-[(9*H*-Carbazol-2-yl)oxy]-*N*-(1-phenylethyl)ethan-1-imine oxide (**11bd**). According to the general procedure from the crude aldehyde **15b** (0.260 g, 1.15 mmol) obtained directly from diol **14b** (0.381 g, 1.48 mmol)], (*R*)-(+)-(1-phenylethyl)hydroxylamine oxalate (0.288 g, 1.27 mmol), the pure nitrone **11bd** (0.138 g, 27% calculated for two steps, starting from diol **14b**) was obtained as slightly brownish powder after 5 h of the reaction and subsequent washing with hexane:ethyl acetate (1:1 *v*/*v*) (3 × 16 mL): m.p. 193–195 °C; ${\left[\alpha \right]}_{D}^{20}$ +5.41 (*c* 0.73, DMSO); IR (KBr, cm^−1^) ν_max_ 3404, 3245, 3080, 2918, 1636, 1608, 1463, 1453, 1349, 1291, 1234, 1169; ^1^H NMR (600 MHz, DMSO-*d_6_*) δ 11.11 (s, 1H, N**H**), 7.98 (d, *J* = 7.8 Hz, 1H, H_arom_), 7.96 (d, *J* = 8.5 Hz, 1H, H_arom_), 7.64 (t, *J* = 4.7 Hz, 1H, C**H**=N), 7.52–7.47 (m, 2H, H_arom_), 7.41 (d, *J* = 8.0 Hz, 1H, H_arom_), 7.37–7.31 (m, 3H, H_arom_), 7.30–7.27 (m, 1H, H_arom_), 7.10 (t, *J* = 7.4 Hz, 1H, H_arom_), 6.96 (d, *J* = 2.3 Hz, 1H, H_arom_), 6.75 (dd, *J* = 8.5 Hz, *J* = 2.3 Hz, 1H, H_arom_), 5.38 (q, *J* = 6.8 Hz, 1H, C**H**CH_3_), 4.95 (dAB,^2^*J_AB_* = 15.9 Hz, ^3^*J* = 4.9 Hz, 1H, OC**H**_2_), 4.89 (dAB, ^2^*J_AB_* = 16.0 Hz, ^3^*J* = 4.6 Hz, 1H, OC**H**_2_), 1.65 (d, *J* = 6.8 Hz, 3H, CHC**H_3_**); ^13^C NMR (151 MHz, DMSO-*d_6_*): δ 157.20, 141.42, 140.28, 139.23, 133.95, 128.74, 128.70, 127.72, 124.80, 122.97, 121.50, 119.80, 119.08, 117.20, 111.13, 108.28, 95.75, 71.73, 63.34, 18.87. Anal. Calcd. for C_22_H_20_N_2_O_2_: C, 76.72; H, 5.85; N, 8.13. Found: C, 76.46; H, 5.74; N, 7.91.

(*S*)-2-[(9*H*-Carbazol-2-yl)oxy]-*N*-(1-phenylethyl)ethan-1-imine oxide (**11be**). According to the general procedure from the crude aldehyde **15b** (0.310 g, 1.38 mmol) obtained directly from diol **14b** (0.351 g, 1.36 mmol)], (*S*)-(−)-(1-phenylethyl)hydroxylamine oxalate (0.345 g, 1.52 mmol), the pure nitrone **11be** (0.136 g, 29% calculated for two steps, starting from diol **14b**) was obtained as slightly brownish powder after 5 h of the reaction and subsequent washing with hexane:ethyl acetate (1:1 *v*/*v*) (3 × 16 mL): m.p. 183–185 °C; ${\left[\alpha \right]}_{D}^{20}$ −5,61 (*c* 0.89, DMSO); IR and NMR spectra of (*S*)-**1be** were identical with its enantiomer (*R*)-**11bd**. Anal. Calcd. for C_22_H_20_N_2_O_2_: C, 76.72; H, 5.85; N, 8.13. Found: C, 76.48; H, 5.74; N, 7.90.

(*R*)-2-[(9*H*-Carbazol-2-yl)oxy]-*N*-[1-(naphthalen-1-yl)ethyl]ethan-1-imine oxide (**11bf**). According to the general procedure from the crude aldehyde **15b** (0.258 g, 1.15 mmol) obtained directly from diol **14b** (0.372 g, 1.45 mmol)], (*R*)-(+)-(1-(1-naphtyl)ethyl)hydroxylamine oxalate (0.349 g, 1.26 mmol), the pure nitrone **11bf** (0.115 g, 20% calculated for two steps, starting from diol **14b**) was obtained as slightly brownish powder after 5 h of the reaction and subsequent purification by column chromatography on silica gel (hexane:ethyl acetate, 5:1, *v*/*v*): m.p. 72–74 °C; ${\left[\alpha \right]}_{D}^{20}$ −21.74 (*c* 0.84, CHCl_3_); IR (KBr, cm^−1^) ν_max_: 3405, 3239, 3051, 2984, 2924, 1631, 1609, 1579, 1491, 1461, 1309, 1290, 1236, 1167; ^1^H NMR (600 MHz, CDCl_3_) δ 8.11–8.05 (m, 1H, H_arom_), 7.94 (d, *J* = 7.8 Hz, 1H, H_arom_), 7.91 (s, 1H, N**H**), 7.89–7.85 (m, 2H, H_arom_), 7.82 (d, *J* = 8.4 Hz, 1H, H_arom_), 7.73 (d, *J* = 7.2 Hz, 1H, H_arom_), 7.53–7.48 (m, 3H, H_arom_), 7.37–7.32 (m, 2H, H_arom_), 7.21–7.18 (m, 1H, H_arom_), 6.80 (t, *J* = 4.5 Hz, 1H, C**H**=N), 6.71–6.64 (m, 2H, H_arom_), 5.85 (q, *J* = 6.9 Hz, 1H, C**H**CH_3_), 5.04 (dAB, ^2^*J_AB_* = 16.9 Hz, ^3^*J* = 4.6 Hz, 1H, OC**H**_2_), 4.97 (dAB, ^2^*J_AB_* = 16.9 Hz, ^3^*J* = 4.4 Hz, 1H, OC**H**_2_), 2.06 (d, *J* = 6.9 Hz, 3H, CHC**H_3_**); ^13^C NMR (151 MHz, CDCl_3_) δ 156.90, 140.58, 139.53, 135.23, 133.92, 132.48, 131.36, 130.05, 129.17, 127.27, 126.11, 125.32, 125.14, 124.72, 123.30, 122.50, 121.10, 119.55, 119.52, 117.66, 110.36, 108.48, 94.99, 70.58, 63.85, 19.06. Anal. Calcd. for C_26_H_22_N_2_O_2_: C, 79.17; H, 5.62; N, 7.10. Found: C, 79.27; H, 5.58; N, 6.85.

(*S*)-2-[(9*H*-Carbazol-2-yl)oxy]-*N*-[1-(naphthalen-1-yl)ethyl]ethan-1-imine oxide (**11bg**). According to the general procedure from the crude aldehyde **15b** (0.198 g, 0.88 mmol) obtained directly from diol **14b** (0.330 g, 1.25 mmol)], (*S*)-(−)-(1-(1-naphtyl)ethyl)hydroxylamine oxalate (0.268 g, 0.97 mmol), the pure nitrone **11bg** (0.100 g, 20% calculated for two steps, starting from diol **14b**) was obtained as brown powder after 5 h of the reaction and subsequent purification by column chromatography on silica gel (hexane:ethyl acetate, 5:1, *v*/*v*): m.p. 62–65 °C; ${\left[\alpha \right]}_{D}^{20}$ +27.20 (*c* 0.82, CHCl_3_); IR and NMR spectra of (*S*)-**11bg** were identical with its enantiomer (*R*)-**1bf**. Anal. Calcd. for C_26_H_22_N_2_O_2_: C, 79.17; H, 5.62; N, 7.10. Found: C, 79.25; H, 5.52; N, 6.86.

### 3.6. General Biological Assays

Nordihydroguaiaretic acid (NDGA), Trolox, 2,2′-azobis(2-amidinopropane) dihydrochloride (AAPH), 2,2-diphenyl-1-picrylhydrazyl (DPPH), soybean lipoxygenase (LOX), and linoleic acid sodium salt were purchased from Aldrich Chemical Co. (Milwaukee, WI, USA). A 0.1 M phosphate buffer (pH 7.4) was prepared by mixing 50 mL of 0.2 M KH_2_PO_4_ solution with 78 mL of 0.1 M NaOH solution, with the pH adjusted by the addition of KH_2_PO_4_ or NaOH as needed.

The test compounds were dissolved in DMSO to prepare a 10 mM stock solution. All in vitro assays were conducted using a Lambda 20 (Perkin–Elmer-PharmaSpec 1700) UV–Vis double-beam spectrophotometer (Perkin-Elmer Corporation Ltd., Lane Beaconsfield, Bucks, United Kingdom). Each experiment was performed in at least triplicate (mean value ± SD, and the standard deviation (SD) of absorbance values was less than 10% of the mean). The inhibition values are given as % inhibition at 100 µM, or as IC_50_ µM values. For the determination of % inhibition the following equation was used:

Inhibition (%) = [Ac (absorbance of control) − As (absorbance of sample)/Ac (absorbance of control] × 100].

NGA and Trolox were used as positive control/reference compounds, whereas a negative control was also used.

### 3.7. Theoretical Calculation of Lipophilicity Values (Clog P)

Theoretical lipophilicity (Clog P) values were calculated using Bioloom version 1.5 software from Biobyte Corp (available online: http://www.biobyte.com, accessed on 8 September 2026). Lipophilicity is an important physicochemical property influencing biological activity as well as Absorption–Distribution–Metabolism–Excretion–Toxicity (ADMET) properties; therefore, we found it interesting to perform theoretical calculations to assess its potential impact on compound behavior.

### 3.8. Determination of Interaction with DPPH

To a solution of DPPH in absolute ethanol the appropriate volume of the compounds (100 μM final concentrations) dissolved in DMSO was added [46]. The absorbance was recorded at 517 nm after 20 min at room temperature. A color change was observed upon reduction. NDGA was used as a reference compound.

### 3.9. Hydroxyl Radical Scavenging Activity

The hydroxyl radicals were produced by the Fe^3+^/ascorbic acid system. EDTA (0.1 mM), Fe^3+^ (167 μM), DMSO (33 mM) in phosphate buffer (50 mM, pH 7.4), the tested compounds (100 μM) and ascorbic acid (10 mM) were mixed in test tubes. The solutions were incubated at 37 °C for 30 min. The reaction was stopped by CCl_3_COOH (17% *w*/*v*) (Table 1) and the % scavenging activity of the tested compounds for hydroxyl radicals was given [46].

### 3.10. Inhibition of Linoleic Acid Peroxidation

2,2′-azobis(2-amidinopropane) dihydrochloride (AAPH) is used to induce the production of alkylperoxyl radicals. The final solution in the UV cuvette consisted of ten microliters of the 16 mM linoleate sodium dispersion 0.93 mL of 0.05 M phosphate buffer, pH 7.4, thermostated at 37 °C. A total of 50 μL of 40 mM AAPH solution was added as a free radical initiator at 37 °C under air and 10 μL of the tested compounds [50]. The oxidation of linoleic acid sodium salt results in a conjugated diene hydroperoxide. The reaction is monitored at 234 nm (Table 1).

### 3.11. ABTS^•+^—Decolorization Assay in Ethanolic Solution for Antioxidant Activity

ABTS is dissolved in water to a 2 mM concentration. ABTS radical cation (ABTS^•+^) is produced by reacting the ABTS stock solution with 0.17 mM potassium persulfate in phosphate buffer (pH 7.4, 20 mM) and allowing the mixture to stand in the dark at room temperature for 12–16 h before use. For steady state measurements, 100 µM ABTS^•+^ was used. The 100 µM ABTS^•+^ solution (200 μL) was diluted with ethanol (790 μL) to an absorbance of 0.70 at 734 nm, equilibrated at room temperature, mixed with 10 μL of the tested compounds and the absorbance was taken at room temperature 1 min after the initial mixing [51]. Trolox was used as a standard.

### 3.12. Soybean Lipoxygenase Inhibition Assay

To evaluate the inhibition of soybean LOX, 10 µL of the test compound stock solution was added to a UV cuvette at room temperature, along with 100 µL of sodium linoleate and 200 µL of enzyme solution (1.9 × 10^−4^ *w*/*v* in saline) prepared in Tris buffer (pH 9.0). The enzymatic conversion of sodium linoleate to 13-hydroperoxylinoleic acid was monitored by measuring absorbance at 234 nm [47]. Three to six different concentrations up to 100 µM were used for the determination of IC_50_ values. For compound **11be**, the five most potent inhibitor concentrations were used (0.01, 0.02, 0.03, 0.08 and 0.1 µM) (concentration–response curves for all compounds for which IC_50_ values were determined are provided in the Supplementary Material). NDGA was used as the reference inhibitor.

## 4. Conclusions

We report the synthesis, antioxidant, and anti-inflammatory properties of fourteen carbazole–nitrone hybrids. Their antioxidant activity was studied by different assays, including inhibition of lipid peroxidation induced by AAPH, the hydroxyl radical scavenging assay, ABTS^•+^–decolorization assay and DPPH scavenging assay. Their anti-inflammatory activity was determined by the inhibition of soybean lipoxygenase. Extensive evaluation highlighted nitrone **11bg** as the most potent antioxidant, combining high anti-lipid peroxidation activity and scavenging of hydroxyl radicals. Nitrone **11be** was found to be the most potent lipoxygenase inhibitor (IC_50_ = 0.0225 µM). (*S*)-Specificity is related with higher inhibition. However, it is interesting that **11bd** the (*R*) enantiomer is also potent. Seven out of fourteen hybrids highly inhibit SLOX, with IC_50_ values less than 30 μM.

Altogether, these results pave the way for further in-depth studies of carbazole nitrone **11bg** as well as **11be** in vitro and in vivo.

## Figures and Tables

**Table 1:** Antioxidant activity of **11aa**–**11ag** and **11ba**–**11bg** ^a^ and standards Trolox and NDGA.

Nitrone ^a^/ Standard	Clog *P* ^b^	DPPH (% ±SD)	ILP (% ±SD)	LOX Inhibition (Inhibition % at 100 μM or IC_50_ [μM ± SD])	Scavenging Activity for ^•^OH (% ± SD)	Scavenging of ABTS^•+^ (% ± SD)
**11aa**	1.69	39 (0.5)	88.3 (1.9)	25 μM (0.8)	70 (2.3)	51 (1.1)
**11ab**	3.84	34 (0.9)	65.6 (2.1)	25.5 μM (1.2)	91 (2.7)	50 (1.9)
**11ac**	3.40	25.5 (0.6)	63.8 (1.7)	45 μM (2.0)	no ^c^	48 (1.3)
**11ad**	3.71	54.5 (1.1)	no ^c^	19.5 μM (0.9)	71 (1.8)	49 (1.3)
**11ae**	3.71	52 (2.1)	10.1 (0.03)	30% (1.6)	72.6 (2.4)	49 (1.1)
**11af**	4.88	44 (1.7)	63.8 (1.1)	66 μM (2.3)	68.4 (1.7)	42 (0.7)
**11ag**	4.88	25.4 (1.0)	48.7 (0.8)	17.5 μM (1.5)	no ^c^	45 (0.6)
**11ba**	1.69	24.5 (0.4)	52.2 (1.5)	71.5 μM (2.2)	42 (1.3)	48 (0.3)
**11bb**	3.84	15 (0.08)	47.2 (1.3)	33.4 μM (0.8)	71.6 (1.2)	49 (0.5)
**11bc**	3.40	21 (0.3)	no ^c^	38% (1.2)	76.7 (1.3)	35 (0.9)
**11bd**	3.71	20.6 (0.1)	100 (2.9)	6.6 μM (0.3)	55 (0.9)	85 (2.4)
**11be**	3.71	11 (0.06)	85 (2.1)	0.0225 μM (0.001)	75 (1.8)	46 (1.1)
**11bf**	4.88	38.8 (1.2)	80.6 (2.7)	50 μM (1.2)	10.7 (0.7)	87.8 (2.1)
**11bg**	4.88	89.7 (2.6)	78.8 (1.9)	26 μM (0.7)	89 (2.6)	95 (3.1)
**NDGA**		97 (1.8)	- ^d^	0.45 μM (0.02)	- ^d^	- ^d^
**Trolox**			93 (3.2)	- ^d^	73 (2.2)	91 (2.8)

^a^ Nitrones tested at 100 µM. ^b^ BioByte Corporation, C-QSAR database, 201 W Fourth St., Suite #204, Claremont, CA 91711-4707, USA. Values are means of three or four different determinations. no = no activity under the experimental conditions. Means within each column differ significantly (*p* < 0.05). ^c^ no = no activity was determined under the reported conditions; ^d^ not determined.

## Data Availability

The original contributions presented in this study are included in the article/Supplementary Materials. Further inquiries can be directed to the corresponding author.

## Supplementary Materials

The following supporting information can be downloaded at https://www.mdpi.com/article/10.3390/molecules31203619/s1. Figure S1: ^1^H NMR Spectrum for **13a** in CDCl_3_; Figure S2: ^1^H NMR Spectrum for **13b** in DMSO-*d*_6_; Figure S3: ^1^H NMR Spectrum for **14a** in DMSO-*d*_6_; Figure S4: ^13^C NMR Spectrum for **14a** in DMSO-*d*_6_; Figure S5: ^1^H NMR Spectrum for **14b** in CD_3_OD; Figure S6: ^13^C NMR Spectrum for **14b** in CD_3_OD; Figure S7: ^1^H NMR Spectrum for **15a** in DMSO-*d*_6_; Figure S8: ^1^H NMR Spectrum for **15b** in DMSO-*d*_6_; Figure S9: ^1^H NMR Spectrum for **11aa** in DMSO-*d*_6_. Figure S10: ^13^C NMR Spectrum for **11aa** in DMSO-*d*_6_; Figure S11: ^1^H NMR Spectrum for **11ab** in CDCl_3_; Figure S12: ^13^C NMR Spectrum for **11ab** in CDCl_3_; Figure S13: ^1^H NMR Spectrum for **11ac** in DMSO-*d_6_*; Figure S14: ^13^C NMR Spectrum for **11ac** in DMSO-*d*_6_; Figure S15: ^1^H NMR Spectrum for **11ad** in CDCl_3_; Figure S16: ^13^C NMR Spectrum for **11ad** in CDCl_3_; Figure S17: ^1^H NMR Spectrum for **11ae** in CDCl_3_; Figure S18: ^13^C NMR Spectrum for **11ae** in CDCl_3_; Figure S19: ^1^H NMR Spectrum for **11af** in CDCl_3_; Figure S20: ^13^C NMR Spectrum for **11af** in CDCl_3_; Figure S21: ^1^H NMR Spectrum for **11ag** in CDCl_3_; Figure S22: ^13^C NMR Spectrum for **11ag** in CDCl_3_; Figure S23: ^1^H NMR Spectrum for **11ba** in DMSO-*d*_6_; Figure S24: ^13^C NMR Spectrum for **11ba** in DMSO-*d*_6_; Figure S25: ^1^H NMR Spectrum for **11bb** in CDCl_3_; Figure S26: ^13^C NMR Spectrum for **11bb** in CDCl_3_; Figure S27: ^1^H NMR Spectrum for **11bc** in DMSO-*d*_6_; Figure S28: ^13^C NMR Spectrum for **11bc** in DMSO-*d*_6_; Figure S29: ^1^H NMR Spectrum for **11bd** in DMSO-*d*_6_; Figure S30: ^13^C NMR Spectrum for **11bd** in DMSO-*d*_6_; Figure S31: ^1^H NMR Spectrum for **11be** in DMSO-*d*_6_; Figure S32: ^13^C NMR Spectrum for **11be** in DMSO-*d*_6_; Figure S33: ^1^H NMR Spectrum for **11bf** in CDCl_3_; Figure S34: ^13^C NMR Spectrum for **11bf** in CDCl_3_; Figure S35: ^1^H NMR Spectrum for **11bg** in CDCl_3_; Figure S36: ^13^C NMR Spectrum for **11bg** in CDCl_3_; Figure S37: Concentration–response curve for determination of the IC_50_ of nitrone **11aa**; Figure S38: Concentration–response curve for determination of the IC_50_ of nitrone **11ab**; Figure S39: Concentration–response curve for determination of the IC_50_ of nitrone **11ac**; Figure S40: Concentration–response curve for determination of the IC_50_ of nitrone **11ad**; Figure S41: Concentration–response curve for determination of the IC_50_ of nitrone **11af**; Figure S42: Concentration–response curve for determination of the IC_50_ of nitrone **11ag**; Figure S43: Concentration–response curve for determination of the IC_50_ of nitrone **11ba**; Figure S44: Concentration–response curve for determination of the IC_50_ of nitrone **11bb**; Figure S45: Concentration–response curve for determination of the IC_50_ of nitrone **11bd**; Figure S46: Concentration–response curve for determination of the IC_50_ of nitrone **11be**; Figure S47: Concentration–response curve for determination of the IC_50_ of nitrone **11bf**; Figure S48: Concentration–response curve for determination of the IC_50_ of nitrone **11bg**; Figure S49: Concentration–response curve for determination of the IC_50_ of NDGA.
