# Supplementary material for: Antioxidant Activity of a New Series of Carbazole-Derived Nitrones
Source: Molecules. 2026 Oct 11;31(20):3619. doi: 10.3390/molecules31203619 (PMC13707573; doi:10.3390/molecules31203619)

Supplementary Materials

Contents

Figure S1: ^{1}H NMR Spectrum for **13a** in $CDCl_3$.

Figure S2: ^{1}H NMR Spectrum for **13b** in DMSO-d_6.

Figure S3: ^{1}H NMR Spectrum for **14a** in DMSO-d_6.

Figure S4: ^{13}C NMR Spectrum for **14a** in DMSO-d_6.

Figure S5: ^{1}H NMR Spectrum for **14b** in CD_3OD.

Figure S6: ^{13}C NMR Spectrum for **14b** in CD_3OD.

Figure S7: ^{1}H NMR Spectrum for **15a** in DMSO-d_6.

Figure S8: ^{1}H NMR Spectrum for **15b** in DMSO-d_6.

Figure S9: ^{1}H NMR Spectrum for **11aa** in DMSO-d_6.

Figure S10: ^{13}C NMR Spectrum for **11aa** in DMSO-d_6.

Figure S11: ^{1}H NMR Spectrum for **11ab** in $CDCl_3$.

Figure S12: ^{13}C NMR Spectrum for **11ab** in $CDCl_3$.

Figure S13: ^{1}H NMR Spectrum for **11ac** in DMSO-d_6.

Figure S14: ^{13}C NMR Spectrum for **11ac** in DMSO-d_6.

Figure S15: ^{1}H NMR Spectrum for **11ad** in $CDCl_3$.

Figure S16: ^{13}C NMR Spectrum for **11ad** in $CDCl_3$.

Figure S17: ^{1}H NMR Spectrum for **11ae** in $CDCl_3$.

Figure S18: ^{13}C NMR Spectrum for **11ae** in $CDCl_3$.

Figure S19: ^{1}H NMR Spectrum for **11af** in $CDCl_3$.

Figure S20: ^{13}C NMR Spectrum for **11af** in $CDCl_3$.

Figure S21: ^{1}H NMR Spectrum for **11ag** in $CDCl_3$.

Figure S22: ^{13}C NMR Spectrum for **11ag** in $CDCl_3$.

Figure S23: ^{1}H NMR Spectrum for **11ba** in DMSO-d_6.

Figure S24: ^{13}C NMR Spectrum for **11ba** in DMSO-d_6.

Figure S25: ^{1}H NMR Spectrum for **11bb** in $CDCl_3$.

Figure S26: ^{13}C NMR Spectrum for **11bb** in $CDCl_3$.

Figure S27: ^{1}H NMR Spectrum for **11bc** in DMSO-d_6.

Figure S28: ^{1}3C NMR Spectrum for **11bc** in DMSO-d_6.

Figure S29: ^{1}H NMR Spectrum for **11bd** in DMSO-d_6.

Figure S30: ^{13}C NMR Spectrum for **11bd** in DMSO-d_6.

Figure S31: ^{1}H NMR Spectrum for **11be** in DMSO-d_6.

Figure S32: ^{13}C NMR Spectrum for **11be** in DMSO-d_6.

Figure S33: ^{1}H NMR Spectrum for **11bf** in $CDCl_3$.

Figure S34: ^{13}C NMR Spectrum for **11bf** in $CDCl_3$.

Figure S35: ^{1}H NMR Spectrum for **11bg** in $CDCl_3$.

Figure S36: ^{13}C NMR Spectrum for **11bg** in $CDCl_3$.

Figure S37: Concentration-response curve for determination of the IC_{50} of nitrone **11aa**.

Figure S38: Concentration-response curve for determination of the IC_{50} of nitrone **11ab**.

Figure S39: Concentration-response curve for determination of the IC_{50} of nitrone **11ac**.

Figure S40: Concentration-response curve for determination of the IC_{50} of nitrone **11ad**.

Figure S41: Concentration-response curve for determination of the IC_{50} of nitrone **11af**.

Figure S42: Concentration-response curve for determination of the IC_{50} of nitrone **11ag**.

Figure S43: Concentration-response curve for determination of the IC_{50} of nitrone **11ba**.

Figure S44: Concentration-response curve for determination of the IC_{50} of nitrone **11bb**.

Figure S45: Concentration-response curve for determination of the IC_{50} of nitrone **11bd**.

Figure S46: Concentration-response curve for determination of the IC_{50} of nitrone **11be**.

Figure S47: Concentration-response curve for determination of the IC_{50} of nitrone **11bf**.

Figure S48: Concentration-response curve for determination of the IC_{50} of nitrone **11bg**.

Figure S49: Concentration-response curve for determination of the IC_{50} of NDGA.

Figure S1: ^{1}H NMR Spectrum for **13a** in $CDCl_3$.

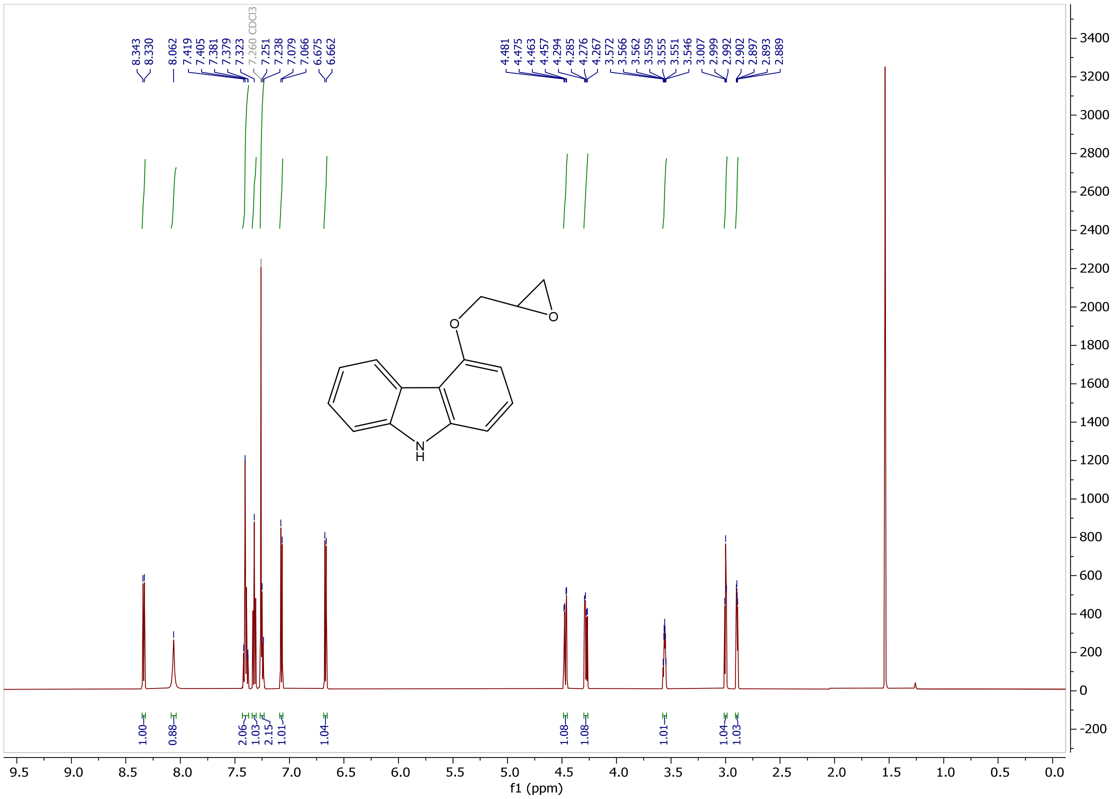

Figure S2: 1H NMR Spectrum for **13b** in DMSO-*d6*.

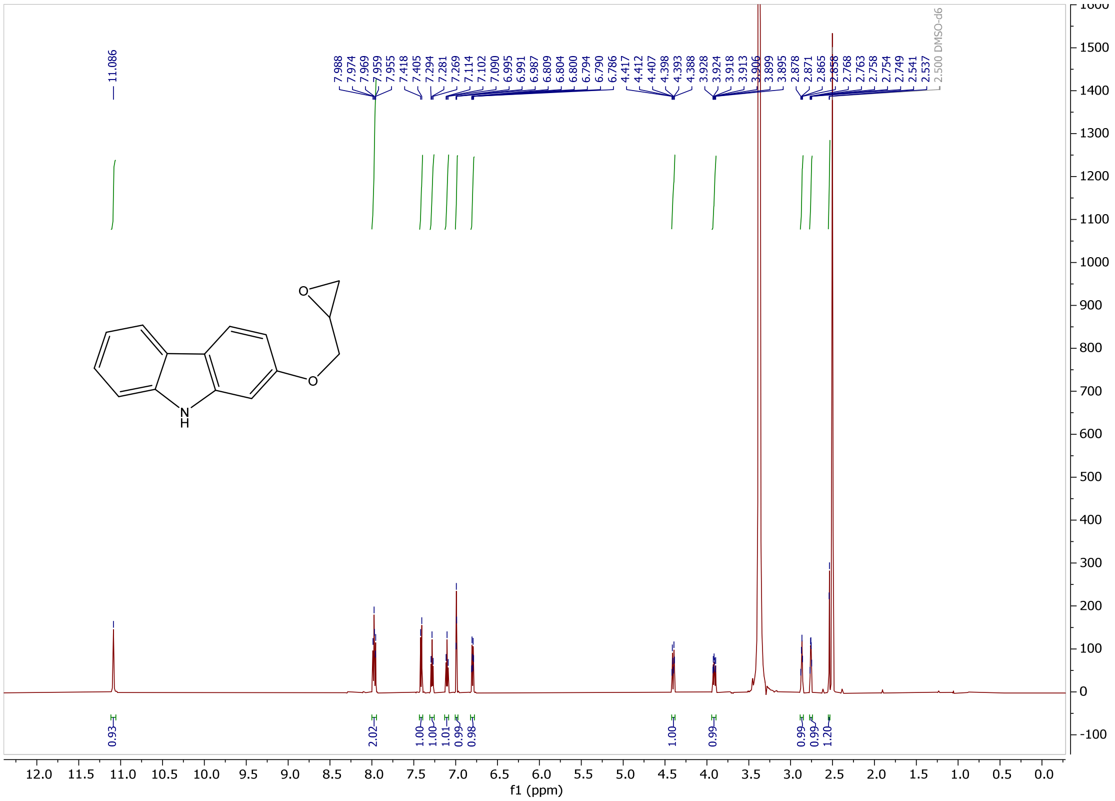

Figure S3: ^{1}H NMR Spectrum for **14a** in DMSO-d_6.

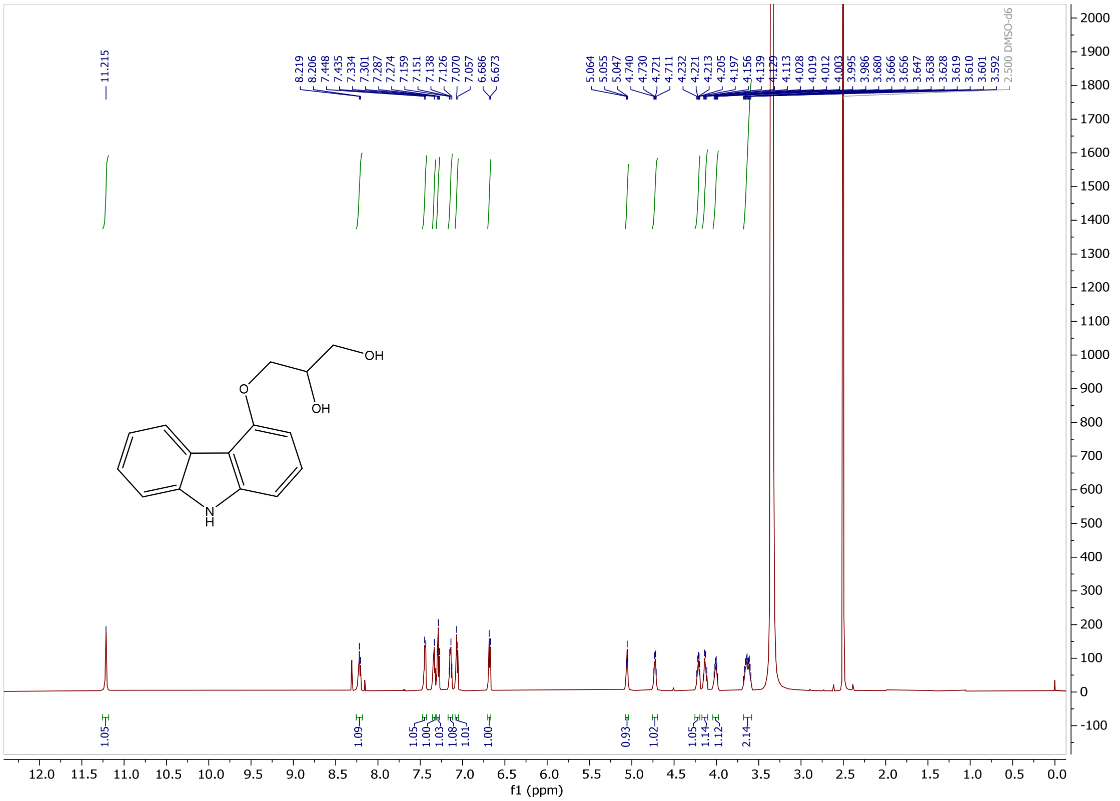

Figure S4: ^{13}C NMR Spectrum for **14a** in DMSO-d_6.

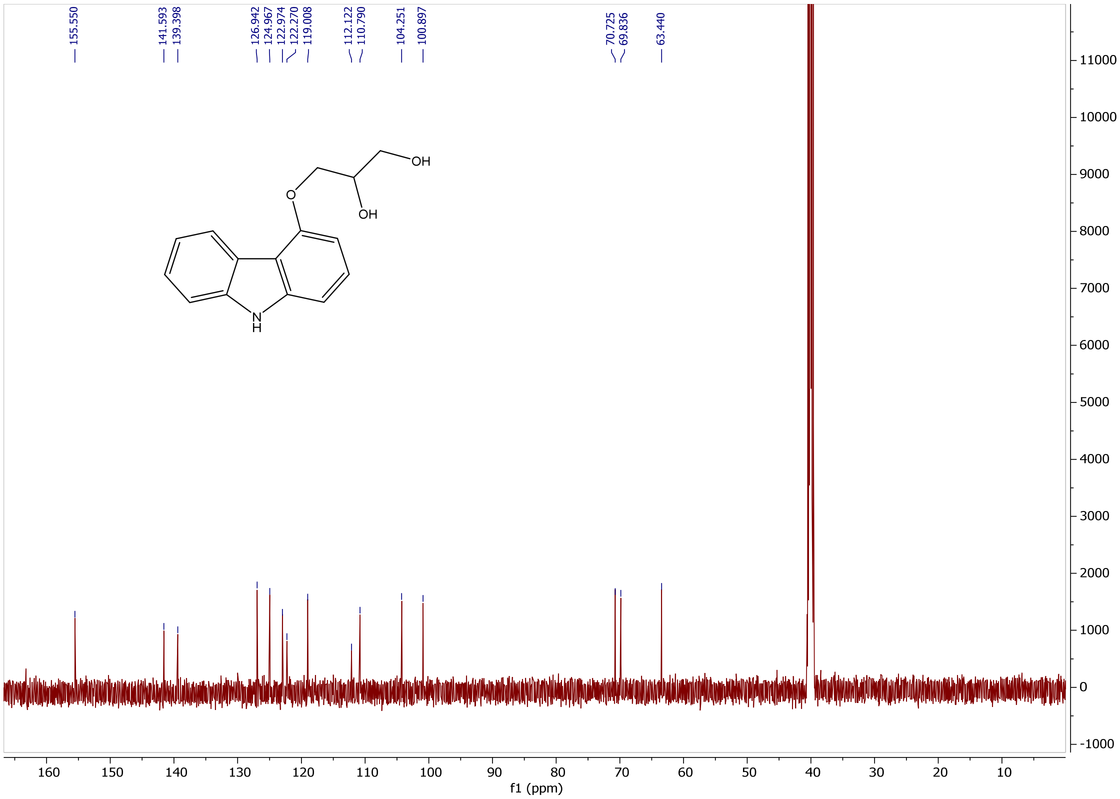

Figure S5: ^{1}H NMR Spectrum for **14b** in CD_3OD.

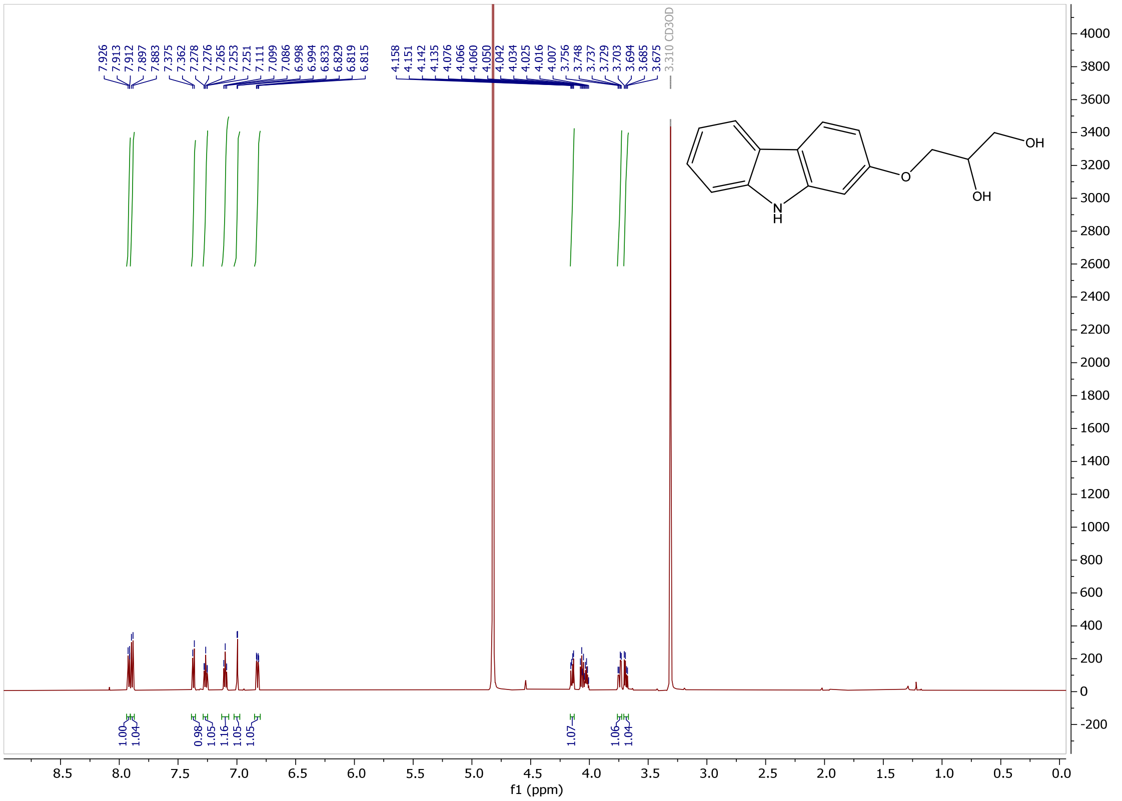

Figure S6: ^{13}C NMR Spectrum for **14b** in CD_3OD.

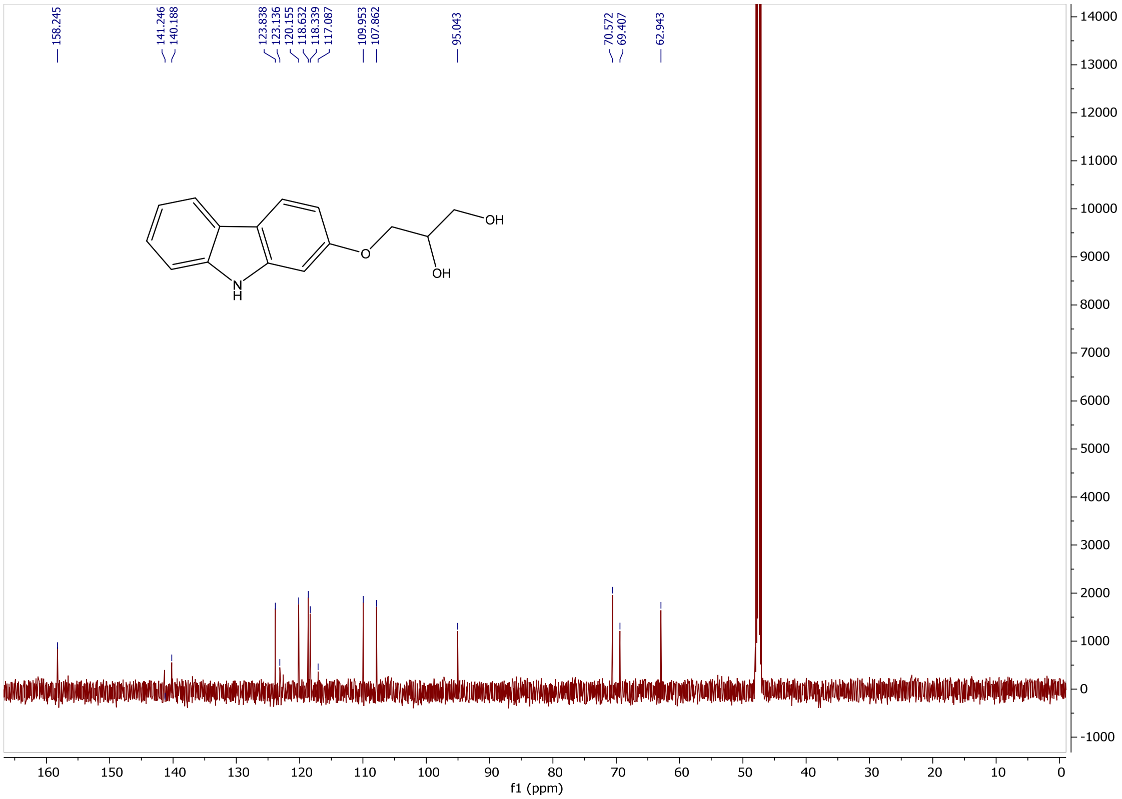

Figure S7: ^{1}H NMR Spectrum for **15a** in DMSO-*d6.*

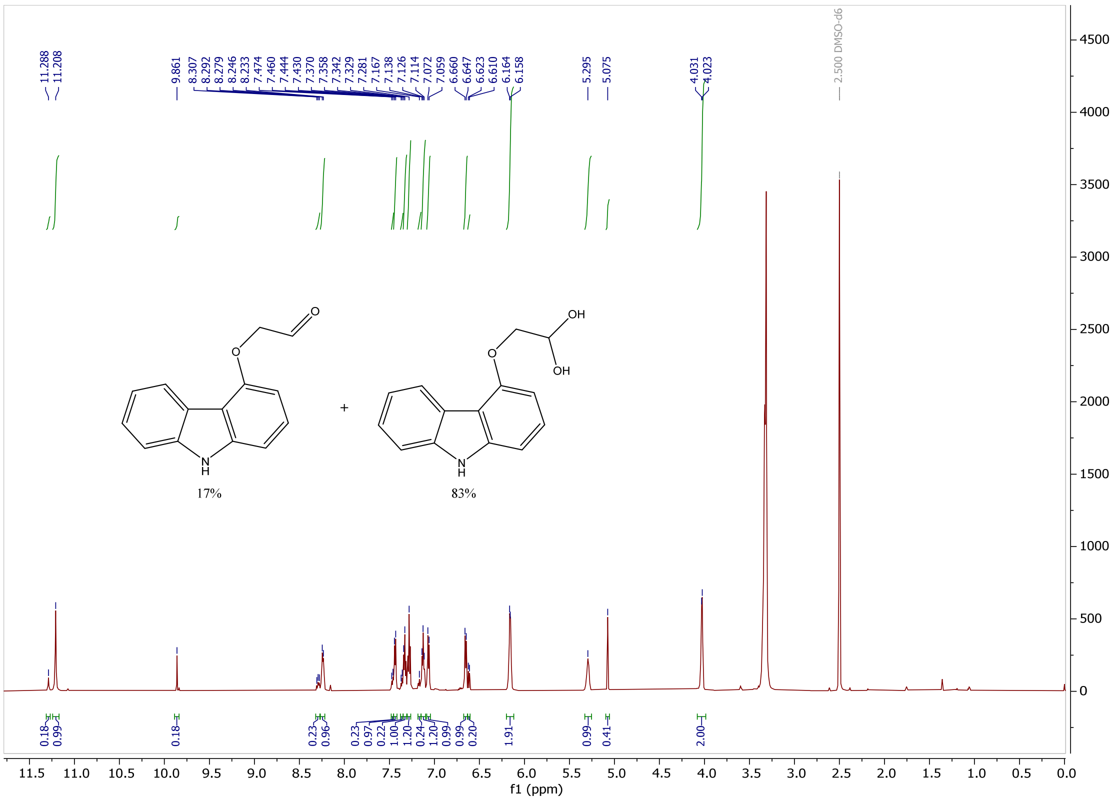

Figure S8: ^{1}H NMR Spectrum for **15b** in DMSO-d_6.

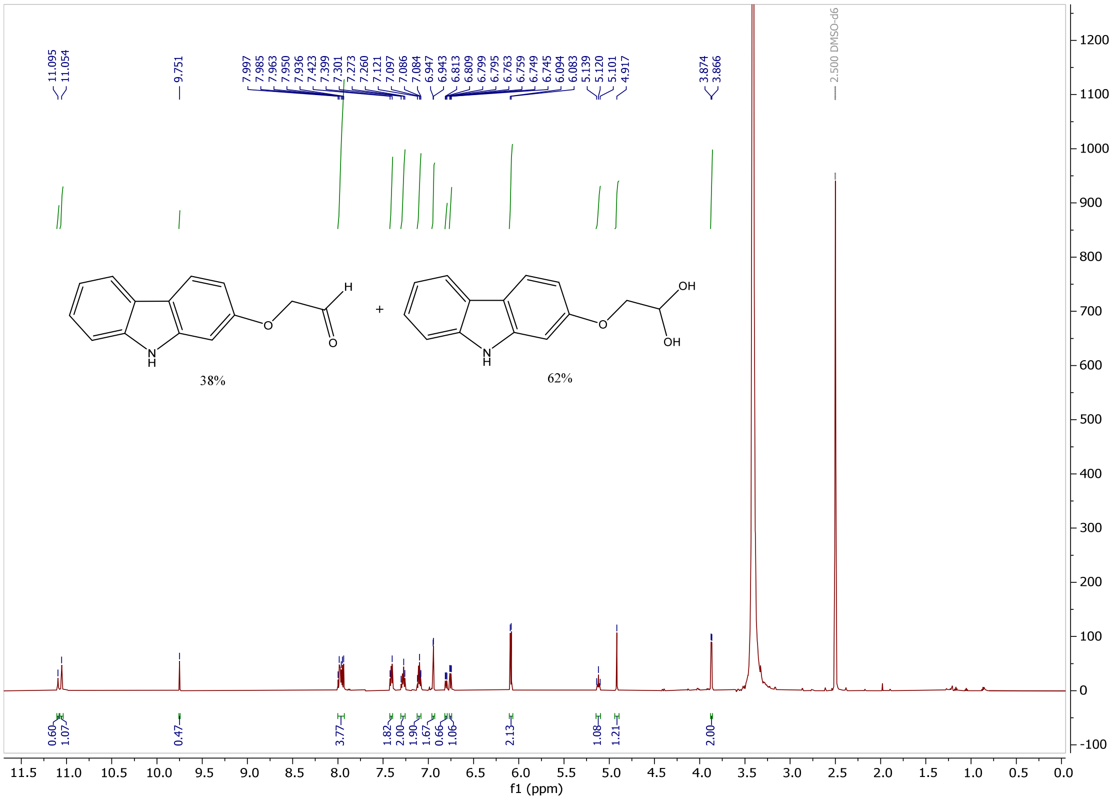

Figure S9: ^{1}H NMR Spectrum for **11aa** in DMSO-d_6.

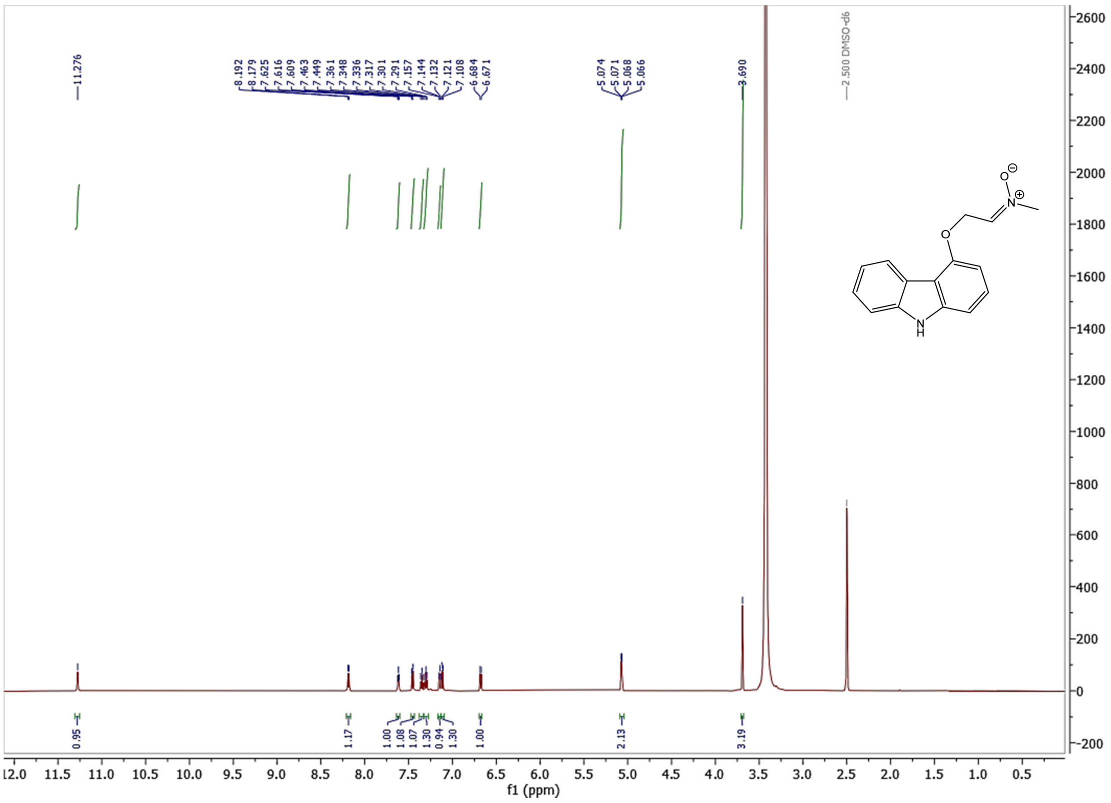

Figure S10: ^{13}C NMR Spectrum for **11aa** in DMSO-d_6.

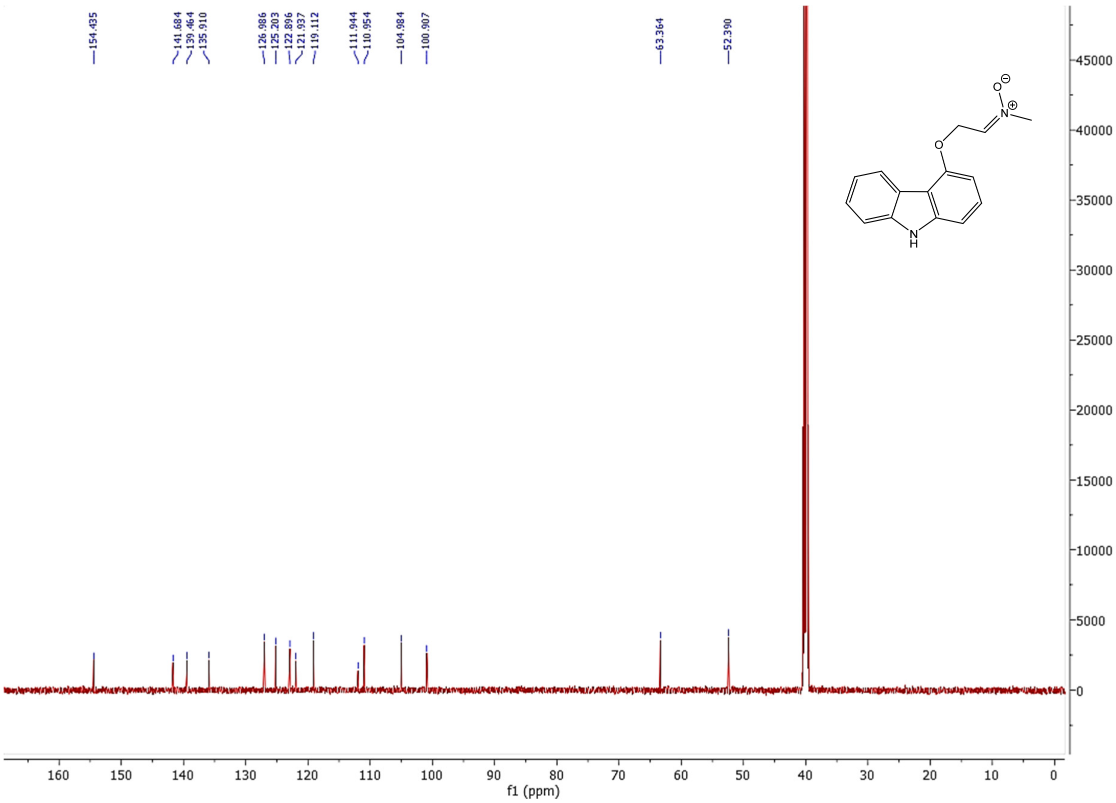

Figure S11: ^{1}H NMR Spectrum for **11ab** in $CDCl_3$.

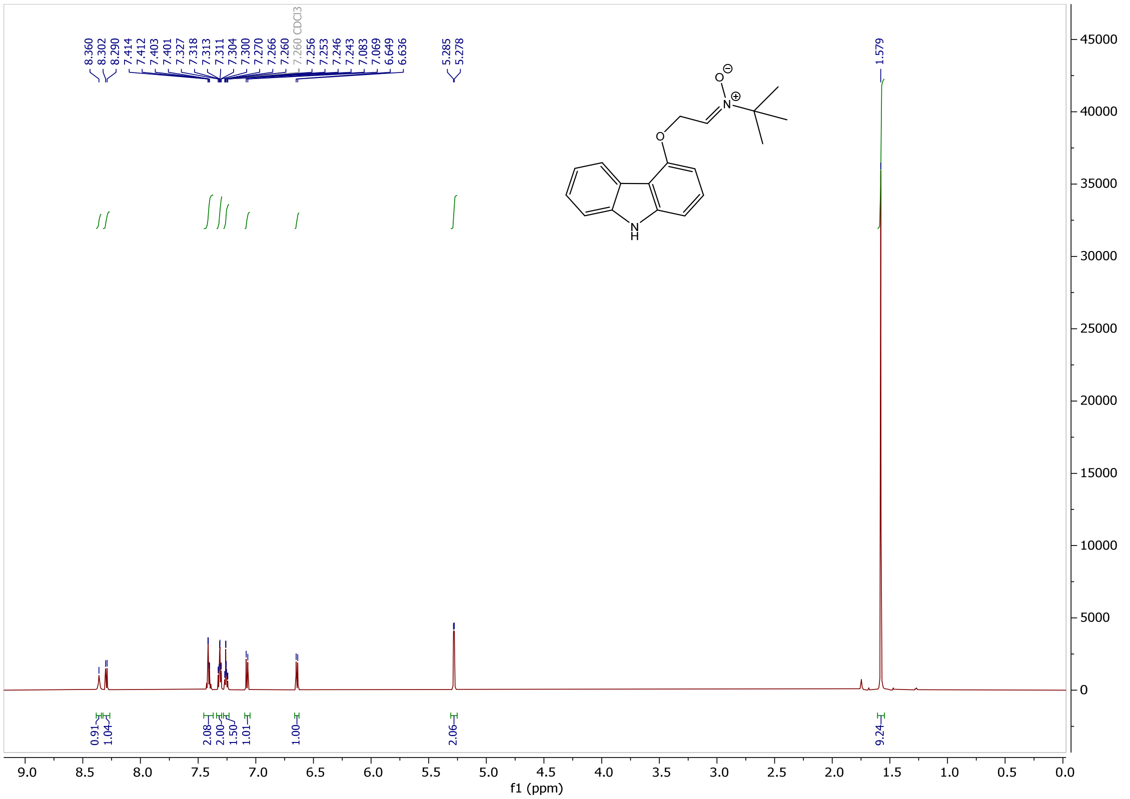

Figure S12: ^{13}C NMR Spectrum for **11ab** in $CDCl_3$.

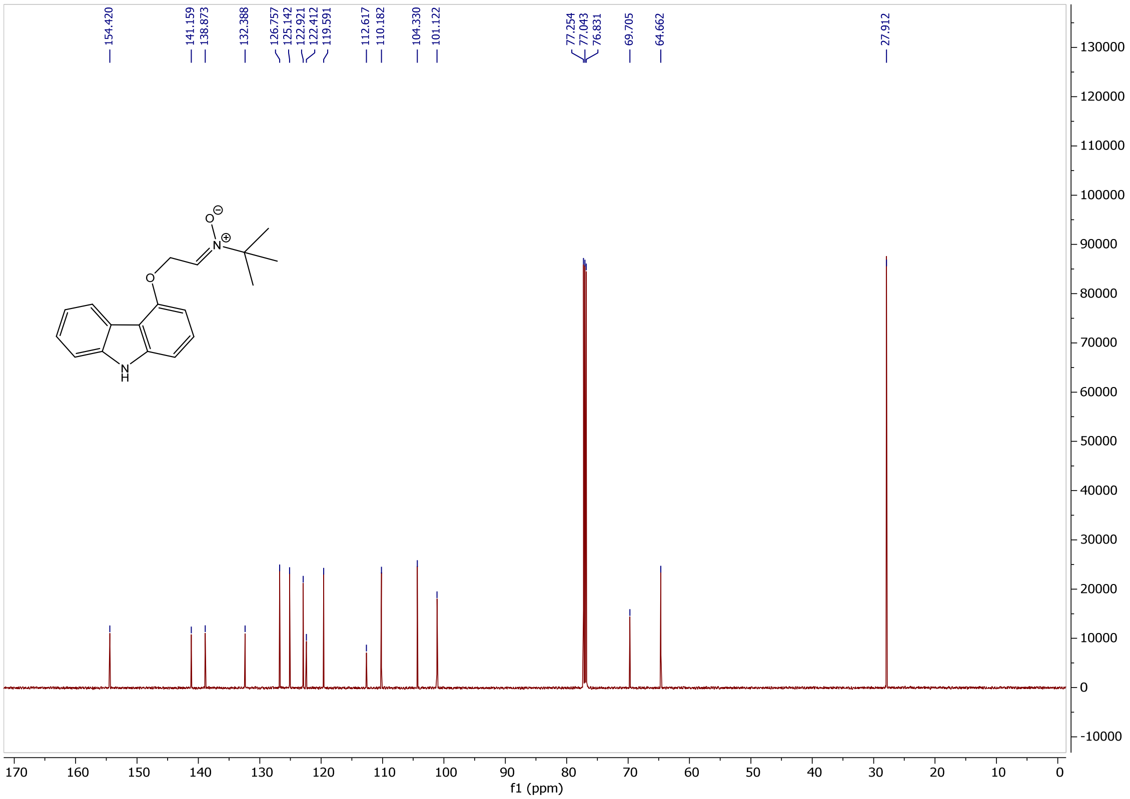

Figure S13: ^{1}H NMR Spectrum for **11ac** in DMSO-d_6.

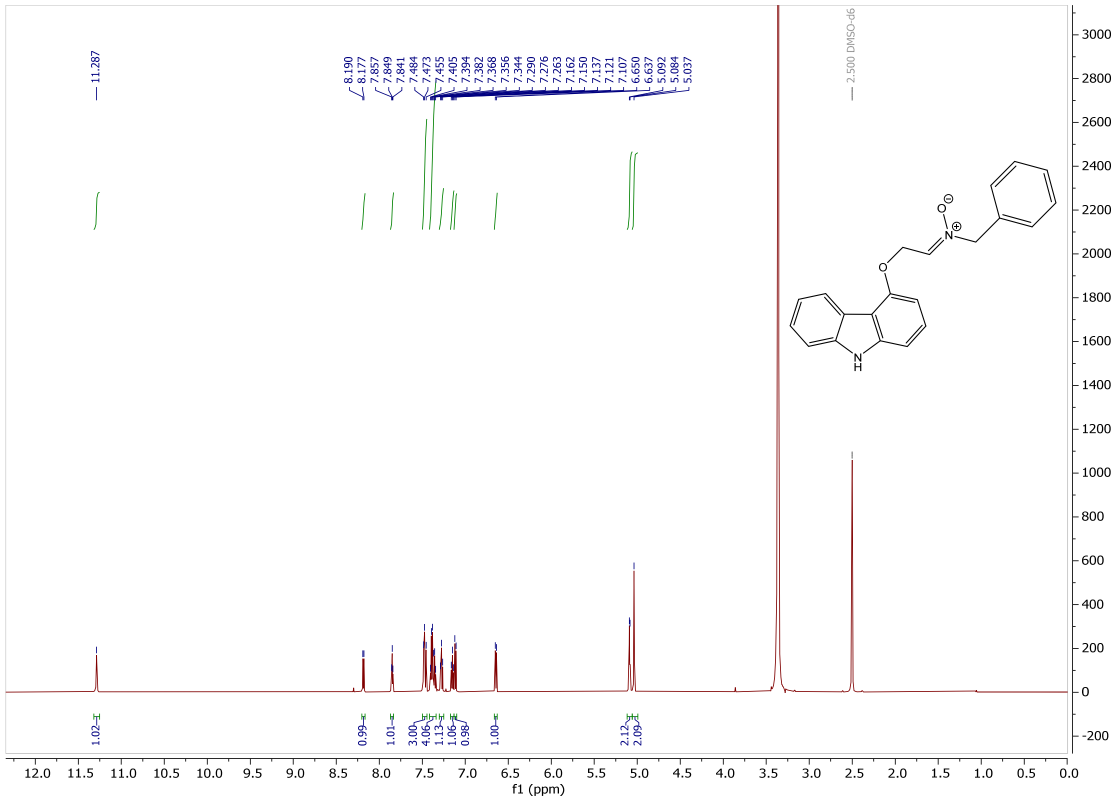

Figure S14: ^{13}C NMR Spectrum for **11ac** in DMSO-d_6.

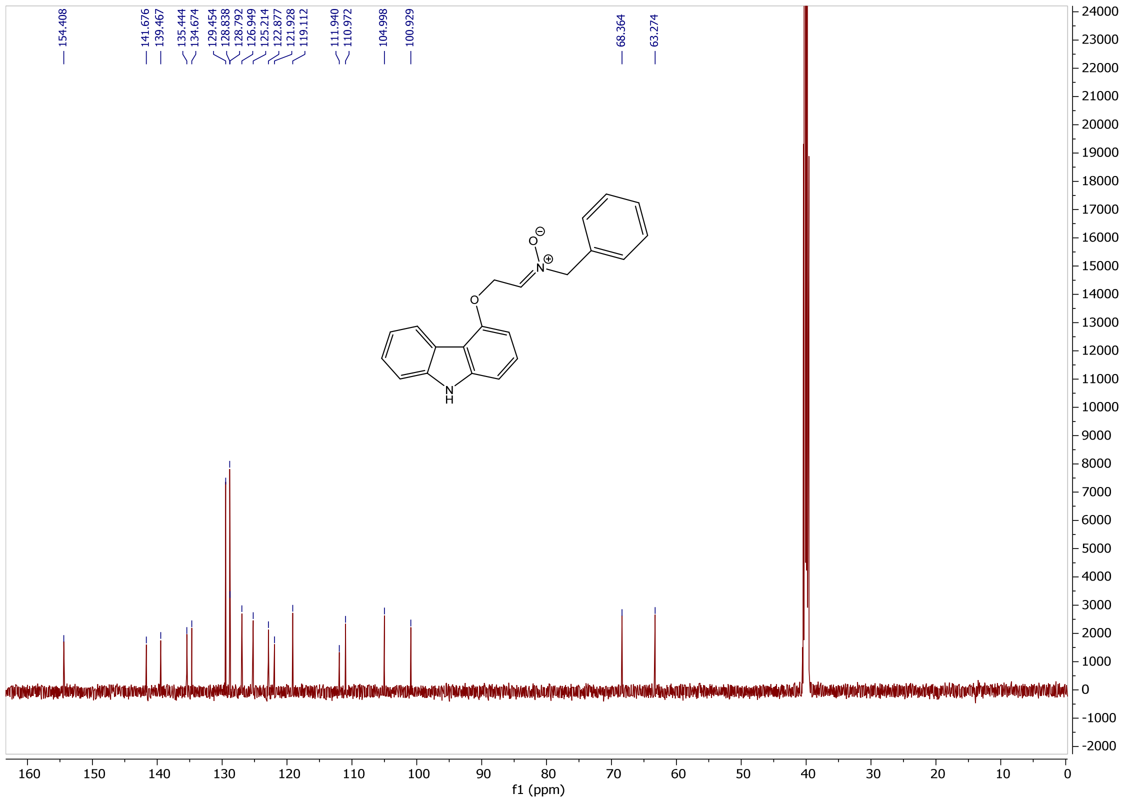

Figure S15: ^{1}H NMR Spectrum for **11ad** in $CDCl_3$.

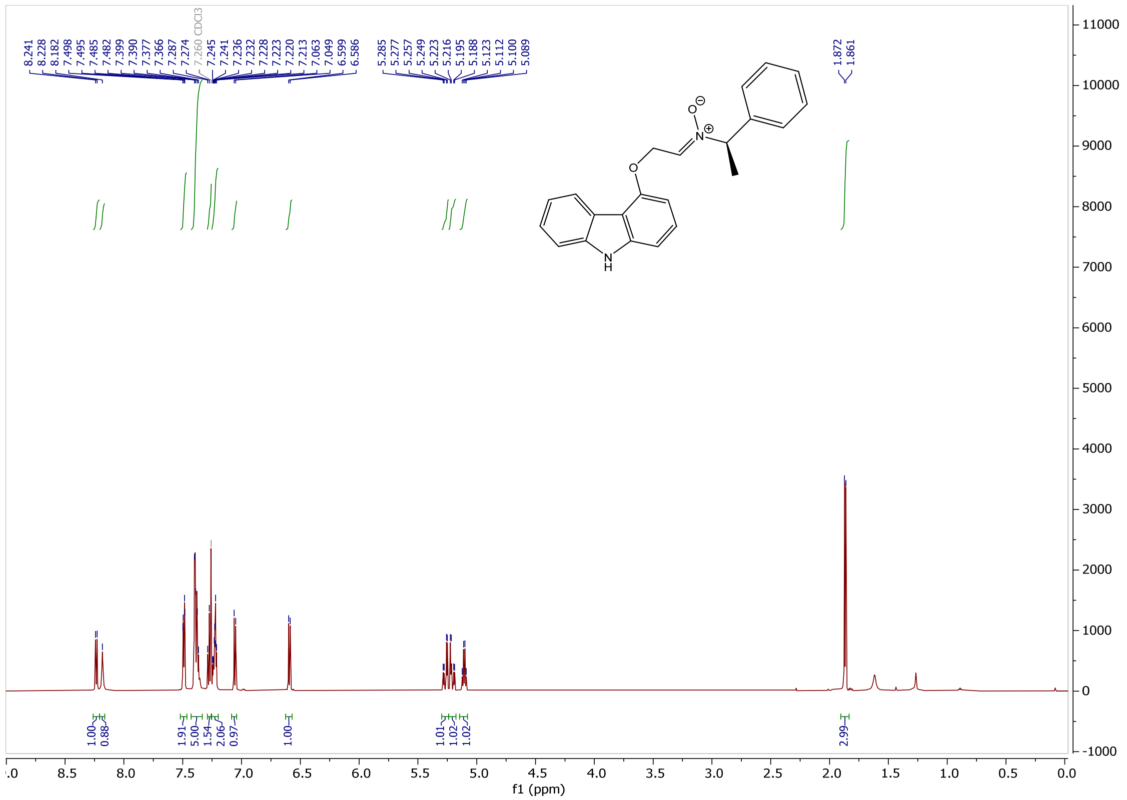

Figure S16: ^{13}C NMR Spectrum for **11ad** in $CDCl_3$.

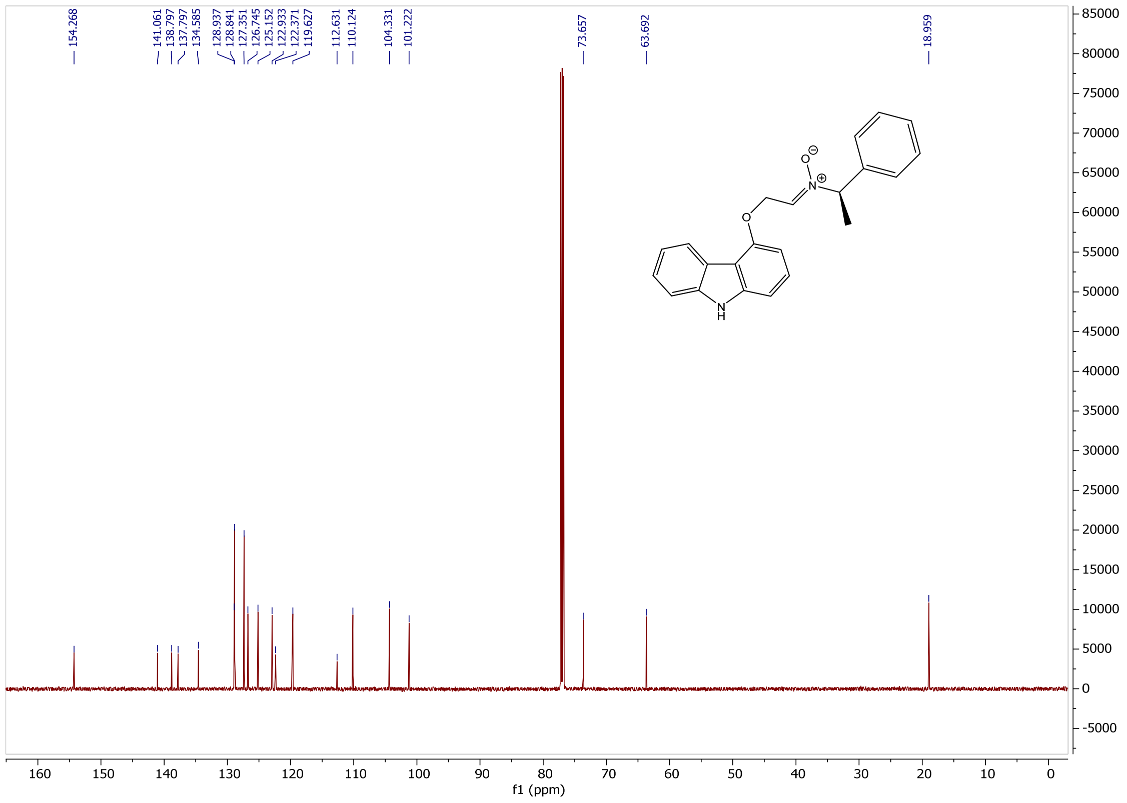

Figure S17: ^{1}H NMR Spectrum for **11ae** in $CDCl_3$.

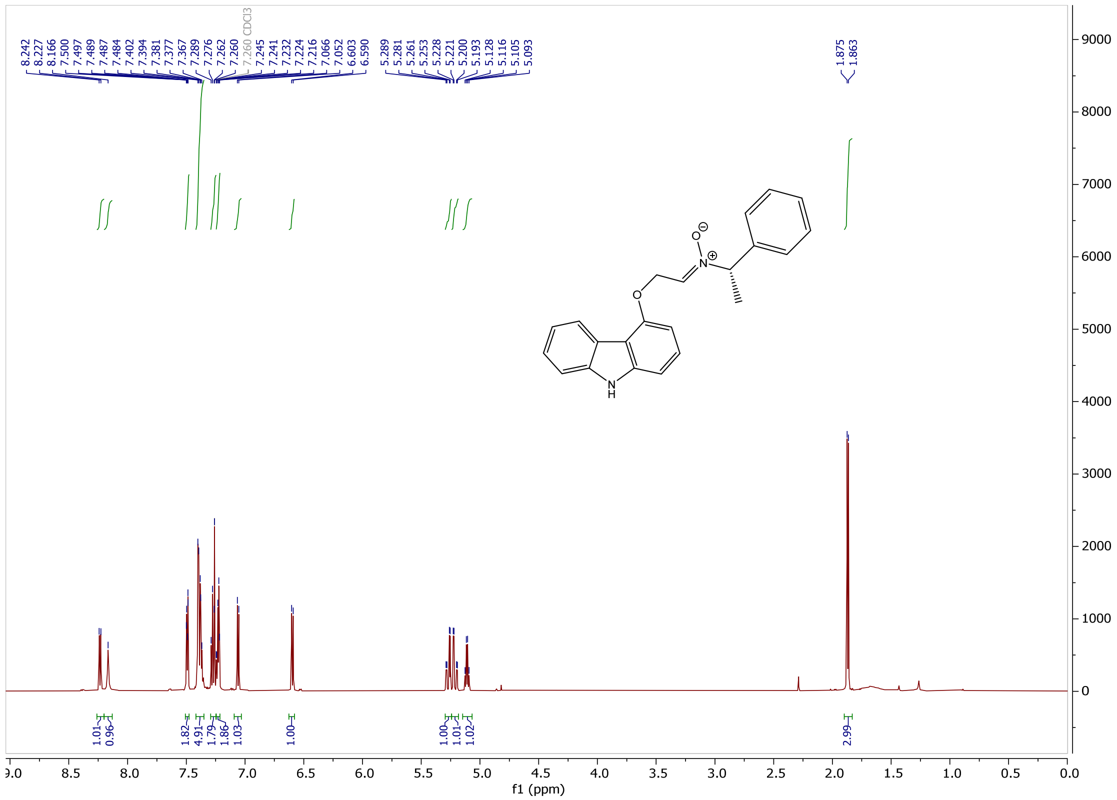

Figure S18: ^{13}C NMR Spectrum for **11ae** in $CDCl_3$.

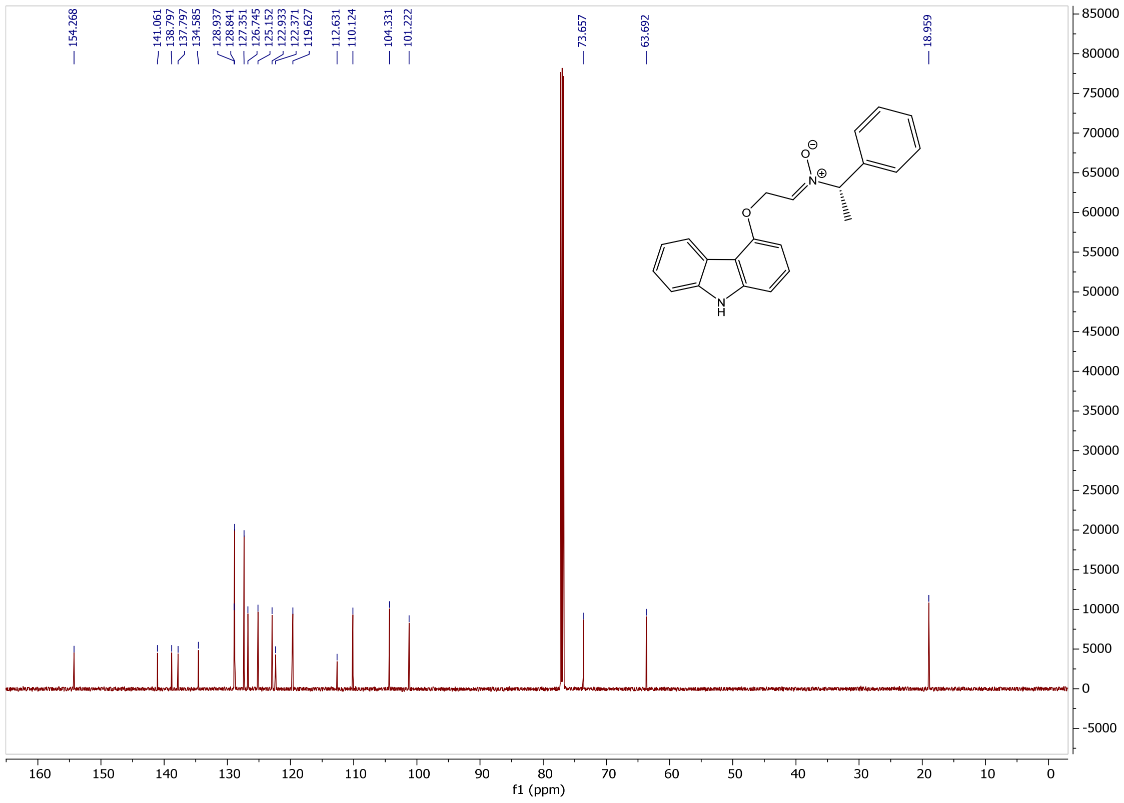

Figure S19: ^{1}H NMR Spectrum for **11af** in $CDCl_3$.

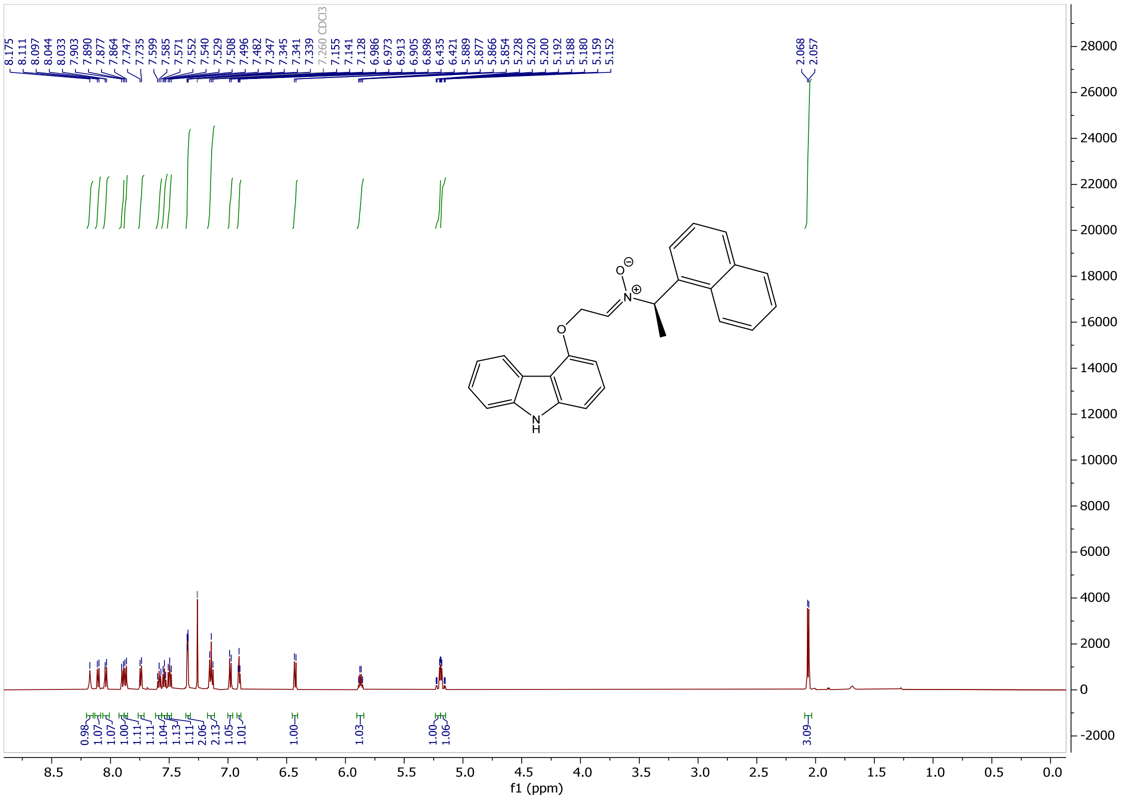

Figure S20: ^{13}C NMR Spectrum for **11af** in $CDCl_3$.

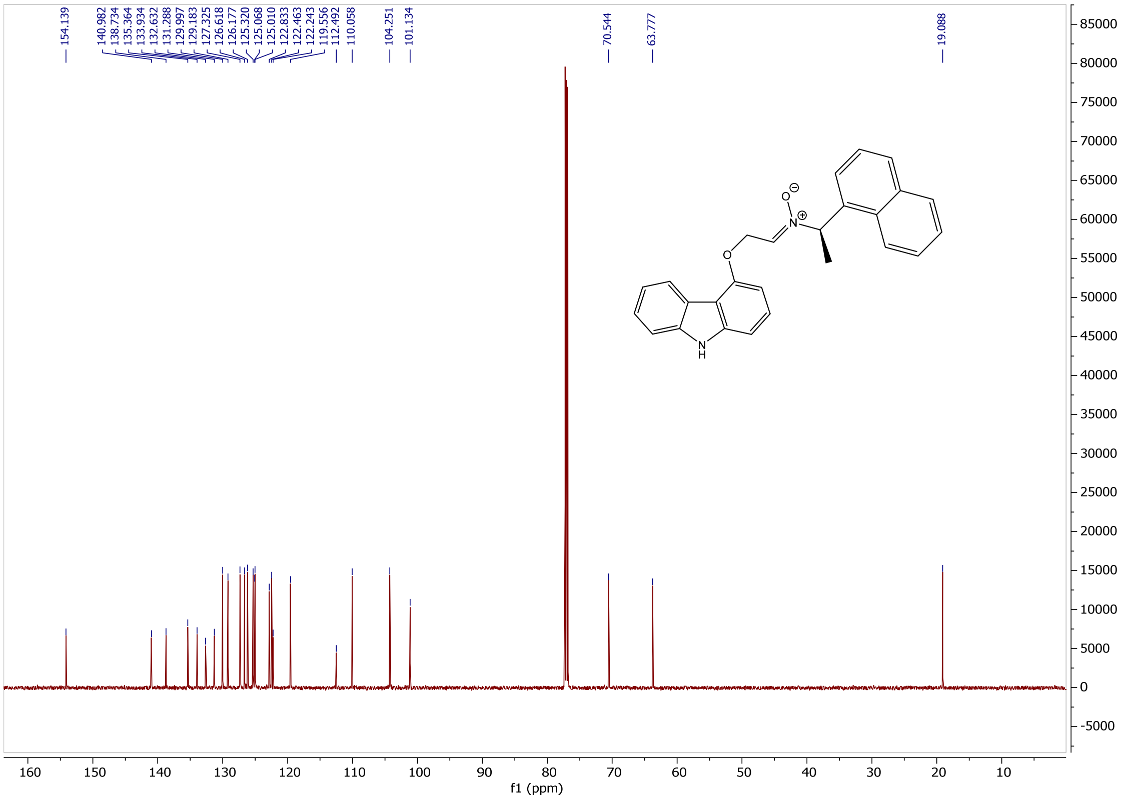

Figure S21: ^{1}H NMR Spectrum for **11ag** in $CDCl_3$.

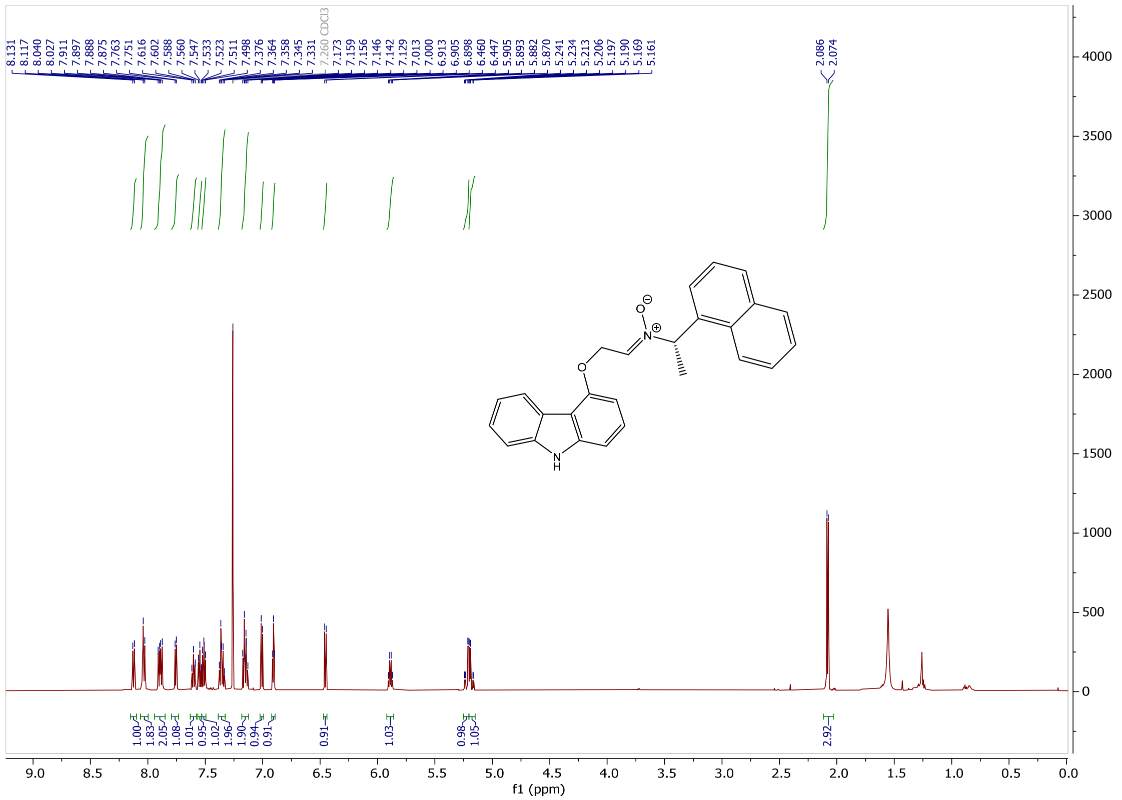

Figure S22: ^{13}C NMR Spectrum for **11ag** in $CDCl_3$.

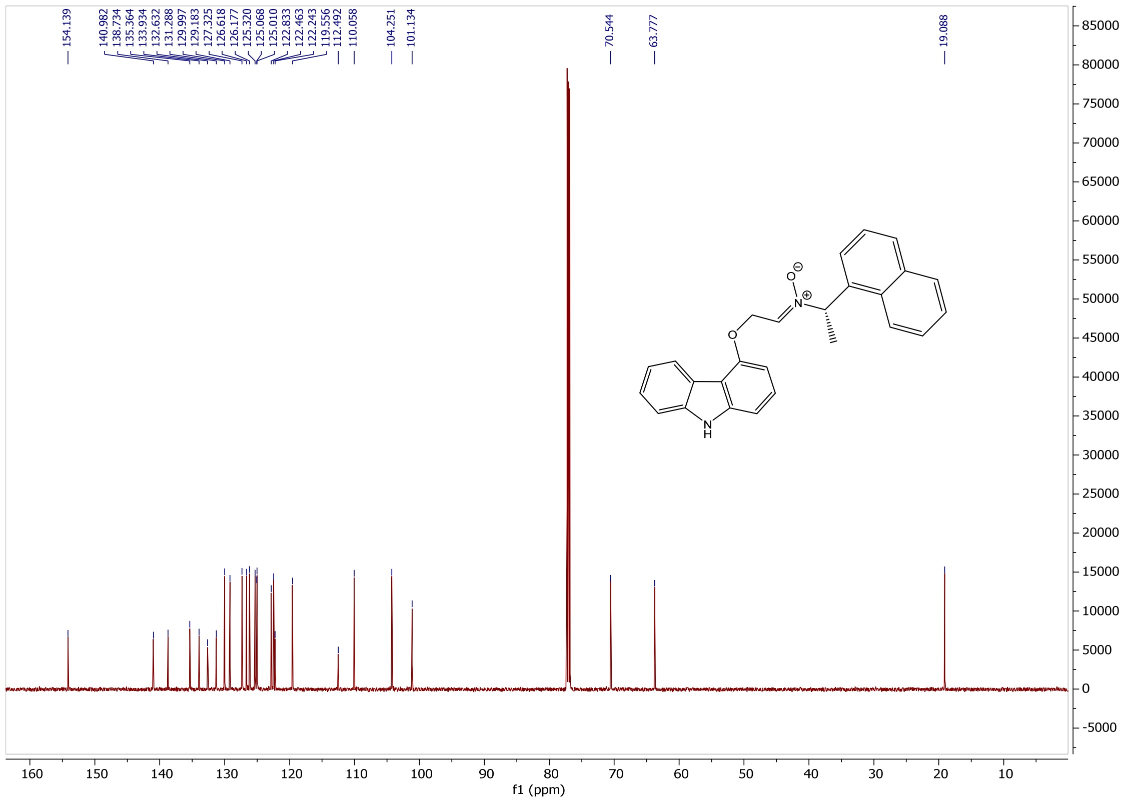

Figure S23: ^{1}H NMR Spectrum for **11ba** in DMSO-d_6.

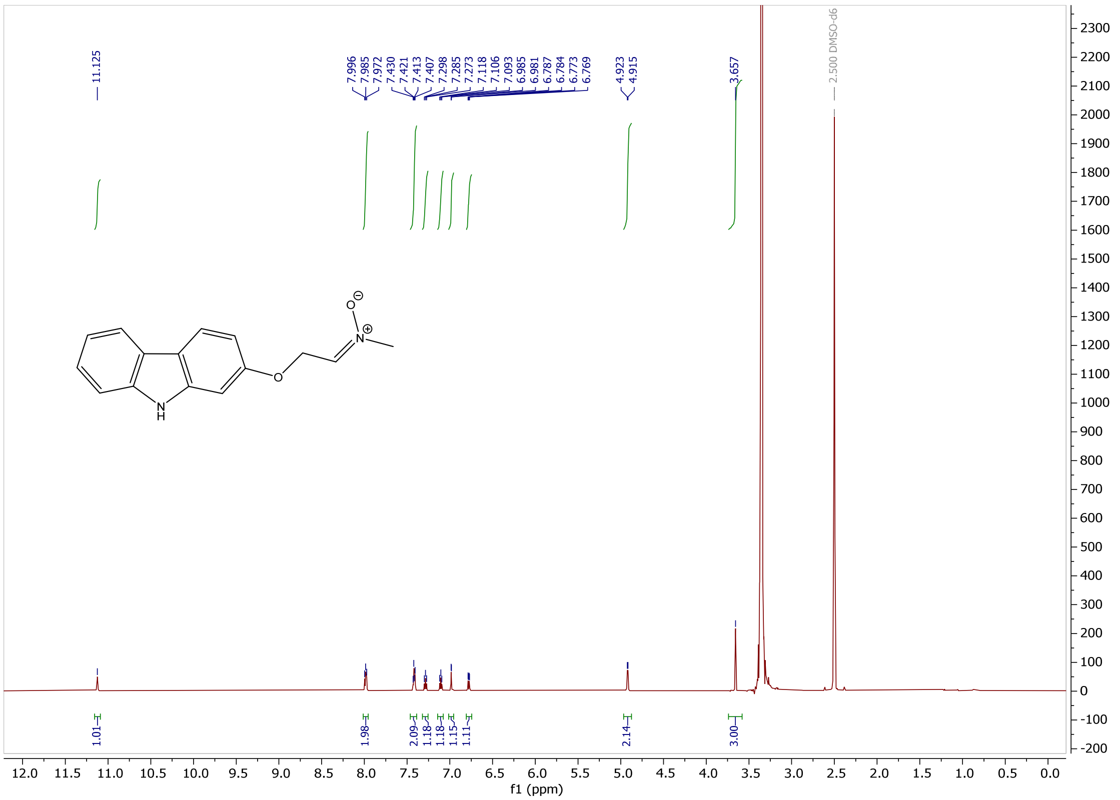

Figure S24: ^{13}C NMR Spectrum for **11ba** in DMSO-d_6.

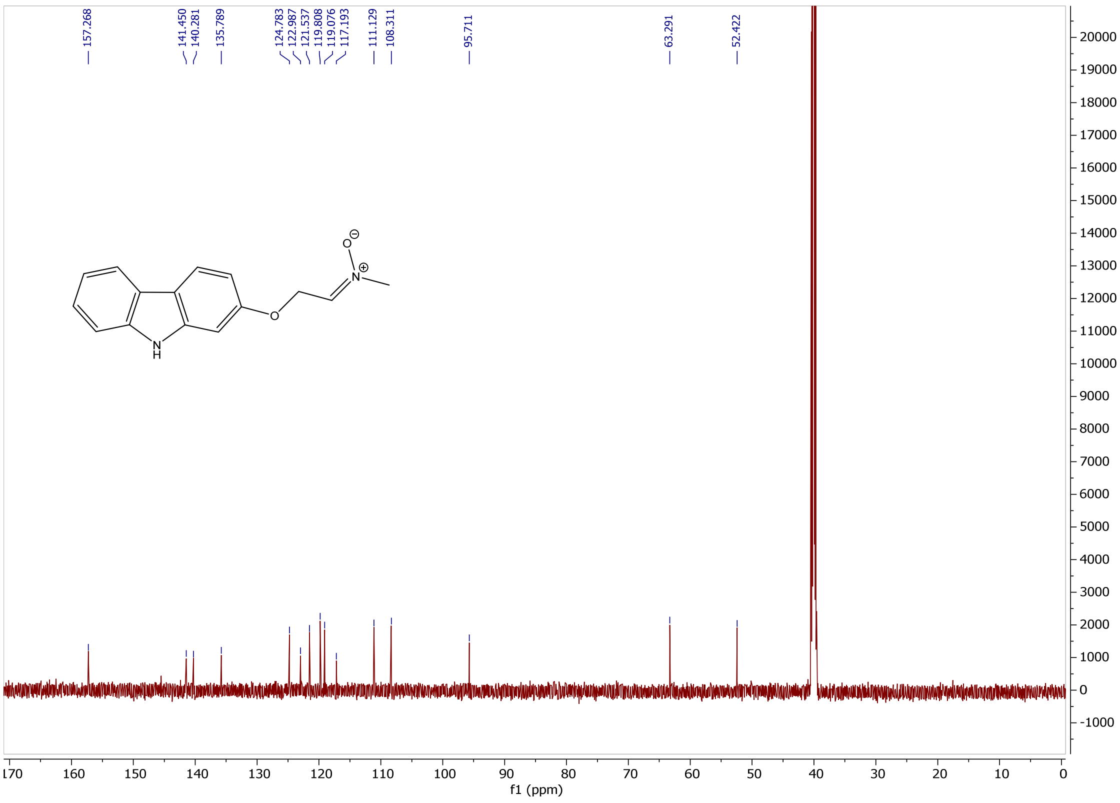

Figure S25: ^{1}H NMR Spectrum for **11bb** in $CDCl_3$.

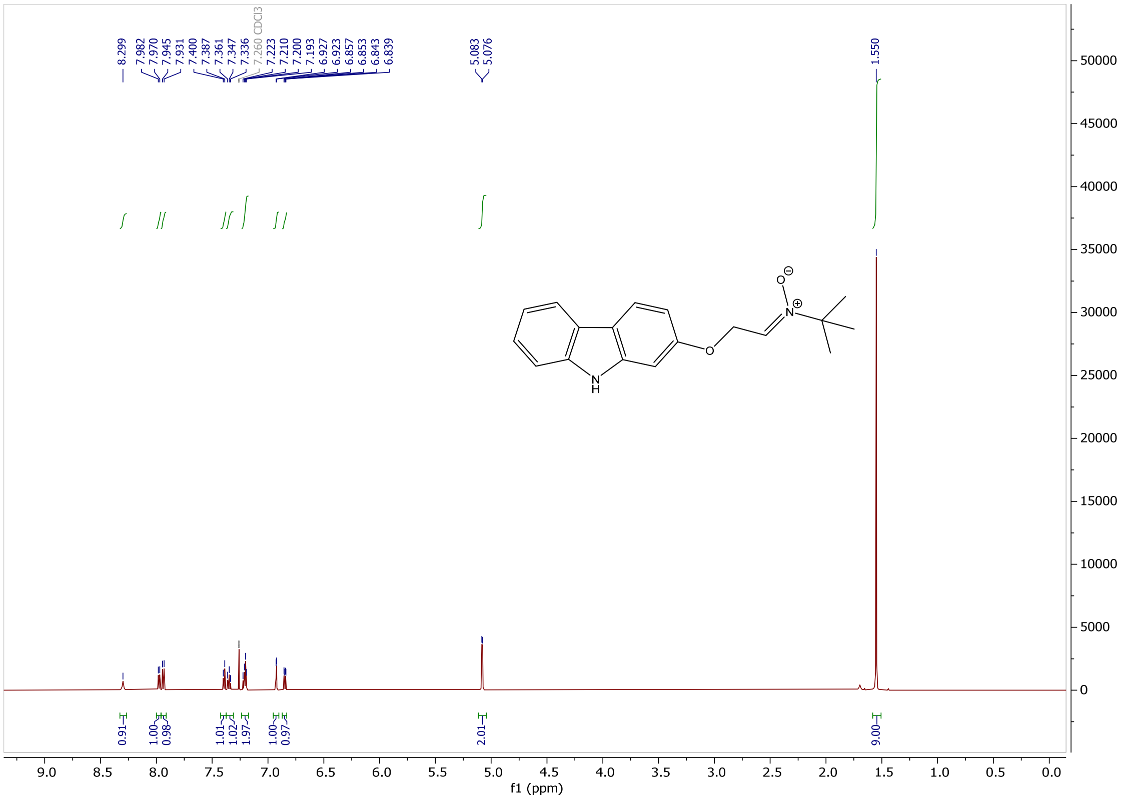

Figure S26: ^{13}C NMR Spectrum for **11bb** in $CDCl_3$.

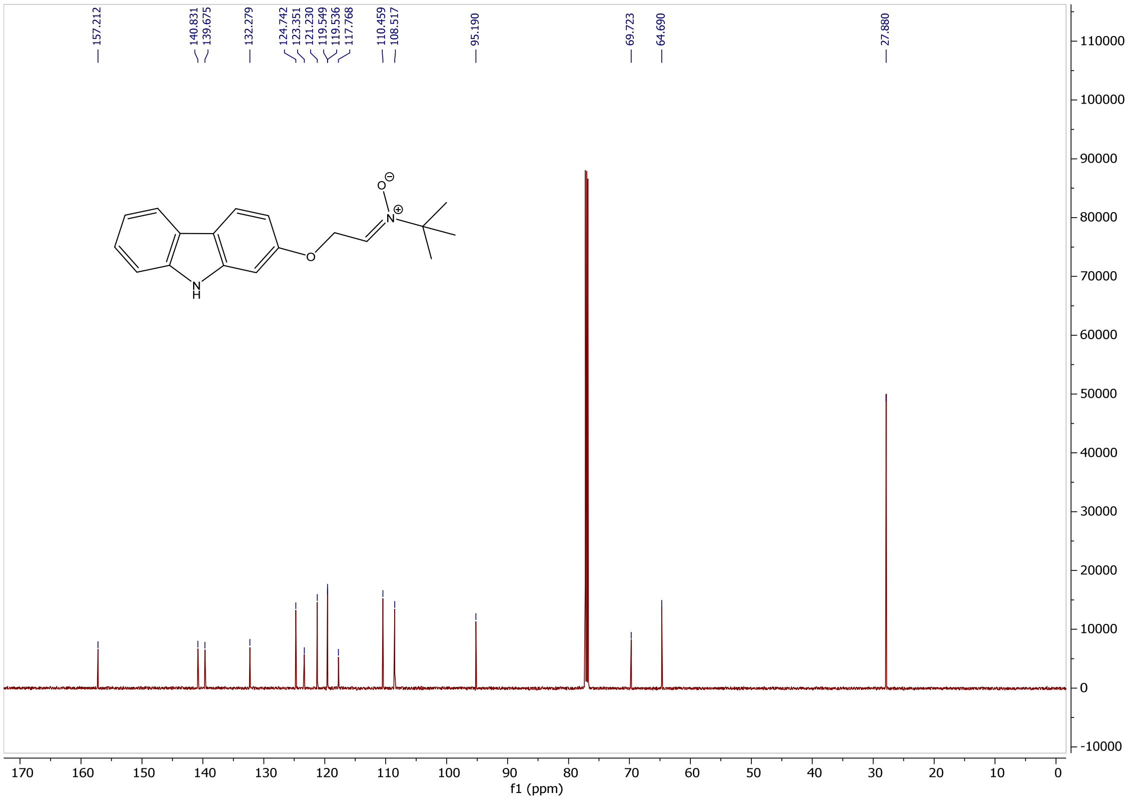

Figure S27: ^{1}H NMR Spectrum for **11bc** in DMSO-d_6.

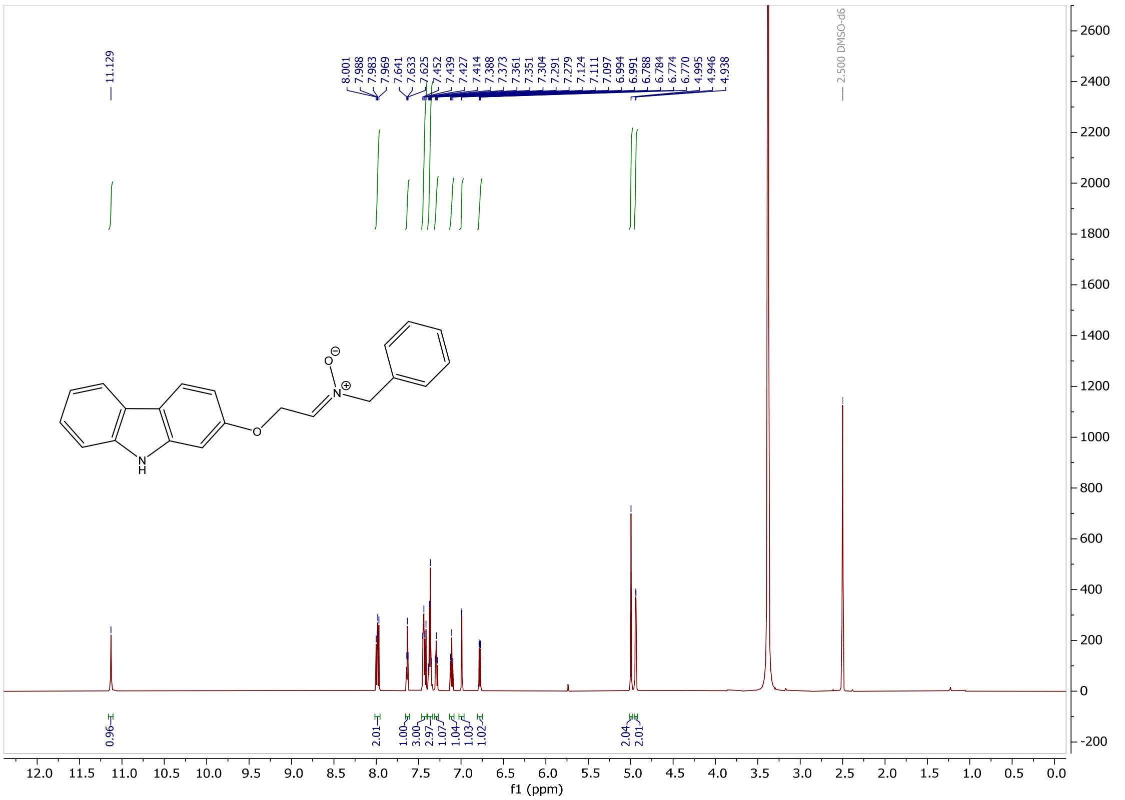

Figure S28: ^{13}C NMR Spectrum for **11bc** in DMSO-d_6.

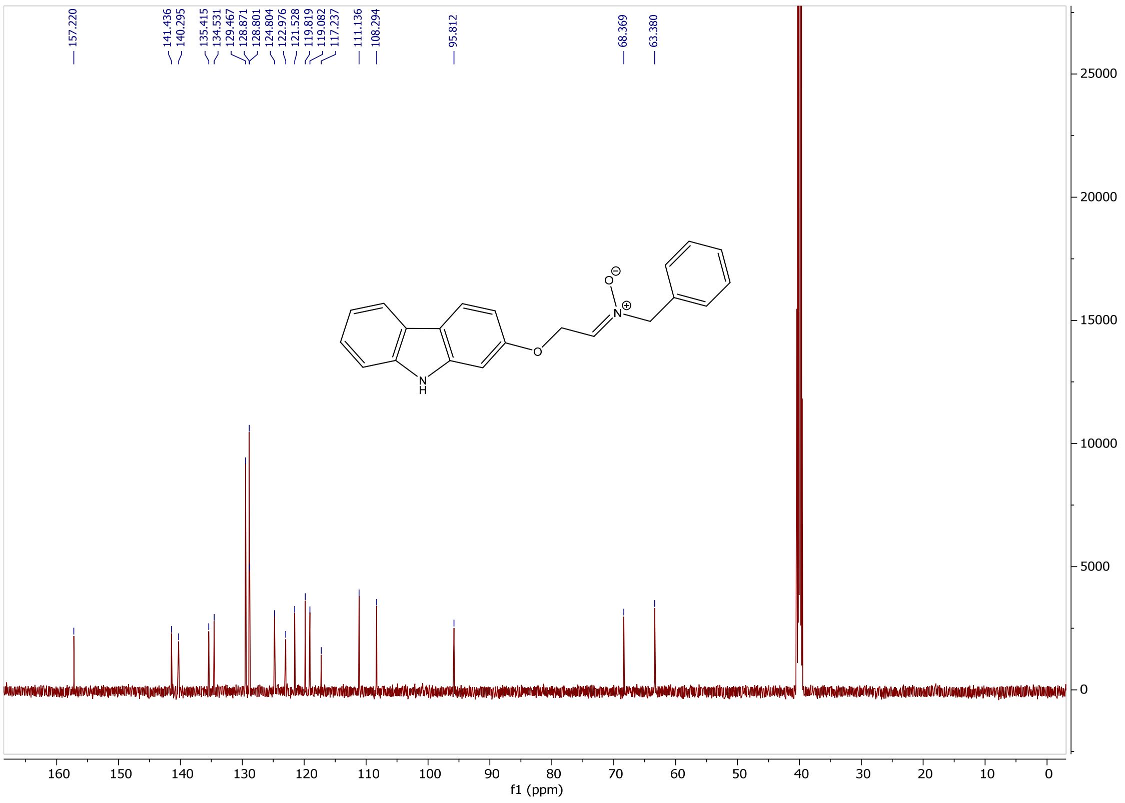

Figure S29: ^{1}H NMR Spectrum for **11bd** in DMSO-d_6.

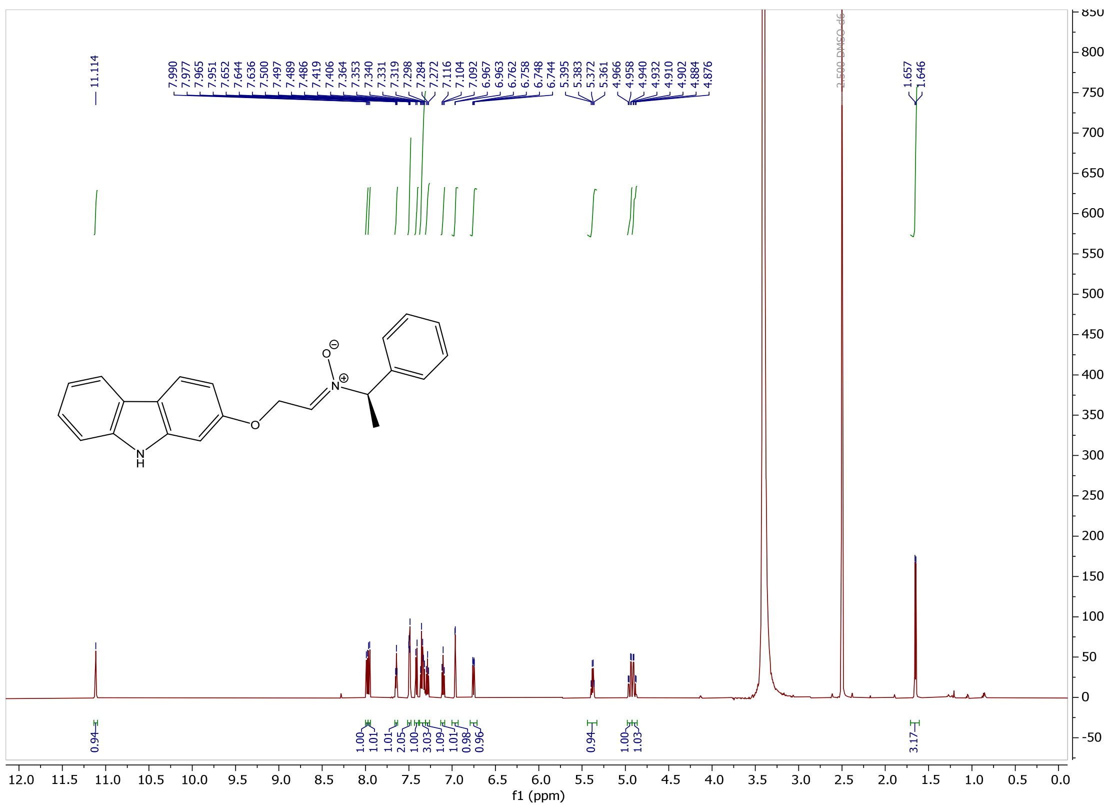

Figure S30: ^{13}C NMR Spectrum for **11bd** in DMSO-d_6.

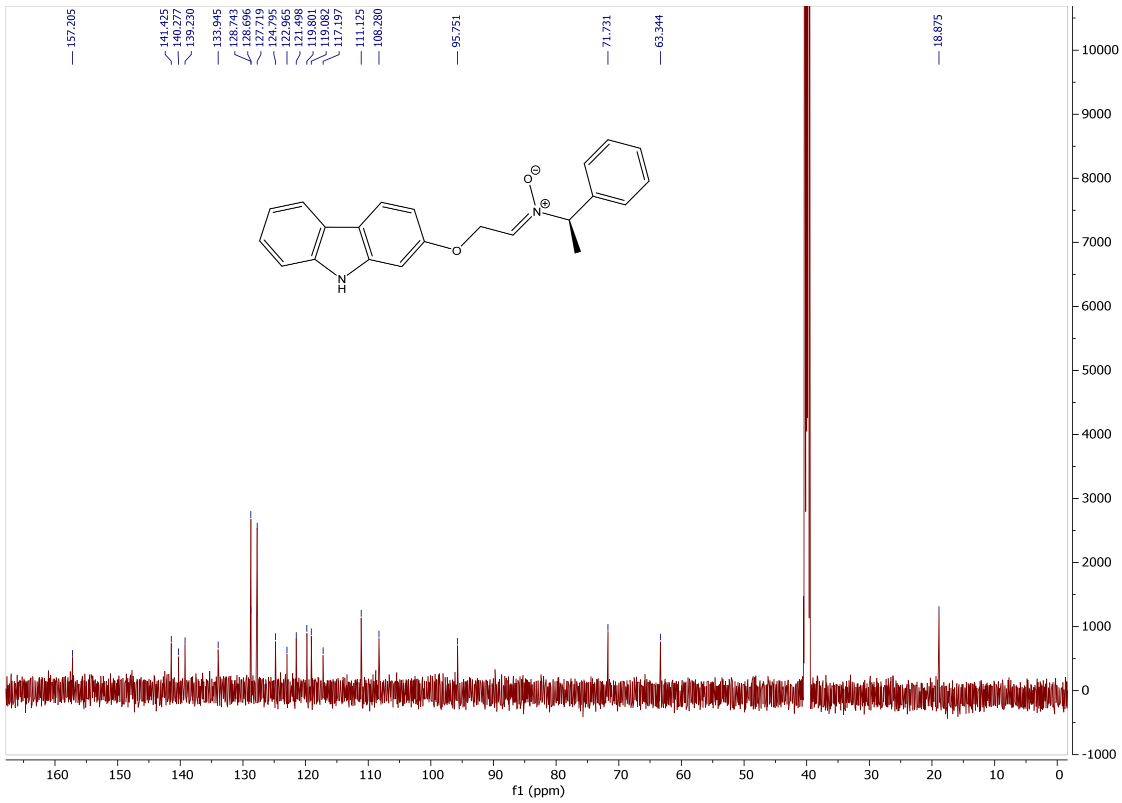

Figure S31: ^{1}H NMR Spectrum for **11be** in DMSO-d_6.

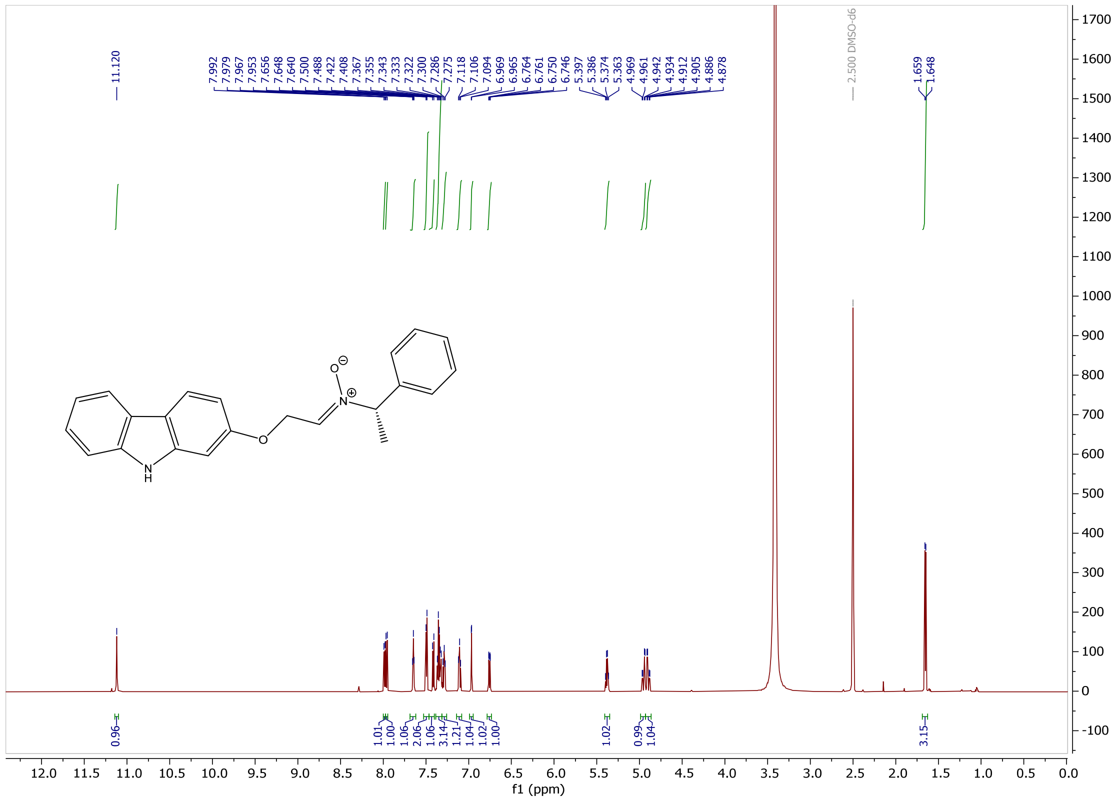

Figure S32: ^{13}C NMR Spectrum for **11be** in DMSO-d_6.

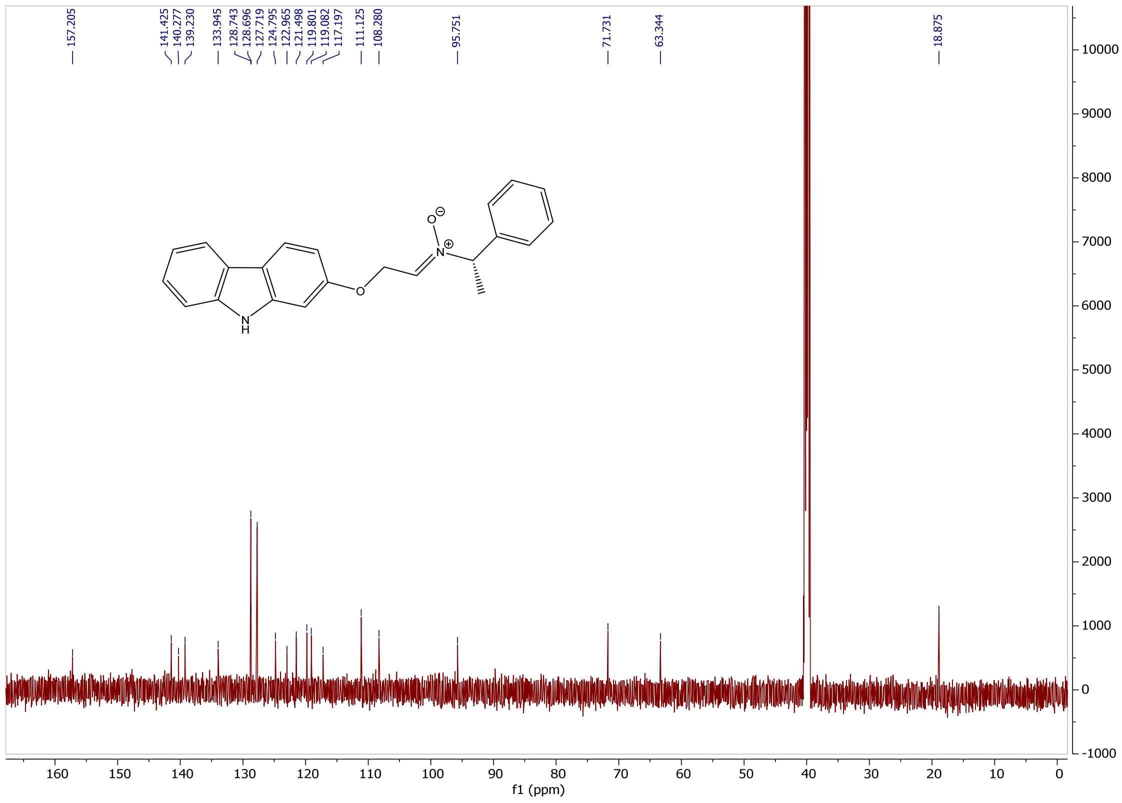

Figure S33: ^{1}H NMR Spectrum for **11bf** in $CDCl_3$.

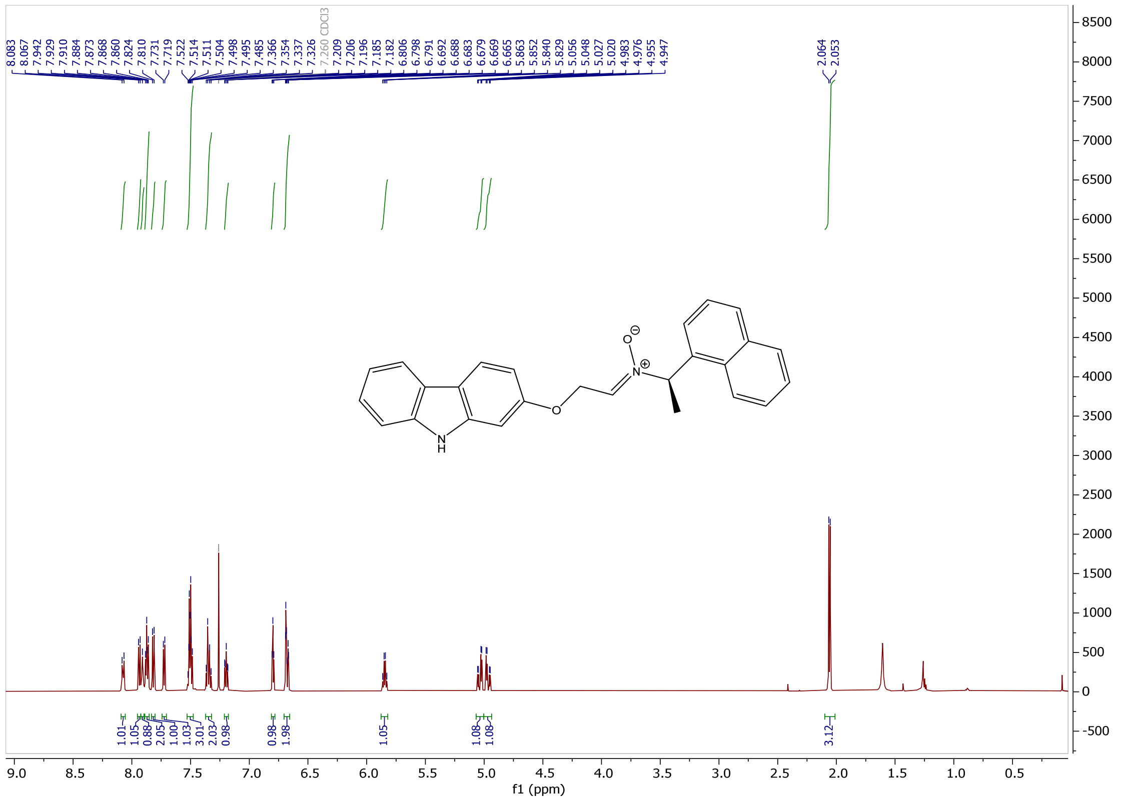

Figure S34: ^{13}C NMR Spectrum for **11bf** in $CDCl_3$.

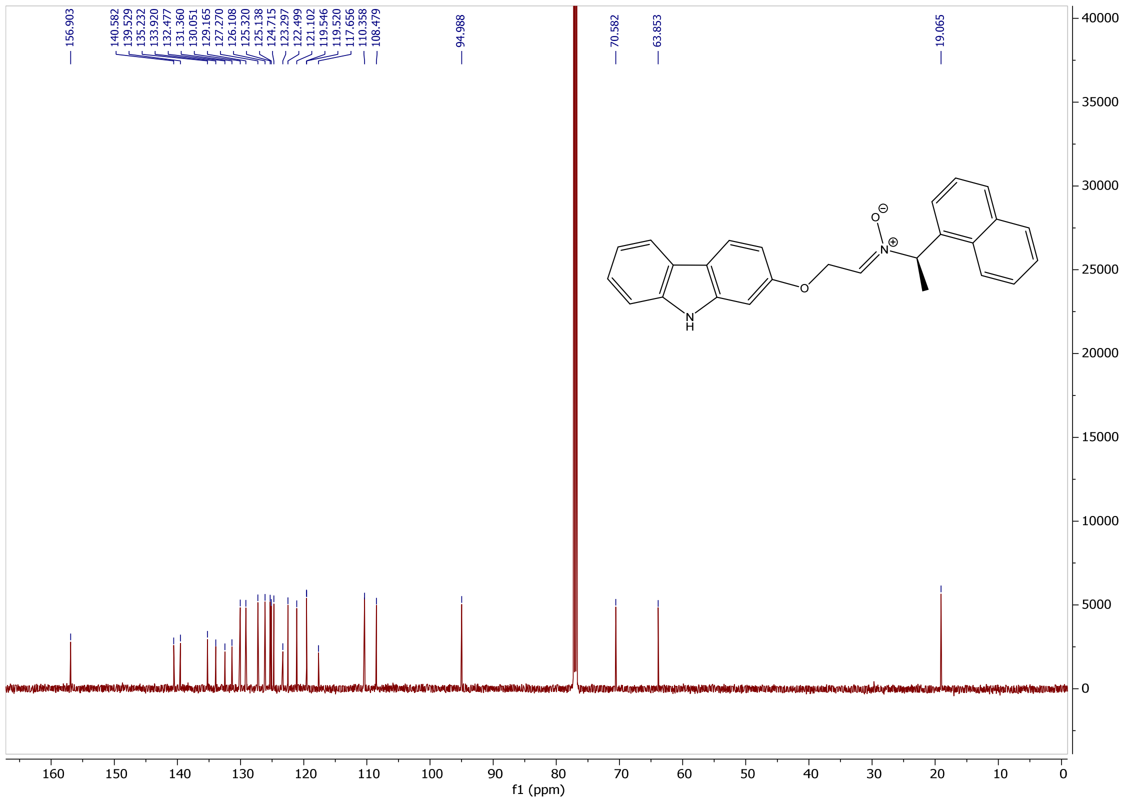

Figure S35: ^{1}H NMR Spectrum for **11bg** in $CDCl_3$.

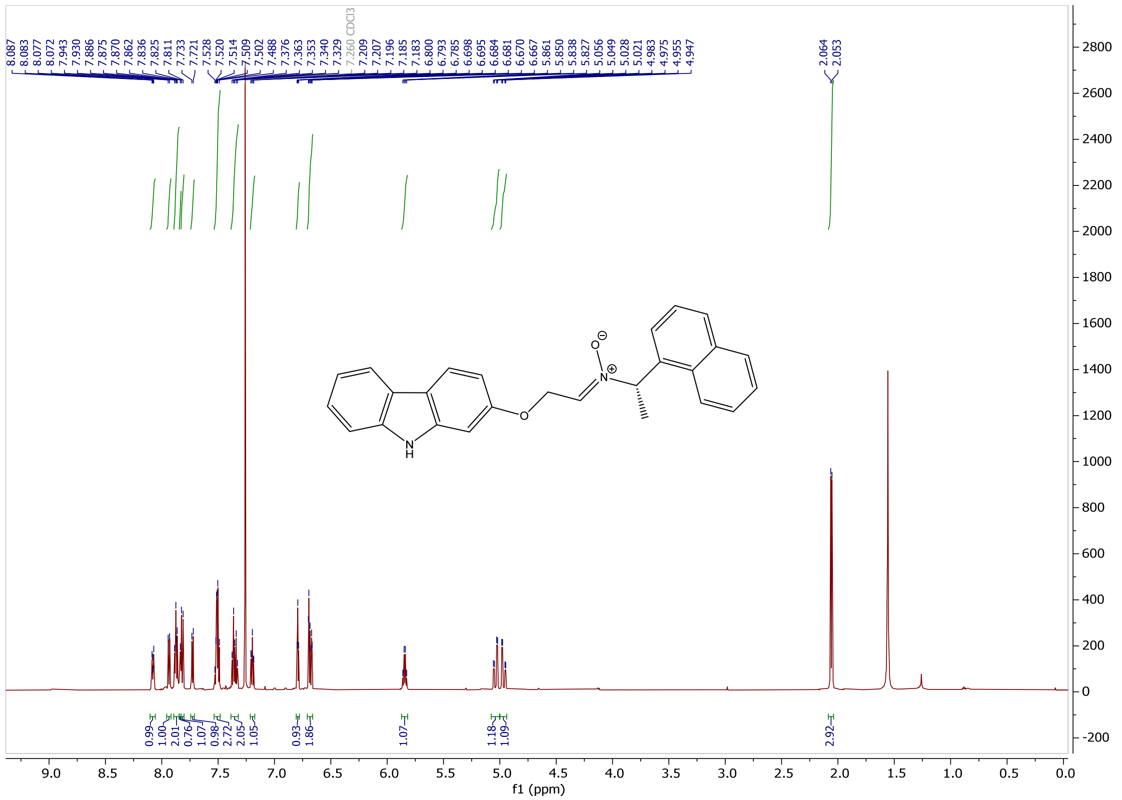

Figure S36: ^{13}C NMR Spectrum for **11bg** in $CDCl_3$.

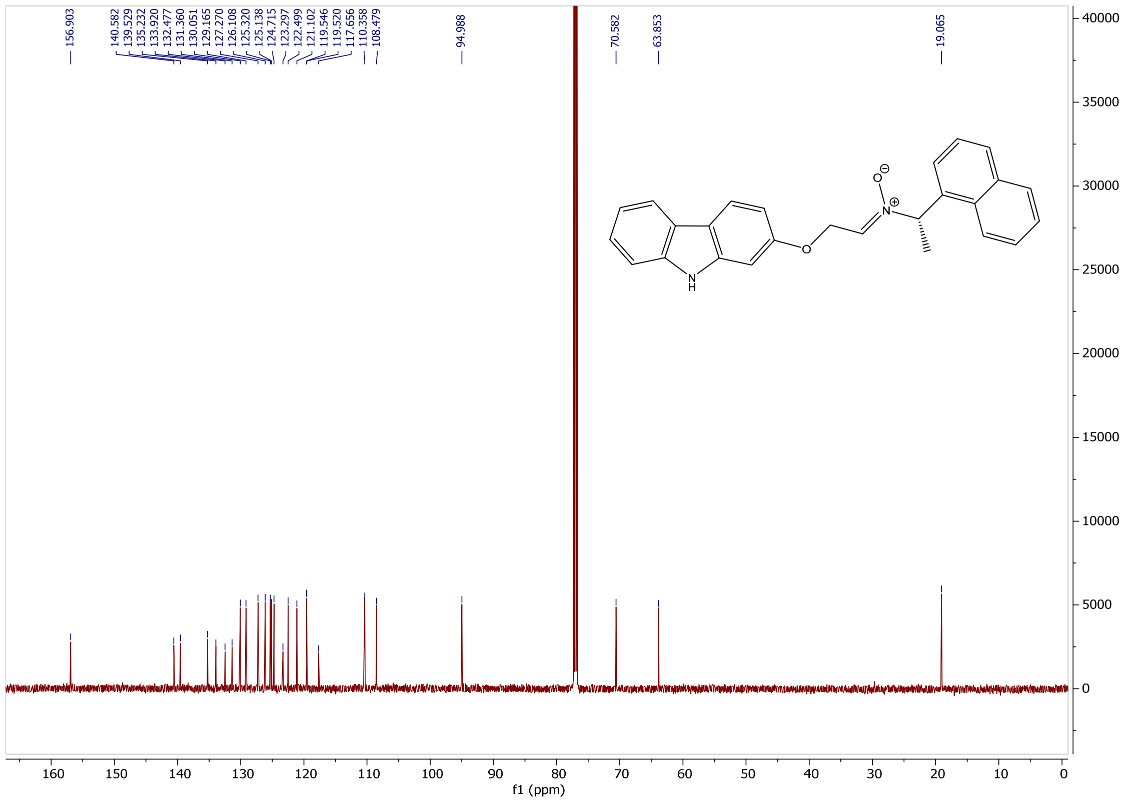

Figure S37: Concentration-response curve for determination of the IC_{50} of nitrone **11aa**.

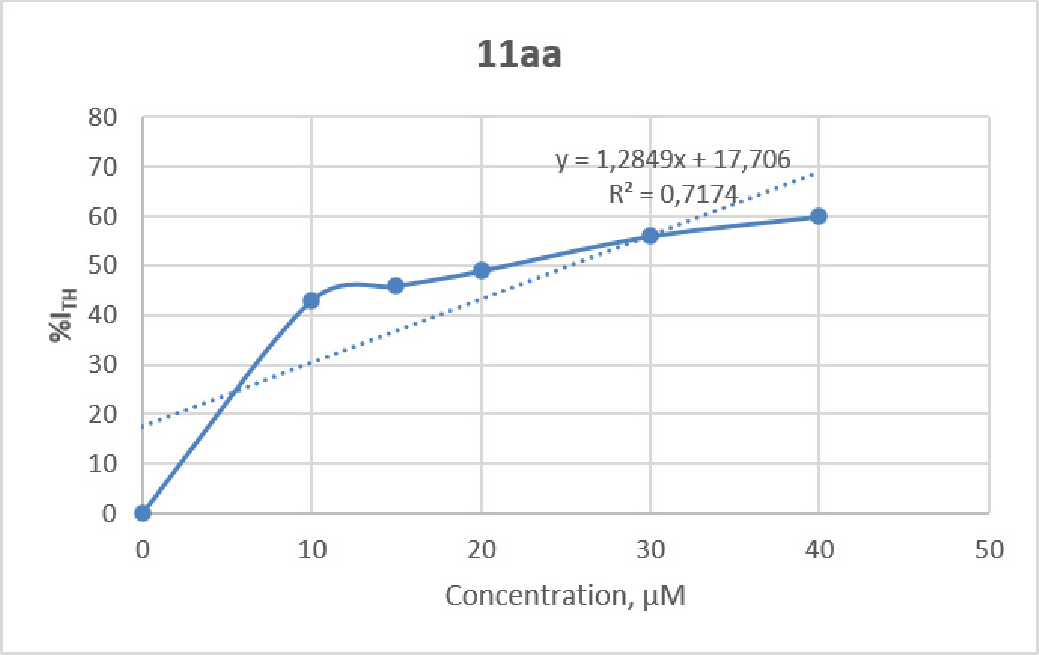

Figure S38: Concentration-response curve for determination of the IC_{50} of nitrone **11ab**.

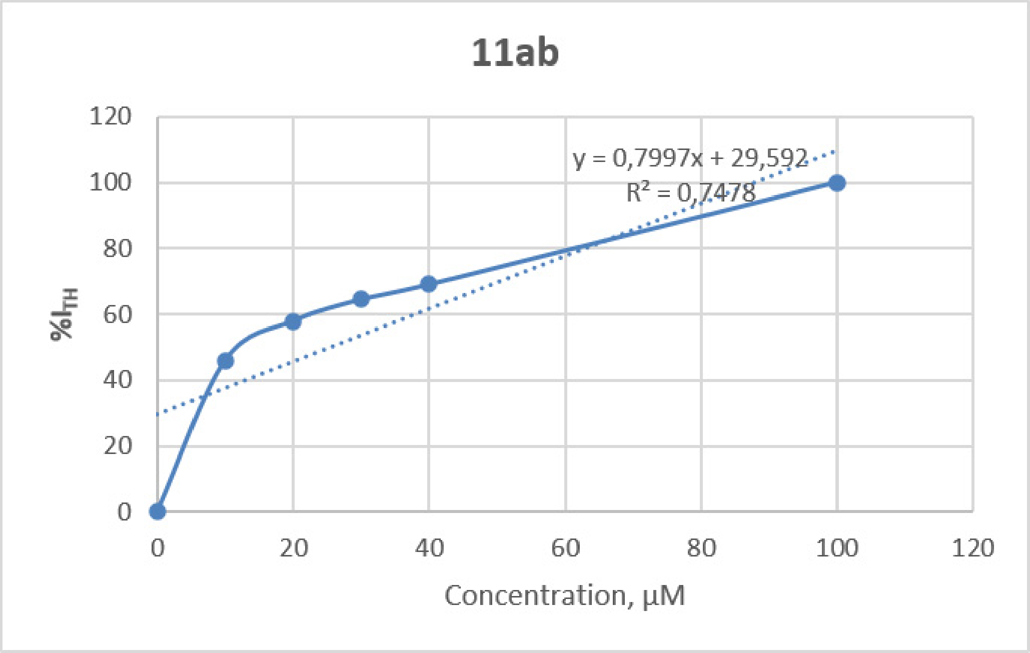

Figure S39: Concentration-response curve for determination of the IC_{50} of nitrone **11ac**.

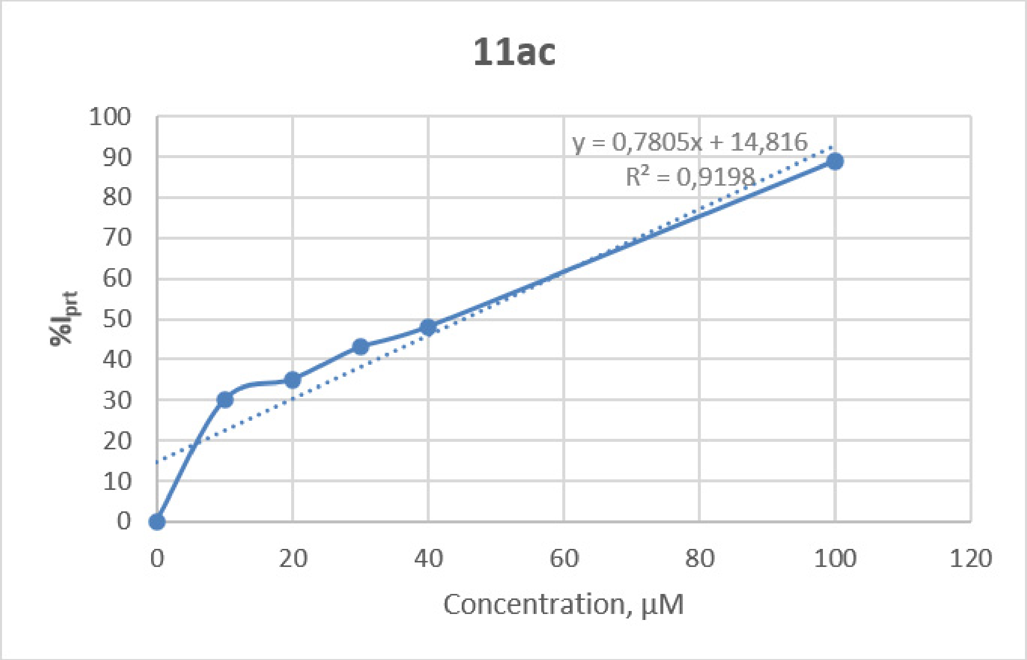

Figure S40: Concentration-response curve for determination of the IC_{50} of nitrone **11ad**.

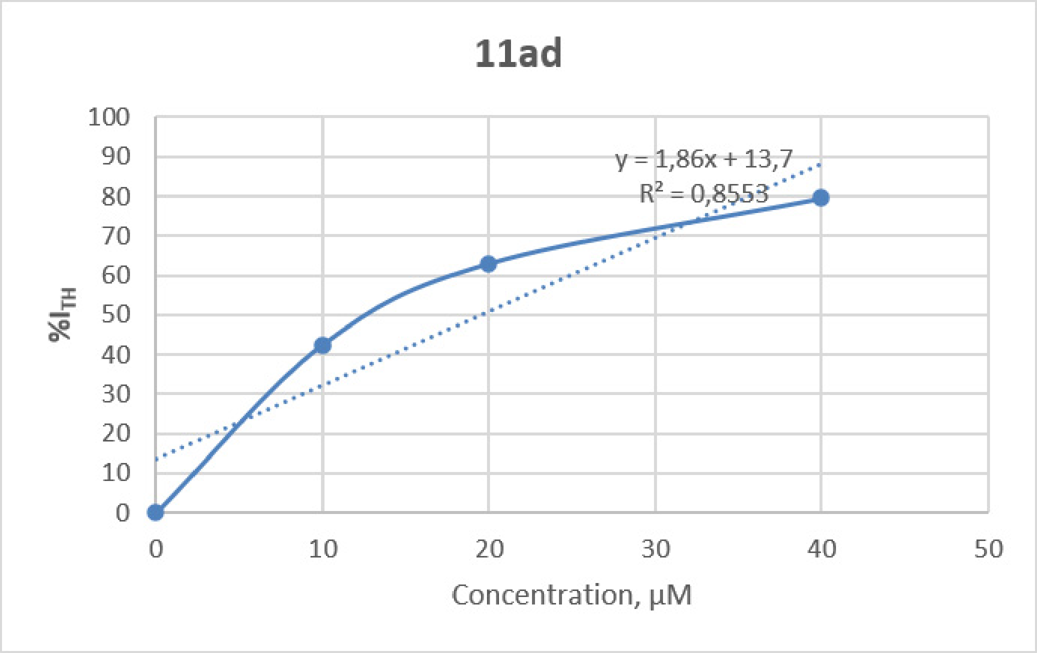

Figure S41: Concentration-response curve for determination of the IC_{50} of nitrone **11af**.

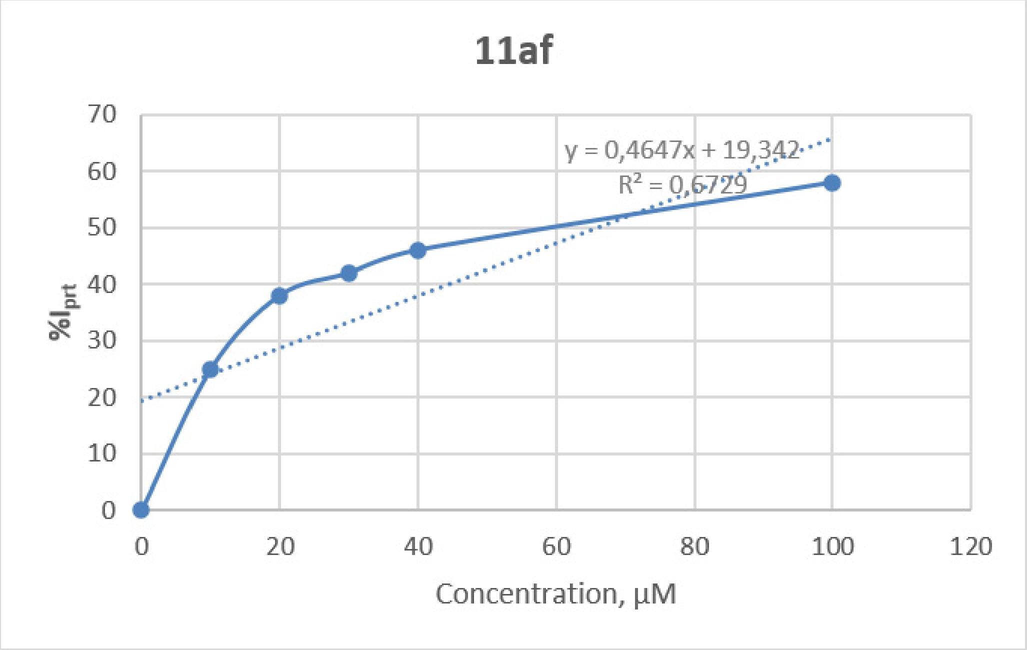

Figure S42: Concentration-response curve for determination of the IC_{50} of nitrone **11ag**.

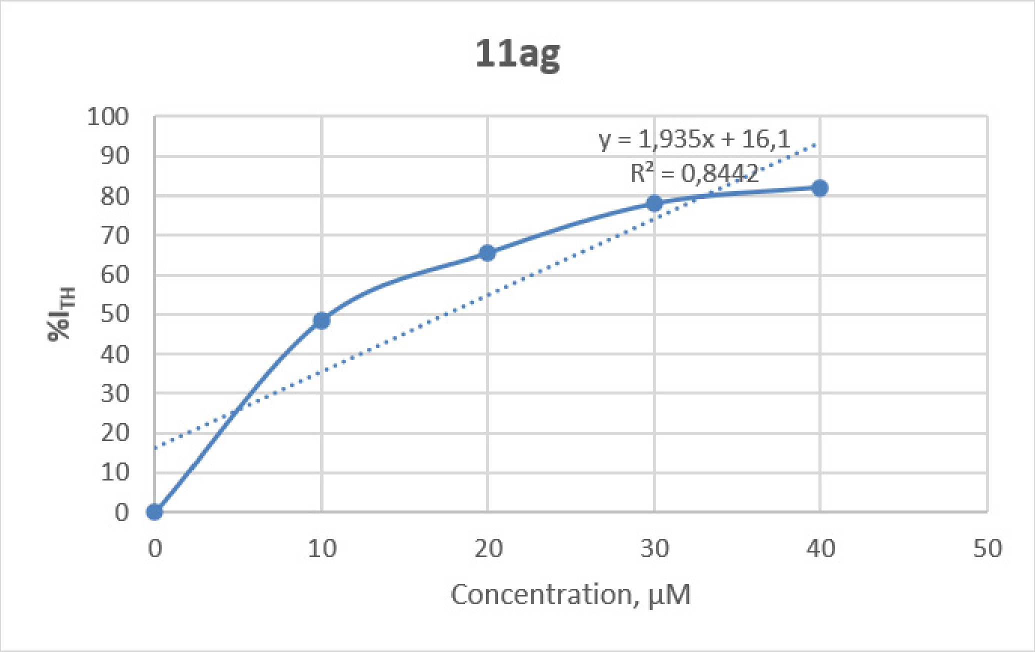

Figure S43: Concentration-response curve for determination of the IC_{50} of nitrone **11ba**.

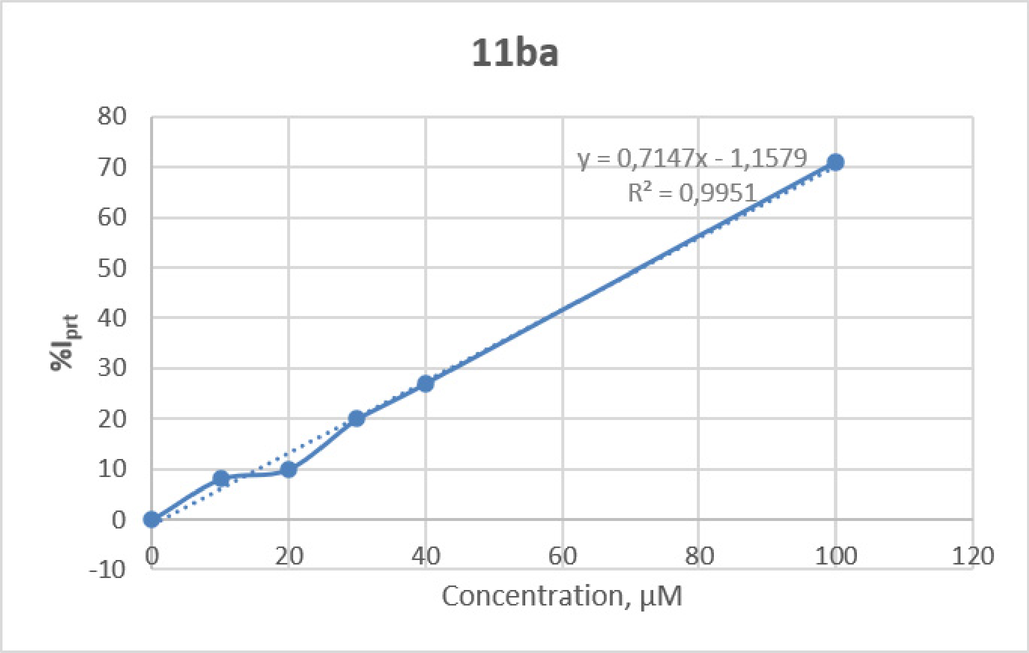

Figure S44: Concentration-response curve for determination of the IC_{50} of nitrone **11bb**.

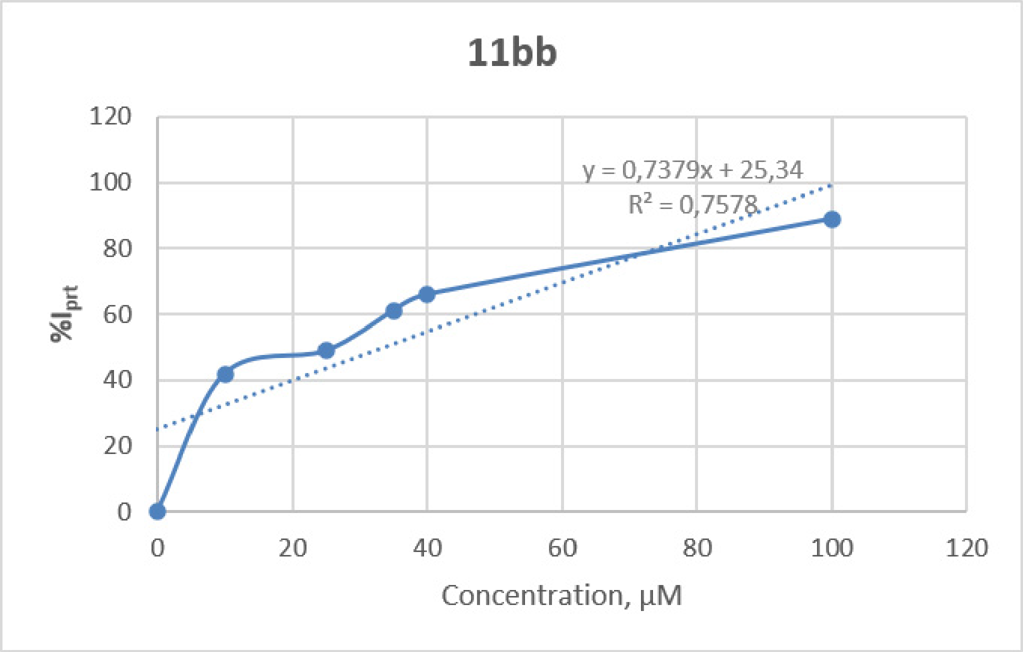

Figure S45: Concentration-response curve for determination of the IC_{50} of nitrone **11bd**.

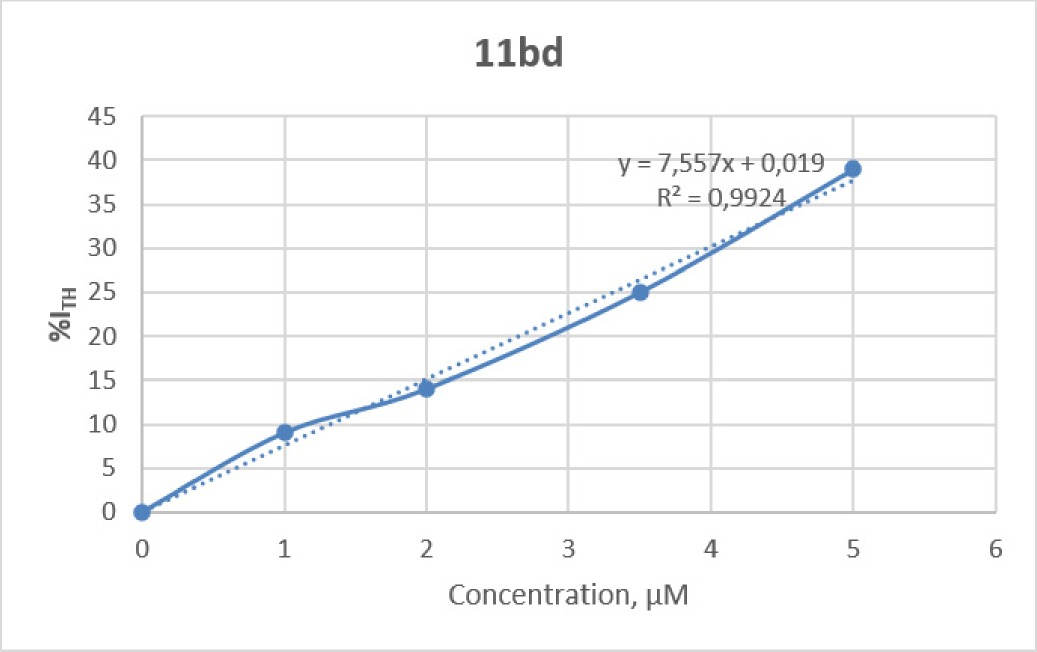

Figure S46: Concentration-response curve for determination of the IC_{50} of nitrone **11be**.

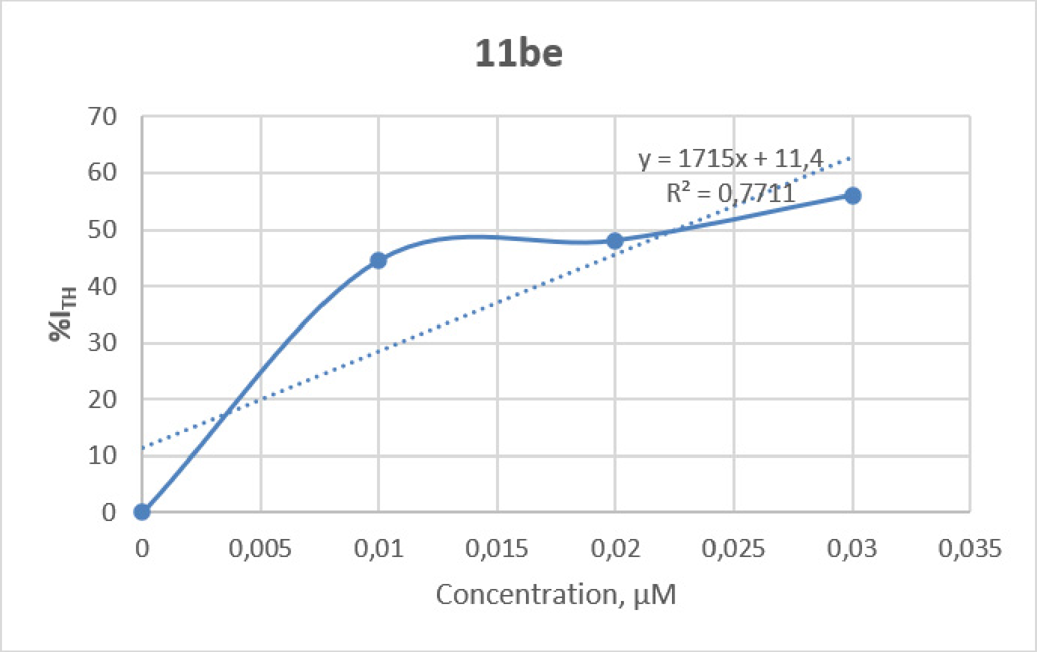

Figure S47: Concentration-response curve for determination of the IC_{50} of nitrone **11bf**.

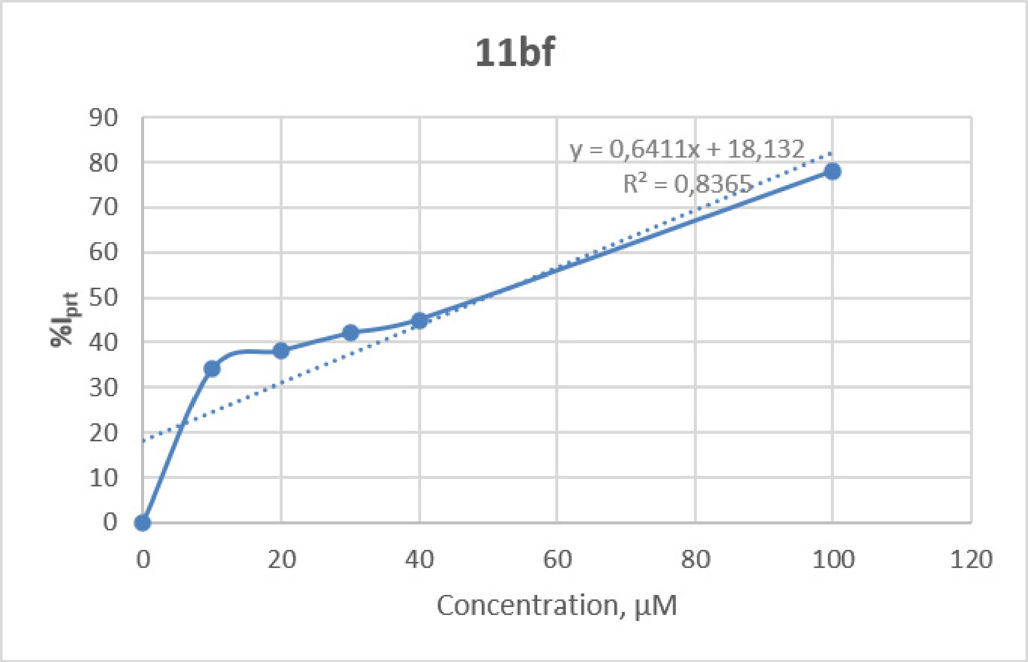

Figure S48: Concentration-response curve for determination of the IC_{50} of nitrone **11bg**.

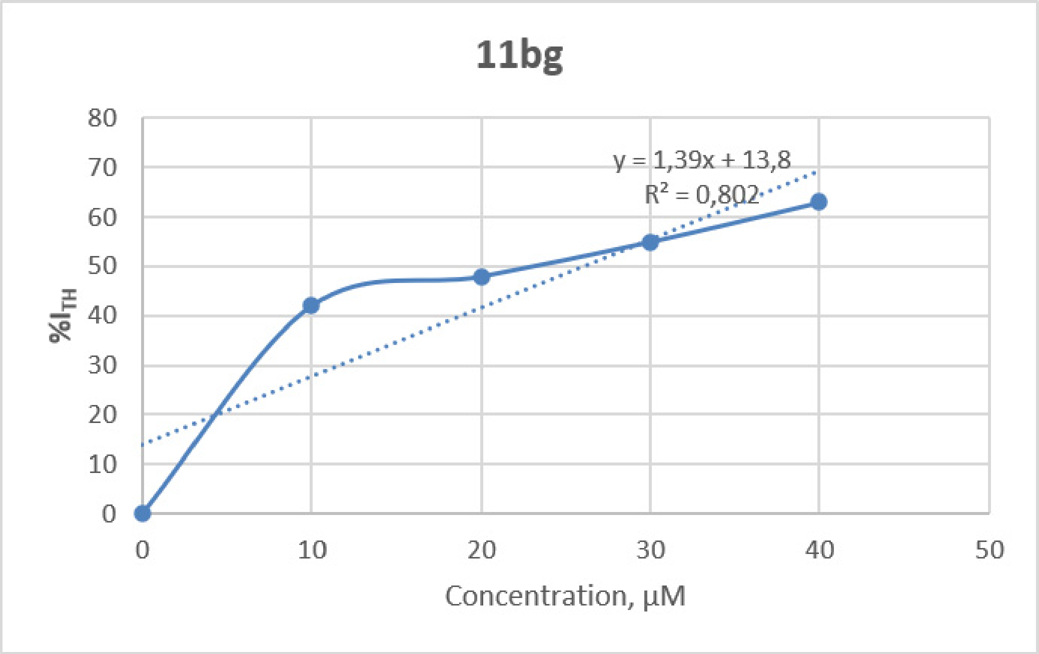

Figure S49: Concentration-response curve for determination of the IC_{50} of NDGA.

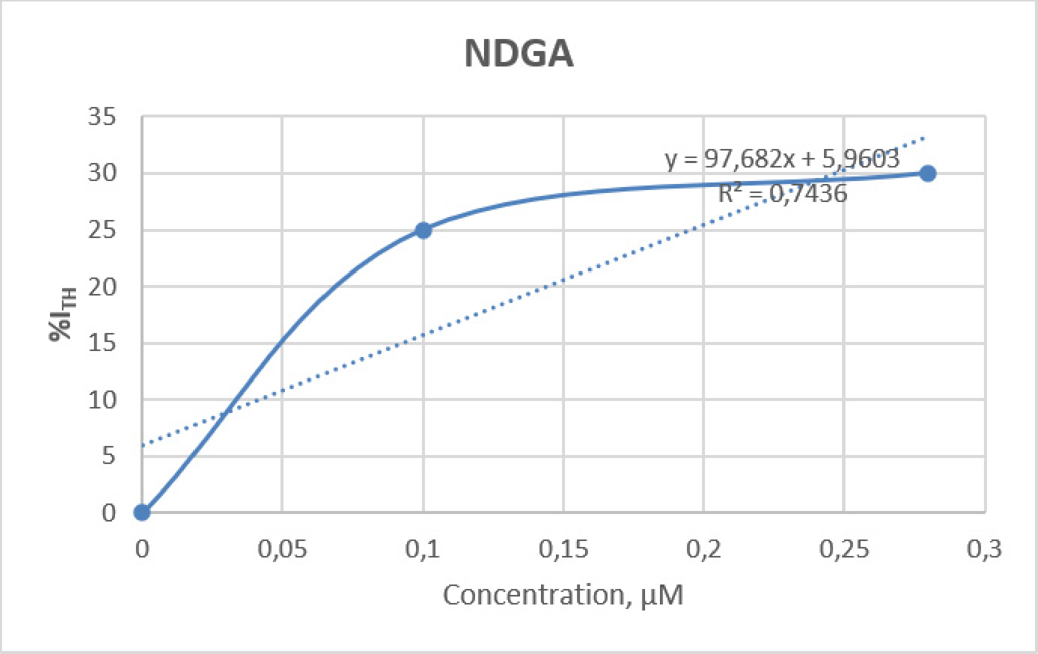

Supplement: Supplementary file 1 [file molecules-31-03619-s001.zip › molecules-4585504-supplementary.pdf]
